# Strength-based capacity-building interventions to promote adolescents’ mental health: A systematic review and meta-analysis

**DOI:** 10.1007/s00787-025-02741-6

**Published:** 2025-05-28

**Authors:** Cong Fu, Wai Tong Chien, Yu Zhang, Kam Ki Lam

**Affiliations:** The Nethersole School of Nursing, Faculty of Medicine, The Chinese University of Hong Kong, New Territories, Shatin, Hong Kong SAR, China

**Keywords:** Strength-based interventions, Mental health literacy, Resilience, Self-efficacy, Adolescents, Capacity-building

## Abstract

Strength-based capacity-building interventions have the potential to enhance an individual’s capacity to improve their mental health. A systematic review and meta-analysis was conducted to examine the effectiveness of these interventions on mental health literacy, resilience, self-efficacy, and positive thinking in adolescents without pre-existing mental/psychiatric diagnoses/conditions. CINAHL, Embase, PsycINFO, PubMed, Web of Science, the Cochrane Library, and CNKI were searched from inception to June 2024. Two reviewers independently conducted the study screening, risk of bias assessment, and data extraction. Leave-one-out sensitivity and subgroup analyses were used to reduce the heterogeneity of the studies and their findings. Forty-seven interventional studies were included, with approaches mainly consisting of psychoeducation, individual skill training (e.g., emotional regulation, mindfulness), and supportive peer feedback. Capacity-building interventions (in 27 studies) improved the levels of mental health literacy (*SMD* = 1.70, 95% *CI*[0.57, 2.84], *I*^*2*^ = 97%, n = 4) and resilience (*SMD* = 0.51, 95% *CI*[0.26, 0.76], *I*^*2*^ = 89%, n = 16) in the short-term (< 1 month) and resilience in the long-term (> 6 months; *SMD* = 0.29, 95% *CI*[0.03, 0.55], *I*^*2*^ = 39%, n = 3) post-intervention. While there were limited studies evaluated on positive thinking and self-efficacy and thus unable to perform meta-analysis on these outcomes, the medium-term effects on resilience show no statistical significance (1–6 months; n = 4). However, the effects on depression and anxiety were nonsignificant. Combined approaches to capacity-building interventions that incorporate two or more theoretical frameworks/models within one intervention could enhance adolescents’ resilience (*SMD* = 0.24, 95% *CI*[0.13, 0.35], *I*^*2*^ = 22%, n = 3). Combining positive psychology with other mental health approaches in interventions might boost adolescents' resilience in short-term but there were limited studies and thus insufficient evidence to evaluate the medium- and long-term effectiveness of these combined approaches. Further rigorous large-scale randomized controlled trials are recommended to provide stronger evidence.

**Supplementary Information:**

The online version contains supplementary material available at 10.1007/s00787-025-02741-6.

## Introduction

Adolescence, the stage of human development between ages 10 and 19 according to the World Health Organization [1–3], is marked by a period of significant adaptive plasticity and critical opportunities for positive growth [4]. During this period, adolescents can explore the world, acquire competencies, learn to navigate life challenges, and ultimately become independent and productive members of society [5, 6]. The World Health Organization (2022) emphasizes the importance of mental health promotion and illness prevention programs to enhance adolescents’ abilities to regulate their emotions, develop alternatives to risk-taking behaviors, build resilience, and establish supportive social environments and networks. Alarmingly, global statistics indicate that 10% to 20% of adolescents experience some level of mental health problems [7, 8]. Such conditions can persist into adulthood, leading to cognitive impairments, educational challenges, and a diminished quality of life [9, 10]. Adolescence is thus a critical period for developing social and emotional habits foundational to mental well-being.

Research on adolescent development has suggested that focusing on capacity-building and providing enabling environments—rather than emphasizing deficits—can substantially enhance adolescents’ transition to adulthood and their overall mental health [11]. For instance, the Positive Youth Development framework advocates for fostering competencies, confidence, connections, character, and caring among adolescents. Additionally, ecological systems theory posits that adolescents thrive in nurturing environments, which include supportive families, schools, and communities [12]. These theories underscore the importance of recognizing adolescents’ individual capacities for decision-making and responsibility in promoting mental health and healthy development. Building upon these foundational concepts, recent interventions have adopted a more holistic approach to adolescent well-being, with a shift from focusing on deficits to a more positive strength-based perspective that emphasizes capacity-building to better understand and support their healthy development and mental health [13, 14]. Rooted in the field of social work, the strength-based approach empowers individuals by recognizing and nurturing their strengths and potential, facilitating positive life changes [15]. Interventions based on this framework have demonstrated effectiveness in enhancing healthcare capabilities, training mental health professionals, and developing community support networks [16–19]. Mental health literacy (MHL) is a cornerstone of strength-based interventions, equipping individuals with the knowledge to recognize and manage mental health challenges, thereby empowering them to seek help and engage proactively in their care [20]. Building on one’s mental health literacy, resilience enables an individual’s adaptive coping with adversity by leveraging internal and external resources, while self-efficacy—the belief in one’s ability to enact health-promoting behaviours—serves as a critical motivator for sustained action. Strength-based interventions explicitly target these capacities (i.e., promoting resilience and self-efficacy) to enhance adaptive functioning and reduce vulnerability to stressors [3]. Finally, positive thinking, operationalized through cognitive reframing and learned optimism, shifts focus from speculative risks to constructive outcomes, strengthening overall psychological and emotional well-being, as well as further enhancing resilience [3, 21]. These four capacities (MHL, resilience, self-efficacy, and positive thinking) form a conceptual basis and targeted outcomes that aligns with the core principles of strength-based approaches, including empowerment, proactive mental health management, and sustained well-being [22]. Although research has explored strength-based interventions for adolescents with specific conditions such as autism spectrum disorders, there is a notable lack of studies addressing the mental health needs of adolescents among the general population. To address this gap, the present review focused on four key capacities (***MHL, resilience, self-efficacy,**** and ****positive thinking***) as essential components based on established evidence supporting their impact on adolescents’ mental health. Each of these four capacities and their operational focuses were examined in this review (elaborated below) to provide a comprehensive framework for understanding and assessing capacity-building interventions for adolescent mental health.

### Mental health literacy

Mental health literacy is critical for adolescent mental health. Defined as the ability to recognize mental health issues, understand their influencing factors, and how to seek help and support, mental health literacy encompasses not only mental health-related knowledge but also the skills and attitudes necessary to reduce stigma and foster a supportive environment for promoting mental health [23–25]. Research has indicated that higher levels of mental health literacy are associated with better mental health outcomes among adolescents, while low mental health literacy is correlated with increased risks of depression and negative mental health outcomes [26, 27]. Importantly, improving mental health literacy can enhance psychological resilience and self-efficacy, allowing adolescents to approach challenges with competence and confidence. This multifaceted construct plays a crucial role in recognizing mental health-related knowledge, developing skills and strategies for seeking help, fostering positive attitudes, reducing the stigmatization of mental health issues, and maintaining positive mental health.

### Resilience

Resilience is defined as an individual’s ability to adapt and recover when facing adversity or constraints [28, 29]*.* This adaptive capacity is essential not only in navigating significant life challenges but also in handling everyday stressors, such as academic demands [30]. Empirical evidence indicates that resilience significantly mitigates psychological distress [31] and fosters positive growth amidst developmental changes and life challenges [32]. Galatzer-Levy's trajectory research on resilience demonstrates that resilience is an outcome characterized by successful coping and adaptation in the face of adversity [33]. Kalisch's neurobiological study conceptualizes resilience as a dynamic process driven by subjective appraisal mechanisms, highlighting the interplay between cognitive evaluation and adaptive responses [34]. Resilience is therefore a fundamental mechanism in promoting positive adaptation and mitigating adverse mental health outcomes among adolescents, making it a crucial factor for their overall well-being and development [30]. Initially focused on risk and adversity, resilience theory emphasizes the recognition of individual capacities for growth rather than focusing on deficits alone [35]. Capacity-building interventions based on resilience theory seek to promote personal strengths and contribute to healthy adolescent development [13, 36]. Recent scholarship has advocated for equipping adolescents with developmentally appropriate information about mental health to enable them to better navigate life challenges [23]. A critical component of mental health is the capacity to effectively manage life events, including personal challenges, stress, and interpersonal conflicts [37]. Resilience is not a static trait but a malleable quality that can be cultivated and strengthened. This malleability suggests that targeted interventions to enhance adolescents’ resilience can improve their coping mechanisms for managing adversity and stress, thereby reducing the negative psychological impacts they may experience.

### Self-efficacy

Self-efficacy, defined as the belief in one’s capability to execute necessary actions, is fundamental to achieving one’s desired developmental and mental health outcomes. Perceived self-efficacy plays a crucial role in shaping adolescents’ ability to navigate various life challenges, such as academic achievement, prosocial behavior, and major life events [38]. Positive recognition and feedback from others during these challenges can reinforce adolescents’ sense of competence and strengthen their self-efficacy [39]. Adolescence is a critical period of exploration and skill development. Fostering self-efficacy during this period is crucial, as it empowers young individuals to navigate life’s challenges confidently and effectively. Studies have consistently identified self-efficacy as a significant health-promoting factor and as a key capacity for adolescent mental health [40, 41]. Studies have shown that adolescents with higher levels of self-efficacy tend to exhibit more positive thinking patterns and greater life satisfaction [42, 43]. Conversely, those with lower self-efficacy often struggle to maintain positive thinking, largely because of heightened emotional sensitivity to negative stimuli [44]. However, recent adolescent health studies have revealed inconsistencies in the definitions and essential components of strength-based capacity-building interventions within the resiliency framework [41]. This variability may stem from unclear theoretical foundations and operationalization of the concept of capacity-building, resulting in limited evidence regarding the design and effectiveness of such interventions [16, 45, 46].

### Positive thinking

Positive thinking has been defined as having an optimistic perception that aids in problem-solving and fosters a positive outlook on the future. It encourages adolescents to take proactive actions, which can lead to better mental health outcomes [47, 48]. Conversely, adolescents who are not able to think positively may have less ability to cope with everyday stress and be more likely to experience depressive symptoms [49]. A high level of positive thinking is also related to reduced distress and improved overall psychological well-being [50].

These capacities, individually or in combination, can be considered core variables underlying human strengths to overcome obstacles. By synthesizing these capacities, this systematic review and meta-analysis evaluated the effectiveness of interventions for enhancing adolescents’ individual capacities and identifying specific therapeutic components or elements of the capacity-building interventions that have been found effective for promoting mental health.

## Methods

We pre-registered this meta-analysis protocol in PROSPERO (CRD42023474504) and followed the PRISMA statements [51].

### Search strategy

We identified peer-reviewed articles on interventions that jointly targeted adolescent mental health by searching CINAHL, Embase, PsycINFO, PubMed, Web of Science, the Cochrane Library, and CNKI (Chinese Knowledge Infrastructure) from their inceptions to June 2024. The search terms were developed using the population, intervention, comparator, and outcome framework. We screened citations from the included studies, traced them in Scopus, searched academic and online search engines (particularly Google Scholar and Microsoft Academic), and checked major clinical trial registries, including ClinicalTrials.gov and the International Standard Randomized-Controlled Trial Number (ISRCTN) Registry, to identify additional relevant studies. The full search strategy is shown in Supplementary Information (SI) 1.

### Inclusion and exclusion criteria

Studies were included if they (1) involved adolescents aged 10 to 19 years [4]; (2) were an interventional study; (3) used an intervention targeted at enhancing adolescent capacities to maintain and promote mental health in areas of mental health literacy, resilience, self-efficacy, and/or positive thinking; and (4) included a control or a comparison group, which involved either no intervention, waitlist control, or active controls that did not specifically target adolescent mental health literacy, resilience, self-efficacy, and/or positive thinking. The studies were carried out in non-clinical settings, including schools, communities, and families. Studies published in both Chinese and English were included. Studies were excluded if they involved adolescents who had pre-existing clinically diagnosed mental health disorders, cognitive impairments and/or learning disabilities; adolescents who were receiving or had recently received psychiatric and/or psychological treatments; and adolescents with serious physical illnesses at recruitment.

### Study selection

We imported the relevant articles into Covidence (www.covidence.org) [52] for screening, where duplicates were automatically removed. CF and WTC independently selected the articles, resolving disagreements through discussion. When necessary, SKKL was consulted to achieve consensus.

### Data extraction

A structured form was developed for data extraction following the Cochrane Consumers and Communication Review Group’s guidelines [53]. The form consisted of (1) authors, (2) year of publication, (3) study country/location, (4) study design and setting(s), (5) participants, (6) interventions, (7) outcome measures, and (8) main results. CF and SKKL independently performed data extraction, resolving discrepancies through discussion. All the authors checked the extracted data for accuracy.

### Assessment of risk of bias in the included studies

The risk of bias in the included studies was independently evaluated by two reviewers, CF and WTC, using Version 2 of the Cochrane risk of bias tool (RoB 2) [54] for randomized controlled trials (RCTs) and cluster randomized controlled trials (c-RCTs). The quasi-experimental design was assessed using Risk Of Bias In Non-randomized Studies – of Interventions (Robins-I) [55]. The third reviewer, WTC, was consulted when necessary. The RoB 2 tool assessed five domains: (1) bias arising from the randomization process, (2) deviations from intended interventions, (3) missing outcome data, (4) measurement bias of the outcome, and (5) bias in the selection of reported results. The studies were categorized as having low, moderate, or high risk of bias. In addition, c-RCTs were assessed for other potential bias using the Cochrane cluster risk of bias version 2 tool [56]. Robins-I assessed seven domains: (1) bias due to confounding, (2) bias in the selection of study participants, (3) bias in the classification of interventions, (4) bias due to deviations from intended interventions, (5) bias due to missing data, (6) bias in the measurement of outcomes, and (7) bias in the selection of the reported results. Each study was classified as low risk, moderate risk, or high risk for each category. The visualization of risk of the bias plots was generated using Risk-of-bias VISualization (robvis), available online.

### Assessment of quality of evidence

The evidence quality was assessed using the Grading of Recommendations Assessment, Development and Evaluation (GRADE) profiler Guideline Development Tool [57] via an online platform. The overall evidence quality was categorized as “high,” “moderate,” “low,” or “very low”. Any disagreements between the reviewers (CF and WTC) were settled through discussion and consensus, and a third reviewer (SKKL) was consulted when necessary.

### Data analysis

The primary outcomes were mental health literacy, resilience, self-efficacy, and positive thinking. The secondary outcomes were common psychological symptoms such as depression and anxiety and related behavioral problems. The intervention effects were considered for the short-term (< 1-month post-intervention), medium-term (1–6 months post-intervention), and long-term (≥ 6 months post-intervention) follow-up periods when possible.

Although a meta-analysis can be conducted using two studies [58], data from three or more studies reporting the same outcomes were combined for meta-analysis in this review. In studies with multiple arms, the most relevant intervention group was included; if multiple groups were relevant, they were combined following the Cochrane Handbook for Systematic Reviews and Meta-Analyses [56]. Studies that could not be pooled because of significant heterogeneity or insufficient data were analyzed using narrative synthesis.

We estimated the effect sizes as the standardized mean difference (SMD) between the intervention and comparison groups regarding the change in mean values from the baseline to the endline after standardization by their pooled standard deviations (SDs), along with the 95% confidence intervals (CIs). Effect sizes were calculated based on the means and SDs. The formula used was *d* = *(M1*–*M2)/SD*_*pooled*_, where *M1* and *M2* are the means of the intervention and control groups, respectively [59]. When studies did not provide these data, we contacted the authors directly. SMDs of 0.2, 0.5, and 0.8 are considered small, medium, and large effect sizes, respectively [59]. When SDs were not provided and could not be obtained from the author, they were estimated using alternative statistics, such as P-values or t-values. We pooled the effect estimates using a random effects model because of the variability between studies [60]. Heterogeneity was assessed using the *I*^*2*^ statistic and categorized as low (*I*^*2*^ < 40%), moderate (40% ≤ *I*^*2*^ ≤ 75%), or high (*I*^*2*^ > 75%) [61]. The c-RCTs were adjusted for clustering effects using the design effect formula: 1 + (M − 1) × ICC [56]. If the ICC was not reported, an ICC of 0.05 was chosen based on prior studies [31]. The effective sample size calculation of the c-RCTs is shown in SI 2. The sample size was then adjusted accordingly. Leave-one-out sensitivity analysis was performed by removing one study at a time to examine the robustness of the pooled effect. When there was significant heterogeneity in the study, subgroup analyses were conducted based on the type of RCT, age group, and different theoretical foundations. All the analyses were conducted using Review Manager 5.4.

## Results

**Fig. 1:**
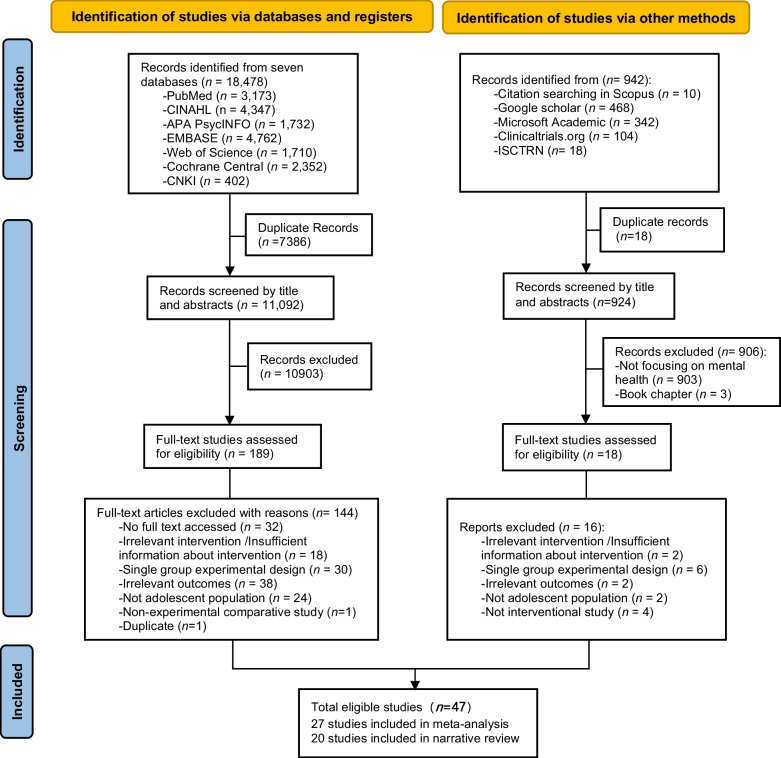
Study Selection. Meta-analyses results of capacity-building interventions on mental health literacy

The initial search identified 18,478 records. After removing 7,386 duplicates, 11,092 articles were screened based on their titles and abstracts. Of these, 10,903 articles were excluded for not meeting the eligibility criteria, leaving 189 articles for full-text assessment. During the full-text screening, 144 articles were further excluded for the following reasons: lack of full-text access (n = 32), irrelevant intervention or insufficient intervention details (n = 18), one-group study design (n = 30), not having the required study outcomes (n = 38), not focusing on adolescents (n = 24), non-experimental study design (n = 1), and duplication (n = 1). This process resulted in 45 articles being chosen for the final review. Moreover, citation screening of eligible studies and searches through electronic engines and trial registries identified two eligible studies; thus, 47 articles [2, 62–107] were ultimately included in the analysis. The study search and selection process according to the PRISMA framework is summarized in Fig. 1.

### Study characteristics

Of the 47 included studies, 17 [2, 63–65, 69, 73, 79, 82, 84, 86, 90, 92, 95, 97, 100, 101, 103] were c-RCTs, 12 [67, 68, 75, 77, 80, 83, 85, 87, 93, 94, 99, 107] were RCTs, 15 [62, 66, 70–72, 74, 76, 78, 81, 89, 91, 96, 98, 102, 104] used an quasi-experimental design, which refers to non-randomized pre–post-tests with a control group [108], two [88, 106] used quasi-c-RCTs, which refers to the absence of randomization, and one [105] used a Solomon four-group design.

The included studies were conducted across 26 countries. Six studies were conducted in Australia [70, 79, 84, 91, 92, 104], five in the United States [69, 73, 87, 93, 96], four in China [83, 85, 101, 107], four in Turkey [62, 67, 77, 94], two in Korea [75, 89], two in Portugal [64, 88], two in the United Kingdom [81, 97], two in Romania [78, 102], and two in the Netherlands [100, 103]. One of each of the other 18 studies [2, 63, 65, 66, 68, 71, 72, 74, 76, 80, 82, 86, 90, 95, 98, 99, 105, 106] were conducted in Greece, Lithuania, India, Indonesia, Ethiopia, Japan, Malaysia, New Zealand, Norway, Tanzania, Spain, Hong Kong, South Africa, Germany, Iran, Lebanon, Ireland, and Zambia.

Most of the studies(n = 40) included both genders, while three targeted only females [2, 82, 89], three targeted only males [75, 84, 104], and one did not report a gender distribution [99]. Of the 47 studies, 84.4% (n = 38) were conducted in school settings; the other studies were carried out in sport clubs [84, 91, 104], community or health service centers [66, 70, 80, 96, 107], or youth shelters for runaways [89]. Notably, 91.5% (n = 43) of the studies focused on resilience and mental health literacy [2, 62–65, 67–85, 87–92, 94–99, 101, 102, 104–107]. The other studies focused on depression, anxiety, self-efficacy, and/or positive thinking [66, 86, 93, 100, 103]. Twelve studies [2, 68, 72, 77, 83, 84, 89, 91, 97, 99, 102, 105] reported no participant dropouts, and only two [66, 88] reported dropout rates higher than 20%.

Nine studies adopted cognitive behavioral theory, either exclusively [75, 77, 85, 86, 93, 94] or in combination with other theories such as social emotional learning practice [63], the activating–belief–consequence model [100], or the extended evolutionary meta-model [90]. Seven studies adopted a resilience framework, either on its own [62, 70, 96, 99, 105] or integrated with other theories such as self-determination theory [87] or social-emotional learning [73]. Other frameworks used in the included studies were positive psychology [82, 95, 101], the community-based participatory framework [104], social learning theory [104], mindfulness-based approaches [69, 102], the mental health literacy framework/model [71, 88, 98], the “Achieving Success Everyday” model [83], positive youth development [72], the integrated behavior change model [84], an inquiry-style psychological counseling model [107], experiential learning theory [68], positive development theory [66], and the three principles of mind, consciousness, and thought [81]. Fifteen studies did not report any theoretical framework or model [2, 64, 65, 67, 74, 76, 78–80, 89, 91, 92, 97, 103, 106]. The study characteristics are summarized in Table 1.

**Table 1:** Study characteristics

Study, country	Design	Setting	Participants'Characteristics	Overall Attrition	Theory/framework
Ataman 2023 (Turkey)	Quasi-experimental design	One public middle school	48 Syrian refugee students (age 10–13, mean 11.2) Female = 50%	12.5% (n = 6: 4 in the intervention group; 2 in the control group)	Hope theory in Resilience framework
Berger 2018 (Tanzania)	Two-arm cluster RCT	One public primary school	183 primary school students (age 11–14) Female = 50.8%	12.00% (n = 24)	Cognitive Behaviour Theory & Social-emotional learning practice
Campos 2018 (Portugal)	Two-arm cluster RCT	Eight public schools	543 primary school students (age 12–14) Female = 43.2%	7.7% (n = 42: 20 in the intervention group; 21 in the control group)	N/A
Casanas 2022 (Spain)	Four-arm cluster RCT	18 public and private schools	1,032 secondary school students (age 13–16, age mean 14.02 ± 0.58) Female = 49.6%	12.2% (n = 126)	N/A
Cerit2021 (Turkey)	Two-arm RCT	One public high school	80 high school students (age 14–17, age mean: 14.9 ± 0.8) Female = 49.1%	12.5% (n = 10: 4 in the intervention group; 6 in the control group)	N/A
Cepukien 2018 (Lithuania)	Quasi-experimental design	Three foster care homes	84 adolescents (age 13–15, age mean: 14.2 ± 0.85) Female = 55.2%	30.0% (n = 26: 3 in the intervention group; 23 in the control group)	Positive developmental theory
Chung 2021 (Hong Kong)	Two-arm RCT	One Integrated Children and Youth Services Centre	228 secondary students living in public housing estates (age 12–16, mean 13.0 ± 0.8), Female = 45.2%	0	Kolb's experiential learning theory
deVilliers201 2(South Africa)	Solomon four group design	Four middle schools in suburbs	161 students (age 11–12) Female = 42.7%	0	Resilience theory
Felver2019 (United States)	Two-arm cluster RCT	One public high school	27 high school students (age range/mean not reported) Female = 66.7%	18.5%(n = 5: all in the intervention group)	N/A
Furness2017 (New Zealand)	Quasi-experimental design	Five secondary schools	80 students with low self-reported low self-efficacy scores (aged 13–15) Female = 58.8%	0	Positive developmental theory Experiential learning theory
Fraser2008 (Australia)	Quasi-experimental design	One community health service centre	44 adolescents who have a parent with a mental illness (age 12–17) Female = 61.4%	9.0% (n = 4: 2 in the intervention group; 2 in the control group)	Resilience framework
Fretian 2023(Germany)	Quasi-experimental design	Three secondary schools and one vocational school	185 secondary and vocational school students (age 14–17) Female = 58.4%	14.3% (n = 31: 17 in the intervention group; 14 in the control group)	Mental health literacy framework
Green 2022 (United States)	Two-arm cluster RCT	Four urban public high schools	372 public high school students (age mean 15.7) Female = 51.9%	6.1%(n = 24: 16 in the intervention group; 8 in the control group)	Resilience and Social Emotional Learning
Hassen 2022 (Ethiopia)	Quasi-experimental design	Public and private high chools	153 adolescents from high schools (age 15–19) Female = 43.8%	10%(n = 18: 7 in the intervention group; 11 in the control group)	N/A
Hyun 2010 (South Korea)	Two-arm RCT	One middle school	34 middle school students whose parent with alcoholic problems (age 12–13) Female = 0%	17% (n = 6: 2 in the intervention group; 4 in the control group)	Resilience and Cognitive Behaviour Theory
Ibrahim 2020 (Malaysia)	Quasi-experimental design	NGO- managed boarding school	101 students from low socioeconomic status (age 13–17, mean 14.61 ± 1.39), Female = 60.4%	12.8% (n = 13: 2 in the intervention group; 11 in the control group)	N/A
Imet 2023 (Turkey)	Two-arm RCT	High school	28 high school students (age range/mean not reported) Female = 50%	0	Cognitive Behaviour Theory
Ionescu 2023 (Romania)	Quasi-experimental design	Three high schools	490 students (age mean 15.56 ± 0.76) Female = 43.9%	35%	N/A
Johnstone 2020(Australia)	Three-arm pilot cluster RCT	Five public primary schools	295 primary school students (age 8–13, 82.9% of which age 10–13, mean 11.04 ± 1.40) Female = 52.5%	15.8% (post-intervention) 42.82% (follow-up)	N/A
Kallinta 2021 (Greece)	Two-arm RCT	Community health service centre	50 adolescents from local community (age 11–17) Female = 70%	2% (n = 1: all in the intervention group)	N/A
Kelley 2021 (UnitedKingdom)	Quasi-experimental design	Six secondary schools	181 secondary students (age 11–15) Female = 6.6%	11.7%(n = 24: all in the intervention group)	Three Principles
Leventhal 2015 (India)	Two-arm cluster RCT	57 government schools in rural areas	2508 rural adolescents living in high-poverty communities (age mean 12.99 ± 1.17) Female = 100%	4.8% (n = 121: 71 in the intervention group; 50 in the control group)	Positive Psychology, Emotional Intelligence, Restorative practice
Liu 2021 (China)	Two-arm RCT	One high school	44 high school students (age 12–18, mean 16 ± 0.7) Female = 59.1%	12.0% (n = 6: 3 in the intervention group; 3 in the control group)	Cognitive Behaviour Theory
Li 2021(China)	Two-arm RCT	One boarding high school	76 high school students from disadvantaged economic status community (age 12–16) Female = 60.5%	0	Achieving Success Everyday (ASE) model
Liddle 2021 (Australia)	Two-arm cluster RCT	One community football club	102 adolescents from community sport club (age 12–18, mean 14.30 ± 1.75) Female = 0%	0	Integrated Behaviour Change model
MaalouFadi 2020 (Lebanon)	Two-arm cluster RCT	10 public and private schools	277 adolescents (age 11–13, mean 12 ± 0.5) Female = 52.7%	19.6% (n = 52) 44 in the intervention group; 11 in the control group)	N/A
Morgado 2021 (Portugal)	Quasi-cluster RCT	One public school	38 secondary school students (age mean 14.50 ± 0.89) Female = 63.2%	29.6% (n = 16: 8 in the intervention group; 8 in the control group)	Health literacy, Mental health literacy
Moran2023 (United states)	Two-arm RCT	One public school	261 adolescents (age 9–13, mean 11.6 ± 0.6) Female = 43.3%	11.0% (n = 31: 13 in the intervention group; 18 in the control group)	Self-determination theory Model of youth resilience
Noh 2018 (South Korea)	Quasi-experimental design	Five youth shelters	32 runaway adolescents lived in youth shelter (age 12–21, mean 16.69 ± 2.56) Female = 100%	0	N/A
O'Connor 2022 (Ireland)	Two-arm cluster RCT	29 elementary schools	604 adolescents from elementary schools (age 10–13, mean 11.07 ± 0.67) Female = 60.0%	14.73% (n = 89, at post-intervention) 32.78% (n = 198, at follow-up)	Process-based Cognitive Behaviour Framework by extended evolutionary meta-model (EEMM)
Patafino 2021 (Australia)	Quasi-experimental design	12 community sporting clubs	330 adolescents from football and netball sports club (age 12–15, mean 13.73 ± 0.79) Female = 41.8%	0	N/A
Perry 2014 (Australia)	Two-arm cluster RCT	10 catholic and independent secondary schools	380 secondary school students (age 13–16, age mean 14.75), Female = 50.0%	15.0% (n = 58, at post-intervention) 45% (n = 172, at follow-up)	N/A
Robinson 2015 (United States)	Two-arm RCT	Four public high school	82 adolescents from low-income African-American communities (age mean 12.92 ± 0.80), Female = 53.7%	27%	Cognitive Behaviour Theory
Sahin 2021 (Turkey)	Two-arm RCT	Public high school	29 high school students (age range/mean not reported) Female = 52%	3.0%	Cognitive Behaviour Theory
Seale 2022 (Zambia)	Two-arm cluster RCT	Private, public and community schools	643 adolescents from general population (age 10–13, mean 11.39 ± 0.95) Female = 53.3%	4.9% (at post-intervention) 19% (at delayed start post-intervention)	Positive psychology and Spirituality
Shelton2006 (United States)	Quasi-experimental design	Local community centre	56 adolescents who were at risk of delinquency (age 10–14, mean not reported) Female = 55.4%	Not reported	Ecological-resiliency model
Simkiss 2023 (Wales)	Two-arm cluster RCT	Public secondary schools	1926 secondary school students (age 13–14, mean not reported), Female = 55.3%	0	N/A
Skre 2013 (Norway)	Quasi-experimental design	Four secondary schools	1070 secondary school students (age 13–15, age mean 14.06), Female = 46.1%	2.7% (n = 29)	Jorm's mental health literacy framework, Antonovsky’s theory of salutogenesis
Suranata 2020 (Indonesia)	Three-arm RCT	Junior high school	90 high school students (age mean 12) N/A	0	Bernard's the framework of resilience theory
Tak 2014 (Netherlands)	Two-arm cluster RCT	Secondary schools	1341 secondary school students (age range not reported, mean 14.0 ± 0.5) Female = 47.3%	3.5% (at post-intervention) 15.5%(at 2-year follow-up)	Cognitive Theory and Activating-Belief-Consequence Model
Tang 2022 (China)	Two-arm cluster RCT	Primary and secondary schools	1613 primary and secondary school students (age mean 12.49 ± 1.67) Females = 49.4%	3.5%	Positive Psychology, Social Cognitive Theory
Tripa 2021 (Romania)	Quasi-experimental design	Rural secondary schools	62 secondary school students who are left-behind (age 12–15, mean 12.71 ± 0.868), Female = 53.2%	0	N/A
Tuijnman2022 (Netherlands)	Two-arm cluster RCT	Secondary schools	185 secondary school students (age 12–15, mean 13.43 ± 0.67) Female = 45.4%	3.1% (n = 6)	N/A
Vella 2021 (Australia)	Quasi-experimental design	Community sport clubs	816 adolescents from community-based non-elite organised sports clubs (age 12–17, mean 13.43 ± 0.67) Female = 0%	70%	Community-Based Participatory Research (CBPR) framework, Socio-ecological approach
Yamaguchi 2020 (Japan)	Quasi-cluster RCT	Public high school	899 high school students (age 15–16, mean not reported) Female = 59.3%	8.2%	N/A
Zare 2021 (Iran)	Two-arm cRCT	Public high school	220 female students (aged13–15) Female = 100%	0	Social Learning Theory
Zhang 2021 (China)	Two-arm RCT	Three local communities	153 adolescents with anxiety symptoms (age 12–18) Female = 47.7%	4.4%	Inquiry style psychological counselling model

*RCT* Randomized controlled trial, *N/A* Not reported.

### Intervention characteristics

Most of the studies adopted multi-component interventions to enhance adolescents’ mental health capacities. These included (1) psychoeducation to improve mental health awareness and knowledge, (2) skill-building activities such as emotional regulation, coping, problem-solving, peer groups, and mindfulness, and (3) fostering social connections through community engagement, supportive peer interactions, and group sports activities. Five studies [63, 74, 79, 85, 92] had an active group, such as a social studies intervention covering civics or the general values of the country. Forty-two studies [2, 62, 64–73, 75–78, 80–84, 86–91, 93–107] compared their control group with a waitlist or no intervention group. Two studies using three-arm trials [79, 99] evaluated different interventions against a control group. In one trial [79], the participants were divided into an emotional regulation group, a behavior activation group, and a control group following Australia’s national health and physical education curriculum. We chose the emotional regulation group for the meta-analysis because of its alignment with our objective of enhancing individual skills in adolescents. The second trial [99] compared an internet-based cognitive behavioral therapy group to a waitlist control. Additionally, one trial [65] compared three intervention groups with a waitlist group. A waitlist group [109] is a control group that did not receive the intervention or treatment immediately but was placed on a waiting list to receive it at a later time.

The durations of the interventions ranged from a single session to 14 months, with the total number of sessions varying from 1 to 48. Each session lasted between 45 and 100 min. However, 13 studies [68, 71, 74, 76, 78, 80, 92, 94, 97–99, 104, 106] did not clearly report the details of the duration, number of sessions, or intensity/time intervals of their interventions. All of the studies delivered interventions through group-based face-to-face sessions except for two studies that used an individual approach (i.e., one with an online platform [74] and another with a digital game [103]). The delivery methods varied considerably, including PowerPoint presentations, videos, and interactive activities like exercises, games, role-playing, discussions, and home assignments. One study featured individuals with mental illness sharing their rehabilitation and recovery stories [84]. Nearly half of the interventions (n = 20) [62, 64, 65, 67, 68, 70, 73, 75, 77, 79, 83, 86–91, 94, 100, 105] were led by mental health professionals, including qualified psychological counsellors, clinical psychologists, mental health nurses, and social workers. Nine [63, 69, 71, 78, 81, 92, 97, 98, 106] were led by trained school teachers. Four [66, 76, 80, 104] were conducted by researchers but did not present details about their qualifications. The others (n = 12) [2, 72, 82, 84, 85, 93, 95, 99, 101–103, 107] were mainly facilitated by volunteers, community facilitators, educators, or counselors. However, one intervention [74] had no group leader or facilitator, and another [96] did not specify this information.

In addition to baseline measurements, all of the studies performed post-intervention assessments. Twenty-six [63–65, 67, 68, 70, 72, 76–79, 83, 85, 88–90, 92, 94, 98–101, 103, 105–107] of the 47 studies conducted follow-up assessments between one month and two years after. The most common primary outcomes used were mental health literacy (*n* = 16) and resilience (*n* = 27). Several studies assessed two primary outcomes, including self-efficacy and resilience [87], self-efficacy and positive thinking [93], and mental health literacy and resilience [104]. Nine studies (56%) [65, 70, 71, 76, 81, 84, 88, 90, 92] used multiple scales to assess different components of mental health literacy, and six studies used overall scales commonly used in the field, including the Mental Health Literacy Questionnaire [64, 74], Mental Health Literacy Scale [84, 91], Knowledge and Attitudes to Mental Health Scales [97], and the Romanian version of the Mental Health Knowledge and Attitudes Scale [78]. The level of resilience was assessed using the Connor–Davidson Resilience Scale [82, 90, 95, 104], Resilience Scale –14 items [68, 83, 85, 101], and Child and Youth Resilience Measure [62, 79, 80, 87]. Self-efficacy was measured with the Self-Efficacy Questionnaire [72, 87, 99], Schwarzer’s General Self-Efficacy Scale [82], Coping Self-Efficacy Scale [83], Help-Seeking Self-Efficacy Scale [71], and Self-Efficacy Scale [66]. Positive thinking was assessed with the Positive Automatic Thoughts Questionnaire [93]. Table 2 summarizes the main characteristics of interventions.

**Table 2:** Intervention characteristics

Study	Intervention (Duration, frequency, content)	Comparison(s)	Format of delivery	Outcomes (instrument, time points for assessment),
Ataman 2023	**Hope-based intervention** - **5 weeks** (weekly 90-min session, 2 sessions during 1 st week) - Session contents: Introduction of Hope theory; Encouraging students to set goals and developing capacity to conceptualize goals, encouraging creation of specific strategies and pathways to achieve these goals, discussions and peer experience sharing	Usual school; no intervention	Group-based, face-to-face Graduate psychology student	Resilience (CYRM-12) At baseline, immediate post-intervention
Berger 2018	**Culturally-adapted ERSAE-Stress-Prosocial intervention(ESPS)** - **8 weeks** (weekly 90-min session) - Session contents: a warm-up exercise, experimential work, psycho-educational knowledge, a contemplative practice, a learned skill and homework assignments	Social studies (covered the history, civics) - 1 sessions, 120 min/session	Group-based, face-to-face School teachers	Anxiety (SCAS) At baseline, 1-week post-intervention, 8-month follow-up
Campos 2018	**"Finding Space"interactive psychoeducation sessions** - **2 weeks** (weekly 90-min session) - Session contents: 1 st session: exploration of mental health knowledge and beliefs; identification of risk factors, symptoms and signs of mental health disorders; promotion of non-stigmatized behaviours 2nd session: raising awareness of mental health problems; identifying formal and informal help-seeking options; promoting first-aid and self-help skills through group dynamics, music, and videos and take-home assignments	Usual school; no intervention	Group-based, face-to-face Trained psychologist	Mental health literacy (MHLq) At baseline, 1-week post-intervention, 6-month follow-up
Casanas 2022	**Intervention 1: Mental Health Literacy + Stigma Reduction programme** -**7 weeks (**weekly 60-min session**)** (concepts of mental health and mental health disorders, social skills, healthy behaviours of mental health, stigma) **Intervention 2: Mental Health Literacy programme** —**6 weeks (**weekly 60-min session**)** (concepts of mental health and mental health disorders, social skills, healthy behaviours of mental health) **Intervention 3: Sensitive programme** - **1 week(**weekly 60-min session**)** (broad topic on mental health and emotional regulation)	Waitlist group	Group-based, face-to-face Mental health nurses	Mental health knowledge(EMHL Test), At baseline, 2-week post-intervention, 6-month follow-up, 12-month follow-up,
Cerit 2021	**Social skill training** - **7 weeks (**weekly 40-min session) - Session contents: Training of self-awareness, self-efficacy, coping, positive way of thinking, problem solving skills, emotion management, empathy exercises, discussion and take-home assignment	Usual school; no intervention	Group-based, face-to-face Mental health nurses	Resilience (Gürgan Resilience scale), At baseline, post-intervention, 1 month follow-up
Cepukien 2018	**Solution-focused self-efficacy enhancement training** - **3 weeks** (weekly 90-min session) - Session content: Narrative of the train journey	Usual school; no intervention	Group-based, face-to-face Researchers (authors)	Self-efficacy (Self-efficacy scale) At baseline, post-intervention
Study	Intervention (Duration, frequency, content)	Comparison(s)	Format of delivery	Outcomes (instrument, time points for assessment),
Chung 2021	**Adventure-based training** - 2‐day/1‐night summer camp - activities various games + health education talks	Usual health education	Group-based, face-to-face Certified professional adventure‐based educators and nurses	Resilience (RS-14) Depressive symptoms (CES‐DC) At baseline, 3-month and 6-month post-intervention
deVilliers2012	**Intervention 1(resiliency intervention) = Intervention 2** - **15 days** (daily 90-min session) - Session contents: resiliency intervention focused on intrapersonal competencies (awareness and regulation of behavioural, cognitive and emotional adjustments) and interpersonal skills	Usual school; no intervention	Group-based, face-to-face Psychologists	Resilience (RSCA) At baseline (Intervention1 and Comparison1), post-intervention(all four groups), 3-month follow-up (all four groups)
Felver 2019	**L2B mindfulness skill training** - **7 weeks**(weekly 48-min session) - Session contents: Body awareness, self-reflections, emotion regulation, reducing self-judgment, building positivity through mindfulness, emotional balance with meditation	Usual health education	Group-based, face-to-face School teachers	Resilience (SEARS-SF) At baseline, 1-week post-intervention
Furness 2017	Project K program −**14 months** program -Session content: 3-week wilderness adventure program with outdoor activities; 10-day community challenges and 12-month mentoring partnership	Waitlist group	Group-based, face-to-face Not reported	Resilience (RS-25) Self-efficacy (Project K Self-Efficacy Questionnaire) At baseline, 14-months post-intervention
Fraser 2008	**Koping Adolescent Group Program (KAP)** - **3 weeks** (weekly 120-min session) —Session contents: Psychoeducation of mental health; experience sharing of having a parent with a mental illness; coping skills training through group discussion, quizzes, and other activities (e.g. creative art and craft, videos, games);	Waitlist group	Group-based, face-to-face Mental health clinicians	Mental health literacy (researcher-modified-Knowledge of mental illness dimension and awareness of mental illness dimension), At pre-, post-intervention, 8-week follow-up
Fretian 2023	**Mental health curriculum (MHC)** - 1-day psychoeducation + presentation of personal experience of mental illness - Session contents: (1) destigmatizing mental illnesses, (2) understanding mental health and mental illness, (3) symptoms, treatment, risk factors regarding specific mental illnesses, (4) lived experiences of mental illness, (5) help-seeking and support, and (6) positive mental health	No intervention (next term of 10 th grade students)	Group-based, face-to-face School teachers and school counsellors	Mental health knowledge (MHKS), Help-seeking efficacy (HSES) At baseline, post-intervention
Green 2022	**SPARK teen mentoring intervention** -**13 days** (daily 60-min session) - Session contents: understanding the mind, embracing positive thoughts, strengthening goal-setting and decision-making skills, reasoning, mood management, self-esteem through group discussions, role plays, and video watching	Usual school curriculum	Group-based, face-to-face Trained facilitators with classroom experience	Resilience (RSCA) At baseline, and immediate post-intervention
Hassen 2022	**Mental health literacy curriculum** —**6 weeks** (8 sessions) - Session contents: materials of seeking information, understanding common disorders and their risks, self-help methods, and available mental health support	Information regarding “secrets of successful students” - 8 sessions, 6 weeks	Individual-based, social media platforms No facilitator	Mental health literacy (MHLq) At baseline, mid-intervention, 1-week post-intervention
Hyun 2010	**Cognitive behavioural therapy** - **10 weeks** (weekly 50-min session) - Session contents: cognitive restructuring (e.g.increasing self-consciousness and identifying dysfunctional coping) and behavioural activation (e.g.developing healthy coping strategies	Usual school; no intervention	Group-based, face-to-face Trained CBT nurses	Resilience (KARS), Depression (RADS) At baseline and post-intervention
Ibrahim 2020	**Depression literacy intervention** - Single session - Session content: lecture, group activities, mental awareness activities, and a short video on depression	Usual school; no intervention	Group-based, face-to-face Researchers	Mental Help Seeking Attitude (MHSAS), Depression-specific mental health literacy (D-Lit), At baseline,post-intervention (only intervention group),3-month follow-up
Imet 2023	**Cognitive behavioural group counselling** -**12 weeks** (weekly 65-min session) -Session contents: Recognizing emotions and developing emotion regulation skill	Usual school; no intervention	Group-based, face-to-face Trained psychological counsellor	Resilience (APRS), At baseline,post-intervention, and 2-month follow-up
Ionescu-Corbu 2023	The Guide psychoeducation - **6–8 weeks** (6 modules) - Session contents: 2 modules(stigma reduction), 1 module (brain function and mental health), 1 module (information about mental illnesses), 2 modules (how to maintain a good mental health and how, when and where to seek help)	Usual school; no intervention	Group-based, face-to-face School teachers and school counsellors	Mental health literacy (MHKAS) At baseline, post-intervention, 2-month follow-up, 1-year follow-up
Johnstone2020	**Intervention 1: Emotion regulation (ER) program** - **8 weeks** (weekly 50-min session) (building emotional regulation skills and develop strategies to manage negative emotions) Intervention 2: Behaviour activation (BA) program - 8 weeks (weekly 50-min session) (developing adaptive behaviours)	Australian national health and physical curriculum	Group-based, face-to-face Psychologists	Resilience (CYRM-12), Depression and anxiety (CDAS), At baseline, post-intervention, and at 6‐month follow-up
Kallinta 2021	**Stress management program** —**8 weeks** (8 modules, twice a day) - Session contents: diaphragmatic breathing, progressive muscle relaxation and guided imagery with presentation and discussion	Usual school: no intervention	Group- and individual-based, face-to-face Researchers(authors)	Resilience (CYRM), Anxiety (SCAS), At baseline, post-intervention
Kelley 2021	**Innate Health Education and Resilience Training (iHEART)** - **10 weeks** (weekly 50-min session) —Session contents: how mind works, building youth's skills on how to deal real-life issues, and discussion	Usual school; no intervention	Group-based, face-to-face School teachers	Resilience (I-ORQ) At baseline, post-intervention
Study	Intervention (Duration, frequency, content)	Comparison(s)	Format of delivery	Outcomes (instrument, time points for assessment),
Leventhal2015	**Combined curriculum (RC + HC) + Girls First Resilience Curriculum (RC)** - **23 weeks** (weekly 60-min session) - Session contents: 1–3 Identifying the character strengths and plan the goals 4–19 Building on character strength to develop coping skills and emotional intelligence skills to manage difficult situations and emotions 20–23 Collaborate in group to exercise the skills, discussion, and experience sharing	Usual school; one additional after school hour	Group-based, face-to-face Trained female community facilitators	Emotional resilience (CDRS-10), Self-efficacy (GSES), Depression (PHQ-9), Anxiety (GAD-7) At baseline, post-intervention
Liu 2021	**Group counselling resilience intervention** —**10 weeks** (weekly 120-min session) —Session contents: 1 st: Program introduction 2nd—3rd: Techniques for emotional regulation 4 th—5 th: Enhancing interpersonal and communication skills 6 th—7 th: Building family support 8 th—9 th: Developing positive cognition and thinking 10 th: Cultivating appreciation and gratitude	Life planning course	Group-based, face-to-face Qualified psychological counsellors	Resilience (RS-C), At baseline, post-intervention, 2-month follow-up
Li 2021	**ASE model-based resilience intervention** - **8 weeks** (weekly 60-min to 90-min session) - Session contents: cognitive and skill-based components, guiding adolescents to reinterpret challenges, enhanced their skills and encouraged the adoption of positive coping strategies as alternatives to negative behaviours	Usual school; no intervention	Group-based, face-to-face Qualified psychological counsellors	Resilience (RS-C), At baseline, post-intervention, 3-month follow-up
Liddle 2021	**Help Out a Mate (HOAM) intervention** - Single workshop session(45 min) - Session contents: psychoeducation of mental health and mental illness problems, building confidence in helping somebody with a mental health disorder, and positive attitudes toward people with mental disorders through group discussion, role-play interactive activity	Waitlist group	Group- & sport-based, face-to-face Trained male volunteers with lived experience of mental illness	Mental Health Literacy (MHLS), At baseline, 2-week post-intervention, 4-week post-intervention
MaalouFadi 2020	**Culturally adapted My FRIENDS Youth intervention** - **10 weeks** (weekly 30-min to 45-min session) - Session contents: Education related to resilience and mental health concepts (e.g. understanding feelings, practicing empathy, learning to relax, challenging one’s thoughts, problem solving, and making and keeping friends)	Waitlist group	Group-based, face-to-face Mental health professionals(authors)	Depressive symptoms (MFQ) Anxiety (SCAR) At baseline, post-intervention
Morgado 2021	**ProLiSMental psychoeducation** - **4 weeks** (weekly 90-min session) OR **8 weeks**(weekly 45-min session) - Sessions contents: recognize the mental health and emotions, mental health promotion, prevention and self-help strategies, mental health first aid actions, and seeking informal and formal help, from knowledge on mental health and anxiety prevention, and management to action in everyday life skills	Waitlist group Usual school; no intervention	Group-based, face-to-face Mental health nurses	Mental health literacy (QuALiSMental) At baseline, post-intervention, and 1-month follow-up for intervention group At 1 month before, baseline, and post-intervention for control group (no follow-up)
Study	Intervention (Duration, frequency, content)	Comparison(s)	Format of delivery	Outcomes (instrument, time points for assessment),
Moran 2023	**Healthy Kids program** -**6 weeks** (weekly 30-min session) -Session contents: introduction of the resilience topic; 2-min mindfulness experiences; discussion of strategies to improve resilience	Usual school; no intervention	Individual-based, face-to-face Certified health coaches	Resilience (CYRM-R) Self-efficacy (SEQ-C) At baseline, post-intervention
Noh 2018	**Resilience enhancement programme** - **4 weeks** (weekly 180-min session) - Session contents: Improving self-esteem and self-regulations; promoting cognitive restructuring for the regulation of negative emotions through positive and resilient thinking; enhancing positive connectedness and relational skills; developing problem-solving and goal-setting skills and reflection of the sessions	Usual care; no intervention	Group-based, face-to-face Mental health nurses	Resilience (YKRQ-27), Depression (BDI-II), Anxiety (BAI), At baseline, post-intervention, 1-month follow-up
O'Connor2022	**A Lust for Life, process-based CBT culturally-sensitive intervention** - **6 weeks** (weekly 40-min session) - Session contents: Discussion and psychoeducation of mental wellbeing; mindfulness practice; cognitive reappraisal; education of interpersonal skills; discussion of cognitive bias	Waitlist group; received the same intervention six weeks after the intervention group finished theirs	Group-based, face-to-face Clinical psychologist with expert training in child and adolescent mental health	Resilience (CDRS-10), Emotional literacy (Emotional Literacy Student Checklist) At baseline, immediate post-intervention, and at 6-week follow-up
Patafino 2021	**Read the Play psychoeducation about mental health** (in particular, mood disorders, anxiety disorders, suicide, substance use, and personality disorders) - Single session (60 min) - Session contents: Education and discussions on mental health and mental illness, addressing stigma toward mental health illness, building help-seeking behaviours and strategies for self-care through interactive games	Waitlist group	Group-based, face-to-face Mental health professionals	Mental health literacy (MHLS), At baseline, immediate post-intervention, and 2–4 week post intervention follow-up
Perry 2014	**Head Strong psychoeducation curriculum** -** 5–8 weeks** (10 h sessions) - Session contents: Education of mood and mental wellbeing; reaching out and help peers; building resilience and exercising mind to help oneself; empower individuals to take actions	National curriculum – self and relationship, movement skill, individual and community health, and physical activity	Group-based, face-to-face School teachers	Mental health literacy (D-Lit), At baseline, post-intervention, and 6-month follow-up
Robinson 2015	**Culturally adapted Adolescent Coping with Stress Course (CWS)** - **15 weeks** (weekly 45-min session) - Session contents: recognizing negative emotions and stress, introducing self-help strategies to restructure cognitive distortions, identifying negative and positive thoughts, practising positive thinking exercises, through discussion, group activities & home exercises	Standard care; **-** one-to-one session focused on stress management strategies	Group-based, face-to-face Social worker and psychology interns supervised by clinical psychologist	Perceived self-efficacy to cope (DCSE), Positive thinking (ATQ-P), Anxiety (STAI-T), At baseline, post-intervention
Sahin 2021	**Cognitive-behavioural group psychoeducation program** - **10 weeks** (10 sessions) - Session contents: identifying the emotions and thoughts, knowing the ability to change, building communication skills, improving self-esteem, improving skills of assertiveness, and coping skills	Usual school; no intervention	Group-based, face-to-face Qualified psychologist with CBT trainings	Resilience (Gürgan's Resiliency Scale), At baseline, post-intervention, 5-month follow-up
Seale 2022	**Culturally adapted Global Resilience Oral Workshops (GROW)** —**24 weeks** (weekly 90-min session) - Session contents: building 24-character strengths from worldwide cultures and religious traditions and delivered through Bible storytelling with interactive activities (e.g. drama, prayer, snacks, creative activities, and games)	Delayed start waitlist; received same intervention after the completion of the intervention group	Group-based, face-to-face, Trained community facilitators	Resilience (CDRS), At baseline, immediate post-intervention, immediate delayed-start post-intervention
Shelton 2006	**The LEAD after school program with creative arts** - **14 weeks **(weekly 200-min session) (two semesters: Fall semester and Spring semester) - Session contents: coping skills and relaxation, exploration of self and self-identity, promoting a sense of family and social connectedness	Usual after-school program; no description of details	Group-based, face-to-face Not reported	Resilience (Polk-20), At baseline, post-intervention
Simkiss 2023	**Guide Cymru psychoeducation** - NA, NA,12 weeks - Session contents: understanding mental health and mental illness, stigma myths and realities, information on specific mental illnesses, experiences of mental illness, help-seeking and finding support, and the importance of positive mental health	Usual teaching; no description of details	Group-based, face-to-face School teachers	Mental health literacy (KAMHS) At baseline, post-intervention (no follow-up data due to Covid-19 restriction)
Skre 2013	**Mental health for everyone programme** —3-day intervention - Session contents: theme of Self-awareness and Identity, Being different, and Loneliness and Fear of the Unknown	Usual school; no intervention	Group-based, face-to-face School teachers	Knowledge about mental health care system (researcher modified questionnaire) At baseline and 2-month follow-up
Suranata 2020	**Intervention 1:** **Cognitive behavioural group counselling session** - **8 weeks** (weekly 55-min session) - Session contents: cognitive technique such as change individual's cognitive distortion, with rational emotive and behavioural techniques such as analysis of self-talk, social skill, problem solving and relaxation training **Intervention 2:** **Internet-based cognitive behavioural counselling session** - **3–5 weeks** (weekly 1 session) - Same session content as the intervention 1	Waitlist group	Group-based, face-to-face and Individual-based, online School counsellors	Resilience (RYDM) At baseline, post-intervention and 5-week follow-up
Tak 2014	**Culturally adapted resiliency school curriculum** - **16 weeks**(weekly 50-min session) + 1 booster session (120 min) - Session contents: Introduction of CBT principles; Skill-building education (e.g. coping skills, decision-making skills, problem-solving skills) and interactive participation and discussion	Usual school; no intervention	Group-based, face-to-face Clinical psychologists	Self-efficacy (SEQ) Depressive symptoms (CDI), Anxiety (RCMAS), At baseline, immediate post-intervention, 6-month follow-up, 1-year follow-up, 18-month follow-up and at 2-year follow-up
Tang 2022	**Peer education intervention** —1 year education with minimum 2 activities/month - Session contents: physical and mental health knowledge, healthy behaviour and lifestyle, and peer education knowledge and skills	Usual school; no intervention	Group-based, face-to-face Peer educators from each class trained by health care specialists	Resilience (RS-C) At baseline and 1-year follow-up
Tripa 2022	**"Resilient left-behind children"SEL and counselling program** - **6 weeks** (weekly 90-min session) - Session contents: self-awareness and awareness of others, building social problem solving and decision-making skills, and practicing mindfulness	Usual school; no intervention	Group-based, face-to-face School counsellor	Resilience (BRS), At baseline and post-intervention
Tuijnman2022	**A game-based program"Moving Stories"** -** 1 week** - Game content: Adolescents engage with Lisa, a character displaying symptoms of depression (though not explicitly labelled as such). Adolescents are asked with assisting Lisa, which they can achieve by executing 5 specific actions daily	Usual school; no intervention	Individual- and group-based, online Volunteer with depression experience	Depression (CDI) At baseline,post-intervention, 3-month,and 6-month follow-up
Vella 2021	**The Ahead of the Game Mental Health Literacy Workshop and Resilience Intervention** **Mental Health Literacy Workshop** - NA, 45 min/session, **NA** - Content: Education of recognizing signs of depression and anxiety, encouraging self-help and help-seeking behaviours, and providing guidance on where to find help **Resilience intervention** - Single face-to-face session(45 min) + 6 internet-based sessions (15 min/session) -Content: Building key psychological skills to cope with adversity, using sport-based examples. Additional details are not provided	Usual sport practice	Group- and individual-based, face-to-face and online Researcher (author) and one volunteer	Depression and Anxiety literacy (DLQ &ALQ), Resilience (CDRS) At baseline, And 1 m follow-up
Yamaguchi 2020	**MHL educational program for adolescents (SMHLP)** (intervention adapted from: Oijo, 2015) - **NA** (2 sessions, 50 min/session)_ - Session contents: education about mental disorders and the importance of mental health, knowledge about symptoms, building help-seeking skills and skills to support others with mental health problems, animated films watching and discussion	Waitlist group	Group-based, face-to-face, School teachers	Knowledge about mental health/illnesses At baseline, immediate post-intervention, 2-month follow-up
Zare 2021	**Mental Health and High School Curriculum Guide** (Intervention based on: Kutcher, 2017) - **6 weeks** (weekly 60-min to 90-min session) - Session contents: understanding mental health and mental illness; stigma of mental illness and information on mental illness, experience of mental illness, seeking help and finding support, importance of positive mental health	Usual school; no intervention	Group-based, face-to-face Researcher (authors)	Mental health literacy (MHLq) At baseline, and 2-week of post-intervention
Zhang 2021	**Psychological counselling + outdoor exercise** - Psychological counselling: **8 weeks** (weekly 60- min session) - Outdoor exercise: **8 weeks**(weekly 100-min session)	Usual school; no intervention	Group-based, face-to-face Counsellor + sport expert	Resilience (HKRA), Depression (SDS), Anxiety (SAS), At baseline, 2-month of post-intervention

*CYRM* Child and Youth Resilience Measure, *SCAS* Spence Anxiety Scale For Children, *MHLq* Mental Health Literacy Questionnaire, *EMHL* EspaiJove Mental health Literacy, *RS* Resilience Scale, *CES‐DC* Chinese version of the Center for Epidemiologic Studies Depression Scale for Children, *RSCA* Resiliency Scale for Children and Adolescents, *SEARS-SF* Social-Emotional Assets and Resilience Scales, *MHKS* Mental Health Knowledge Scale, *HSES* Help-seeking efficacy scale, *KARS* Korean Adolescent Resilience Scale, *RADS* Reynolds Adolescent Depression Scale, *MHSAS* Mental Help Seeking Attitude Scale, *D-Lit* Depression Literacy Scale, *APRS* Adolescent psychological resilience scale, *MHKAS* Mental Health Knowledge and Attitudes Scale, *CDAS* Modified Child Anxiety and Depression Scales, *I-ORQ* Inside-Out Resilience Questionnaire, *CDRS* Connor-Davidson Resilience Scale, *GSES* Schwarzer's General Self-Efficacy Scale, *PHQ-9* Patient Health Questionnaire-9, *GAD-7* General Anxiety Disorder-7, *MHLS* Mental health literacy scale, *MFQ* Mood and Feelings Questionnaire, *SCAR* Scale for Childhood Anxiety and Related Disorders, *QuALiSMental* Questionnaire for Assessment of Mental Health Literacy*, SEQ-C* Self-Efficacy Questionnaire for Children, *YKRQ*Youth Korea Resilience Quotient, *BDI-II* Beck Depression Inventory-II, *BAI* Beck Anxiety Inventory, *DCSE* Depression Coping Self-Efficacy scale, *ATQ-P* Positive Automatic Thoughts Questionnaire, *STAI-T* State-Trait Anxiety Inventory-Trait, *Polk-20* Polk Resilience Patterns Scale-20, *RYDM* Indonesian version of Resilience Youth Development Module, *SEQ* Self-Efficacy Questionnaire, *CDI* Children’s Depression Inventory, *RCMAS* Revised Children’s Manifest Anxiety Scale, *BRS* Brief Resilience Scale, *CDI* Children’s Depression Inventory, *DLQ &ALQ* Depression Literacy Questionnaire & Anxiety Literacy Questionnaire, *HKRA* Healthy Kids Resilience Assessment, *SDS* Self-rating Depression Scale, *SAS* Self-rating Anxiety Scale

### Risk of bias assessment

Twenty-nine studies [2, 63–65, 67–69, 73, 75, 77, 79, 80, 82–87, 90, 92–95, 97, 99–101, 103, 107] were assessed for risk of bias using the RoB 2 tool for RCTs and c-RCTs, and the other 18 studies [62, 66, 70–72, 74, 76, 78, 81, 88, 89, 91, 96, 98, 102, 104–106] were evaluated for risk of bias using the Robins-I tool. The methodological quality of the studies ranged from moderate to high risk of bias. Four studies conducting RCTs [77, 81, 85, 93] had a high risk of bias, with main concerns stemming from randomization and deviations from intended interventions. Only one RCT study [68] detailed its allocation concealment, and all [67, 68, 75, 77, 80, 83, 85, 87, 93, 94, 99, 107] of the RCT studies had “some concerns” in their outcome measurements because of self-reporting. The 17 c-RCT studies [2, 63–65, 69, 73, 79, 82, 84, 86, 90, 92, 95, 97, 100, 101, 103] generally showed low risk of bias in random generation and participant recruitment. However, nine of these studies lacked allocation concealment [2, 64, 69, 73, 79, 82, 95, 101, 103], of which six [2, 63, 65, 95, 97, 101] had concerns regarding deviations from interventions and seven [69, 73, 79, 82, 90, 95, 103] had missing outcome data. The 18 quasi-RCT studies [62, 66, 70–72, 74, 76, 78, 81, 88, 89, 91, 96, 98, 102, 104–106] had concerns about confounding variables and missing outcome data. Ten of them [62, 66, 70, 72, 78, 81, 96, 102, 105, 106] did not report adjustments for confounding factors, and eight did not report how missing data were handled [62, 70, 71, 74, 76, 78, 81, 88, 96, 104]. The risk of bias assessment charts for all of the studies are presented in SI 3, and the risk of bias assessment table is given in SI 4.

Of the 47 included studies, only 27 [2, 62–64, 67–69, 72, 73, 75, 79, 81–87, 89, 90, 92, 95, 99, 101, 102, 105, 107] were pooled in the meta-analysis. The other 20 lacked basic descriptive statistics (means, standard deviations, SE, P-values) [66, 74, 76–78, 80, 88, 91, 93, 94, 96], reported inconsistent participant numbers [97, 104], or used some measurements unsuitable for combination into a composite score [65, 70, 71, 98, 100, 103, 106]. Because we predetermined that only outcomes with three or more studies at the same time could be pooled, the limited number of studies measuring our primary outcomes made it impossible to conduct a meta-analysis. The meta-analysis focused on two primary outcomes: mental health literacy and resilience. Self-efficacy (n = 2) and positive thinking (n = 1) were pooled in the narrative analysis because of a lack of comparable studies and measurement variability.

## Meta-analysis

### Primary outcomes

#### Mental health literacy

**Fig. 2:**
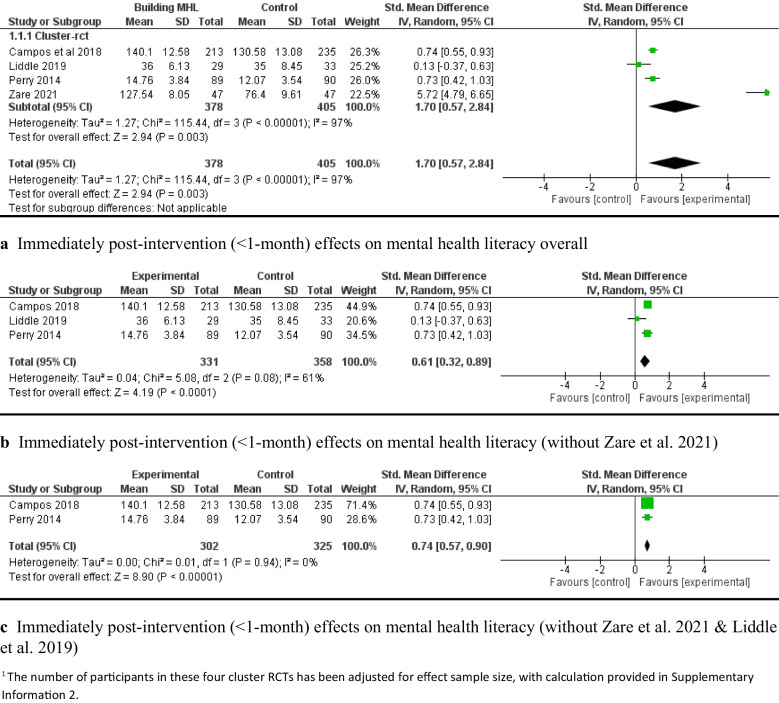
Immediately post-intervention effects of capacity-building interventions on mental health literacy

Regarding the short-term effectiveness of the interventions (< 1-month post-intervention), four c-RCTs [2, 64, 84, 92] assessed mental health literacy outcomes. The random-effects meta-analysis results revealed that capacity-building interventions significantly improved mental health literacy compared with the control groups (e.g., usual care or waitlist) from immediately post-intervention to one month afterward (*SMD* = 1.70, 95% *CI*: [0.57, 2.84], *P* < 0.05; 4 c-RCTs; 783 participants; Fig. 2a). However, there was significant heterogeneity (*I*^*2*^ = 97%). A leave-one-out analysis indicated that the study by Zare et al., focusing solely on female adolescents, contributed most to the high heterogeneity (*z*-value = 12.08, *P* < 0.001). With this study excluded, the pooled effects of the capacity-building intervention on mental health literacy remained significant, with moderate heterogeneity (*SMD* = 0.61, 95% *CI*: [0.32, 0.89], *P* < 0.001, 689 participants, *I*^*2*^ = 61%; Fig. 2b). Similarly, removing the single-gender study of Liddle et al. [58] resulted in a significant pooled effect with low heterogeneity (*SMD* = 0.74, 95% *CI*: [0.57, 0.90], *P* < 0.001, 627 participants, *I*^*2*^ = 0; Fig. 2c).

### Resilience

#### Short-term effectiveness of the interventions (< 1 month post-intervention)

**Fig. 3:**
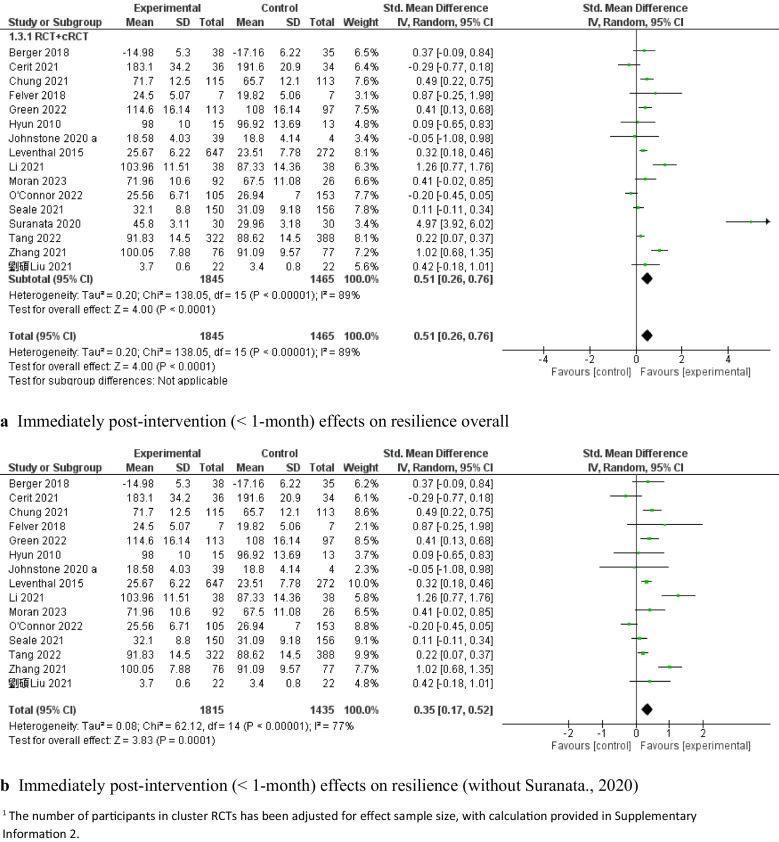
Immediately post-intervention effects of capacity-building interventions on resilience

Sixteen studies [63, 67–69, 73, 75, 79, 82, 83, 85, 87, 90, 95, 99, 101, 107] measured resilience outcomes. The results of a random-effects meta-analysis showed that capacity-building interventions resulted in a statistically significant improvement in resilience compared with the control groups (*SMD* = 0.51, 95% *CI*: [0.26, 0.76], *P* < 0.001, 8 c-RCTs and 8 RCTs, 3,310 adolescents; see Fig. 3a), with high heterogeneity (*I*^*2*^ = 89%). A subgroup analysis was then conducted on the study designs, theoretical frameworks, and types of adolescent populations.

**Fig. 4:**
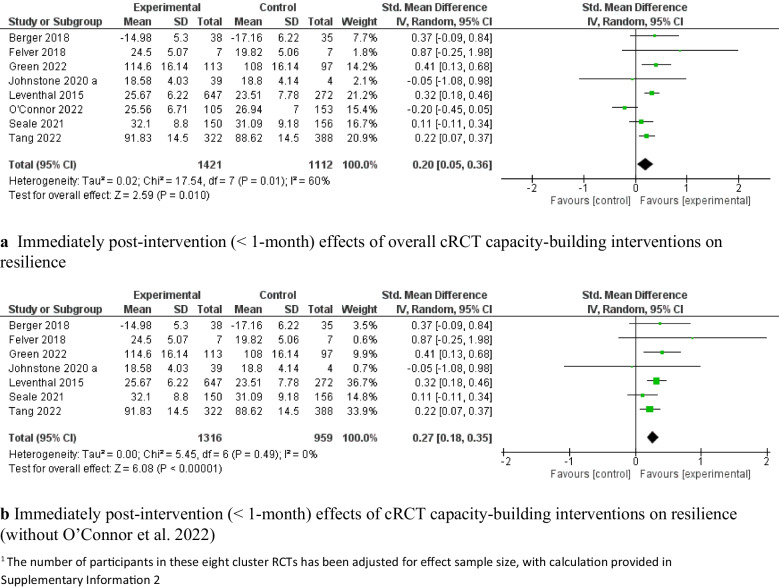
Immediately post-intervention effects (< 1-month) of cRCT capacity-building interventions on resilience

**Fig. 5:**
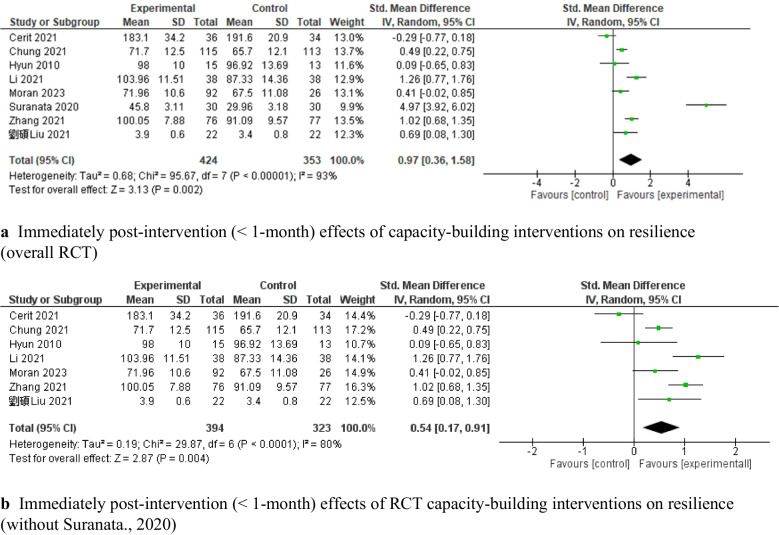
Immediately post-intervention effects (< 1-month) of RCT capacity-building interventions on resilience

Eight c-RCT studies [63, 69, 73, 79, 82, 90, 95, 101] demonstrated that capacity-building interventions had a significant pooled effect on improving resilience, with moderate heterogeneity (*SMD* = 0.20, 95% *CI:* [0.05, 0.36], *P* < 0.05, 8 c-RCTs, 2,533 adolescents, *I*^*2*^ = 60%; Fig. 4a). A leave-one-out analysis of these eight c-RCT studies indicated that the study by Connor et al. [90] was the main contributor of heterogeneity. After we excluded this study, the intervention effect remained significant, with low heterogeneity (*SMD* = 0.27, 95% *CI*: [0.18, 0.35], *P* < 0.00001, 7 c-RCTs, 2,275 adolescents, *I*^*2*^ = 0%; Fig. 4b). The levels of positive mental health reported by Connor et al. demonstrated relatively high psychological functioning at baseline, with only 2% of participants categorized as experiencing languishing mental health; thus, the lack of efficacy might be attributed to a ceiling effect, that is, early adolescents with high baseline scores could have limited potential for improvement. Eight RCT studies [67, 68, 75, 83, 85, 87, 99, 107] also demonstrated significant improvements in resilience (*SMD* = 0.97, 95% *CI*: [0.36, 1.58], *P* = 0.002, 8 RCTs, 777 adolescents Fig. 5a), with high heterogeneity (*I*^*2*^ = 93%). The leave-one-out sensitive analysis identified that the study by Suranata et al. [99] was the greatest contributor to this high heterogeneity. After we removed this study, the pooled effect was reduced but remained significant (*SMD* = 0.54, 95% *CI*: [0.17, 0.91], *P* < 0.05, 7 RCTs, 717 adolescents, *I*^*2*^ = 80%; Fig. 5b). The leave-one-out analysis did not result in any significant reductions in heterogeneity.

**Fig. 6:**
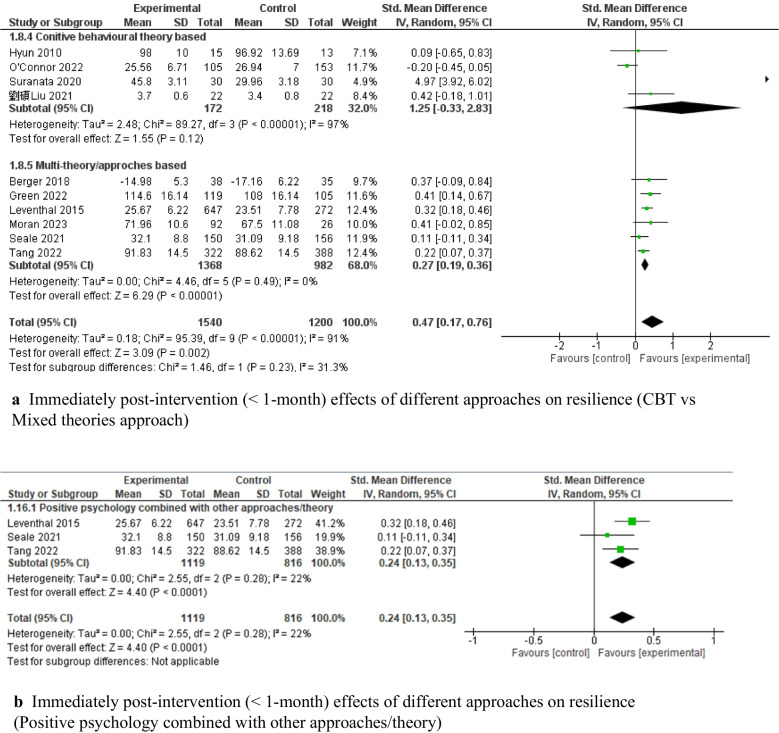

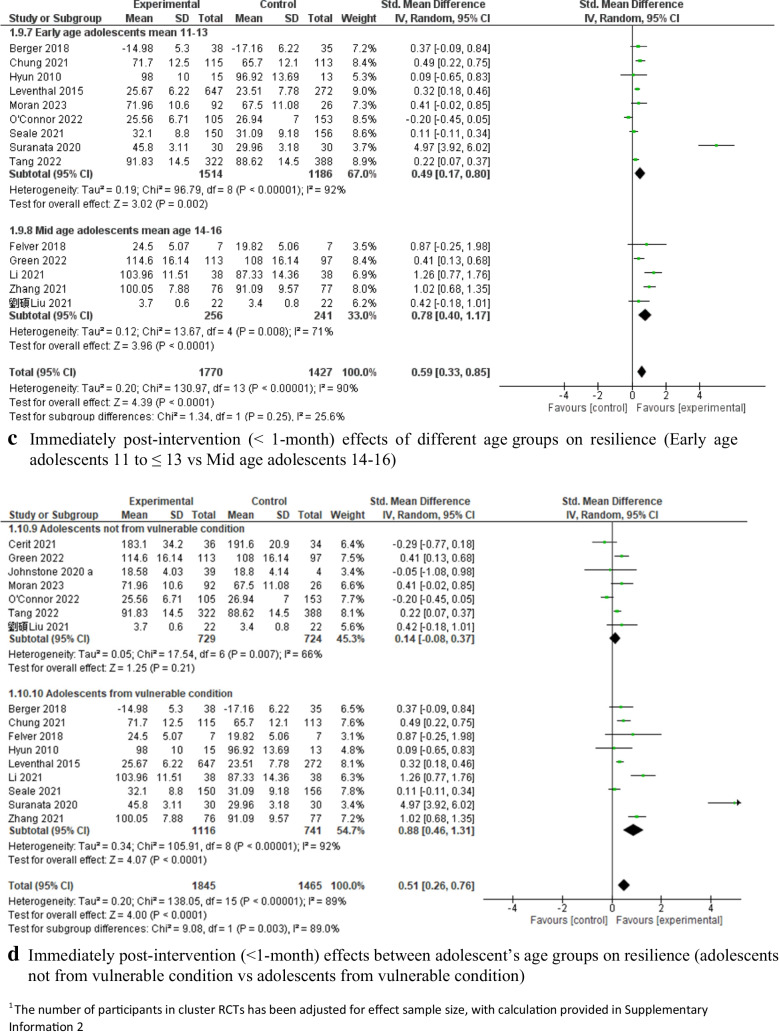
Immediately post-intervention effects of capacity-building interventions on resilience

The effectiveness of the interventions was then stratified according to the interventions’ theory and the adolescents’ characteristics. Interventions combining multiple theories [63, 73, 82, 87, 95, 101] showed a significant effect on improving resilience (*SMD* = 0.27, 95% *CI*: [0.19, 0.36], *P* < 0.001, 5 c-RCTs and 1 RCT, 2,350 adolescents, *I*^*2*^ = 0; Fig. 6a), while cognitive-behavioral therapy (CBT)-based interventions [75, 85, 90, 99] showed a nonsignificant pooled effect (*SMD* = 1.25, 95% *CI*: [−0.33, 2.83], *P* > 0.05, 3 RCTs and 1 c-RCT, 390 adolescents, *I*^*2*^ = 97%; Fig. 6a). Among the combined interventions based on multiple theoretical models/frameworks, three studies integrated positive psychology theory with emotional intelligence, spirituality, and social cognitive theory, respectively; they showed significant pooled effects on improving resilience (*SMD* = 0.24, 95% *CI*: [0.13, 0.35], *P* < 0.001, 3 c-RCTs, 1,935 adolescents, *I*^*2*^ = 22%; Fig. 6b). Capacity-building interventions focused on resilience showed significant effects on resilience for both early adolescents aged 11–13 (*SMD* = 0.49, 95% *CI*: [0.17, 0.80], *P* < 0.001, 5 c-RCTs and 4 RCTs, 2,700 adolescents, *I*^*2*^ = 92%) [63, 68, 75, 82, 87, 90, 95, 99, 101] and middle adolescents aged 14–16 (*SMD* = 0.78, 95% *CI*: [0.40, 1.17], *P* < 0.001, 2 c-RCTs and 3 RCTs, 497 adolescents, *I*^*2*^ = 71%; see Fig. 6c) [69, 73, 83, 85, 107].

There was a significant pooled effect on improving resilience among vulnerable adolescents (e.g., those who were ethnic minorities, lived in impoverished or under-resourced environments, or had lower baseline scores) (*SMD* = 0.88, 95% *CI*: [0.46, 1.31], *P* < 0.001), with substantial heterogeneity (*I*^*2*^ = 92%) [63, 68, 69, 75, 82, 83, 95, 99, 107]. In contrast, no significant pooled effect on improving resilience was observed among the adolescents who were not from vulnerable conditions (*SMD* = 0.14, 95% *CI*: [−0.08, 0.37], *P* > 0.05, *I*^*2*^ = 66%; Fig. 6d) [67, 73, 79, 85, 87, 90, 101].

**Fig. 7:**
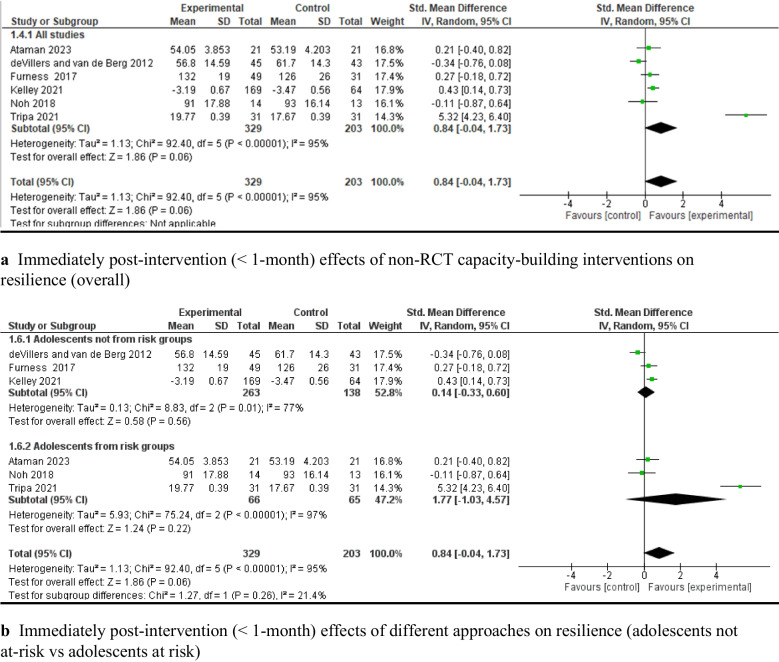
Immediately post-intervention effects of non-RCT capacity-building interventions on resilience: overall non-RCT studies (**a**) and based on adolescent’s characteristics (**b**)

The meta-analysis of six quasi-experimental studies [62, 72, 81, 89, 102, 105] showed that capacity-building interventions had a nonsignificant effect on resilience (*SMD* = 0.84, 95% *CI*: [−0.04, 1.73], *P* > 0.05, 505 adolescents; see Fig. 7a). A subgroup analysis also showed a nonsignificant pooled effect on improving at-risk adolescents’ resilience (e.g., those who were ethnic minorities or lived in an under-resourced environment) (see Fig. 7b).

### Medium-term effectiveness of the interventions (1–6 months post-intervention)

**Fig. 8:**
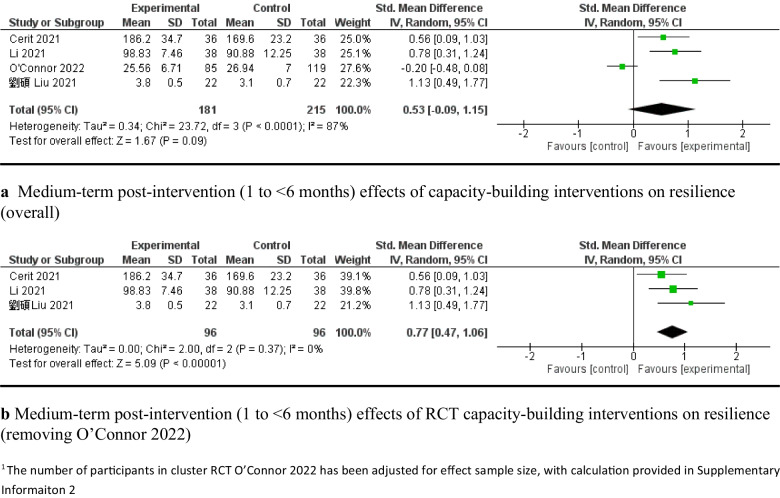
Medium-term post-intervention (1 to < 6 months) effects on resilience: overall (**a**), after removing O’Connor 2022 **(b)**

The medium-term (1–6 months) effectiveness of the capacity-building interventions for adolescents was only assessed on resilience because of the lack of comparable interventions for mental health literacy. Four studies evaluated the medium-term effects of the interventions on resilience [67, 83, 85, 90]. The lengths of the follow-ups ranged from 1 to 3 months post-intervention. The meta-analysis of these four RCTs studies indicated a nonsignificant pooled effect of capacity-building interventions on resilience compared with the control groups, such as school-as-usual (*SMD* = 0.53, 95% *CI*: [−0.09, 1.15], *P* > 0.05, 3 RCTs and 1 c-RCT, 396 adolescents; Fig. 8a). The c-RCT study by O’Connor [90] was the main source of heterogeneity, and the pooled effect size became significant after this study was removed (*SMD* = 0.77, 95% *CI*: [0.47, 1.06], *P* < 0.001, 3 RCTs, 192 adolescents, *I*^*2*^ = 0). Therefore, the nonsignificant result might stem from the ceiling effect observed in O’Connor’s study [90].

### Long-term effectiveness of the interventions (≥ 6 months post-intervention)

**Fig. 9:**
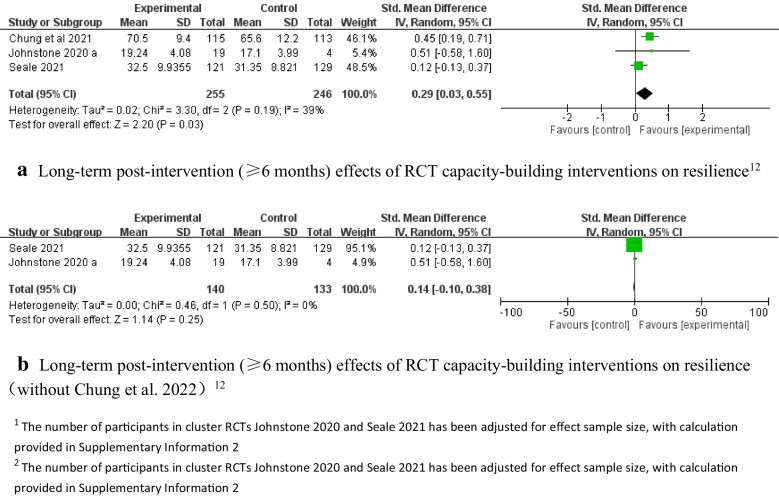
Long-term post-intervention (≥ 6 months post-intervention) effects on resilience

Three studies evaluated the long-term effects of the capacity-building interventions (≥ 6 months post-intervention) for adolescents’ resilience [68, 79, 95]. The meta-analysis indicated a small but significant pooled effect on improving resilience in the intervention groups compared with the control groups (*SMD* = 0.29, 95% *CI*: [0.03, 0.55], *P* = 0.03, 2 c-RCTs and 1 RCT, 501 adolescents, moderate heterogeneity *I*^*2*^ = 39%; Fig. 9a). When the study by Chung et al. was excluded [68], the pooled results indicated no significant effect (*SMD* = 0.14, 95% *CI*: [−0.10, 0.38], *P* = 0.50; Fig. 9b) derived from two c-RCTs involving 501 adolescents. Notably, there was no observed heterogeneity among the studies (*I*^*2*^ = 0). The significant pooled effect might have been primarily influenced by the findings of Chung et al., which focused on risk games conducted over a brief duration of two days and one night. It is possible that such a short intervention period could not adequately represent the broader context of the research, indicating an outlier effect of that study in the analysis.

## Secondary outcomes

### Depression

**Fig. 10:**
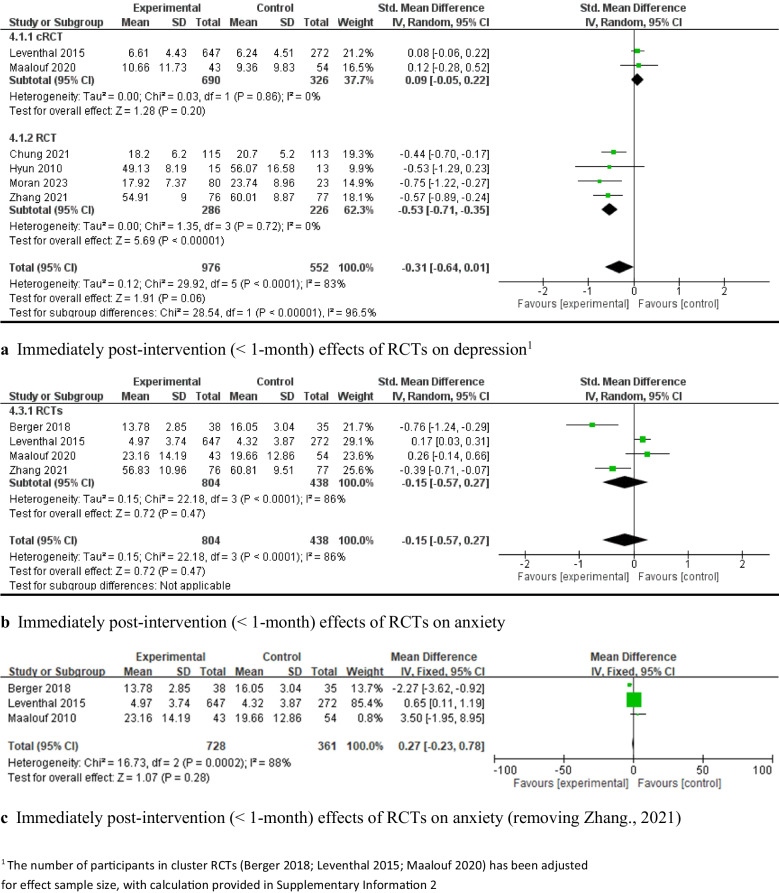
Meta-analyses results of capacity-building interventions on depression and anxiety

Six studies assessing resilience-focused or self-efficacy-focused capacity-building interventions on depression immediately post-intervention were included in the meta-analysis. The overall results revealed that resilience-focused or self-efficacy-focused capacity-building interventions had a nonsignificant pooled effect on reducing depression symptoms (*SMD* = −0.31, 95% *CI*: [−0.64, 0.01], *P* = 0.16, 4 RCTs and 2 c-RCTs, 1,528 adolescents; Fig. 10a). However, the meta-analysis stratified by study design showed a significant reduction in depression among the four RCTs, with low heterogeneity (*SMD* = −0.53, 95% *CI*: [−0.71, −0.35], *P* < 0.001, *I*^*2*^ = 0; Fig. 10a), while the pooled effect was nonsignificant in the two c-RCTs (*SMD* = 0.09, 95% *CI*: [−0.05, 0.22], *P* > 0.05, *I*^*2*^ = 0).

### Anxiety

Four RCT studies evaluating resilience-focused capacity-building interventions on anxiety immediately post-intervention showed a nonsignificant pooled effect on anxiety reduction (*SMD* = −0.15, 95% *CI*: [−0.57, 0.27], *P* > 0.05, 1 RCT and 3 c-RCTs, 1,242 adolescents,* I*^*2*^ = 86; Fig. 10b). When conducting a leave-one-out sensitive analysis by removing Zhang's study [107], the result still showed a nonsignificant pooled effect on anxiety reduction with high heterogeneity (*SMD* = 0.27, 95% *CI*: [−0.23, 0.78], *P* > 0.05, 3 c-RCTs, 728 adolescents; Fig. 10c).

## Outcomes of individual studies (Narrative analysis)

Whenever there were insufficient studies (< 3) for a pooled analysis of effectiveness, we extracted the study data and calculated the effect sizes for individual outcomes of studies (Table 3).

**Table 3:** Effect of capacity building intervention on study outcomes not pooled in the meta-analysis

Outcome	Study	Instrument			Effect size^a^, SMD (95%CI)^b^	
					Post-inervention	1–18 months(m) post-intervention
Mental health literacy	Casanas 2022	EMHL Test	Recognition		0.64 [0.46, 0.82]	6 m 0.43 [0.26, 0.61] 12 m 0.43 [0.25, 0.60]
			Knowledge		0.73 [0.55, 0.91]	6 m 0.48 [0.30, 0.66] 12 m 0.31 [0.14, 0.49]
	Fraser 208	MHKA-Researcher defined	knowledge		0.77 [0.14, 1.40] ***	2 m 1.43 [0.52, 2.34] **
			Awareness		−0.13 [−0.74, 0.47] *	2 m 0.18 [−0.43, 0.79] **
	Yamaguchi 2020	MHKA-Researcher defined			1.58 [1.43, 1.74] ***	2 m 0.85 [0.71, 1.00] ***
	Skre 2013	MHKA			N/A	N/A
	Fretian 2023	MHKS			0.77 [0.45, 1.09] ***	N/A
	Simkiss 2023	MHKAS			N/A	N/A
	Ionescu 2023	MHKAS	Knowledge		0.04 [−0.19, 0.27] ***	N/A
			Attitudes		−0.08 [−0.30, 0.15] **	N/A
	Ibrahim 2020	MHSAS			0.30 [−0.12, 0.71] **	N/A
	Hassen 2022	MHLq			0.35[0.154–0.542] ***	N/A
	Patafino 2021	MHLS			N/A	N/A
	Morgago 2021	QuALiSMental			N/A	N/A
	Vella 2021	D-lit/A-lit	Depression-literacy		0.63 [0.32, 0.94] ***	N/A
			Anxiety-literacy		0.00 [−0.31, 0.31] ***	N/A
**Resilience**	**Imet 2023**	**APRS**			0.95***	N/A
	**Kallianta 2021**	**CYRM**			N/A	N/A
	**Noh 2018**	**YKRQ-27**			−0.11 [−0.87, 0.64]	1 m −0.12 [−0.89, 0.65]
	**O’Cornor 2022**	**CDRS-10**			−0.19 [−0.37, −0.01]	1.5 m −0.40 [−0.60, −0.20]
	**Sahin 2021**	**GURGAN’S Resilience Scale**			N/A	N/A
	**Shelton 2006**	**Polk-20**			N/A	N/A
	**SuraN/Ata 2020**	**RYDM**			0.89***	N/A
**Self-efficacy**	Cepukiene 2018	SES		General self-efficacy	0.53 [0.00, 1.05] ***	N/A
				Social self-efficacy	0.57 [−0.19, 1.33]	N/A
	Furness 2017	Project-K Self-efficacy Questionnaire		Academic self-efficacy	0.52 [0.24, 0.80] *	N/A
				Social self-efficacy	0.38 [0.13,0.62] *	N/A
				Help seeking self-efficacy	0.30 [0.16, 0.44] *	N/A
	Fretian 2023	HSES			0.56 [0.25, 0.88] ***	N/A
	Leventhal 2015	GSES			2.51 [1.88, 3.1] ***	N/A
	Moran 2023	SEQ-C			−0.08 [−0.53, 0.37]	N/A
	Tak 2014	SEQ			−0.19 [−0.30, −0.08]	18 m 0.12 [0.01, 0.23]
**Positive thinking**	**Robinson 2015**	**ATQ-P**			0.49***	N/A

^***^, P < 0.001; **, P < 0.01; *, P < 0.05; N/A = Not reported; EMHL = = EspaiJove Mental health Literacy MHKA = Knowledge about mental health, MHKS = Mental Health Knowledge Scale, MHKAS = Mental Health Knowledge and Attitudes Scale, MHSAS = Mental Help Seeking Attitude Scale, MHLq = Mental Health Literacy Questionnaire, MHLS = Mental health literacy scale, QuALiSMental = Questionnaire for Assessment of Mental Health Literacy, D-lit/A-lit = Depression Literacy Questionnaire & Anxiety Literacy Questionnaire, APRS = Adolescent psychological resilience scale, CYRM = Child and Youth Resilience Measure, YKRQ-27 = Youth Korea Resilience Quotient-27, CDRS = Connor-Davidson Resilience Scale, Polk-20 = Polk Resilience Patterns Scale-20, RYDM = = Indonesian version of Resilience Youth Development Module, SES = Self-Efficacy Scale, HSES = Help-seeking efficacy scale, GSES = Schwarzer's General Self-Efficacy Scale, SEQ = Self-Efficacy Questionnaire, ATQ-P = = Positive Automatic Thoughts Questionnaire

### Mental health literacy

Studies focusing on mental health literacy exhibited considerable heterogeneity in intervention duration, follow-up times, and measurement tools. Of the 16 studies concerning mental health literacy, only four were pooled into a meta-analysis because of sufficient similarity and data integrity. Twelve studies [65, 70, 71, 74, 76, 78, 88, 91, 97, 98, 104, 106] assessed capacity-building interventions aimed at enhancing mental health literacy among adolescents. Ten of these studies [65, 70, 71, 78, 88, 91, 97, 98, 104, 106] implemented in-person mental health psychoeducational programs combined with training in self-help strategies and coping skills. Three studies [65, 70, 78] focused on the subdomain of mental health literacy (i.e., recognition and knowledge). Only Casanas’s study [65] assessed long-term effects (6 months and beyond). Notably, the effect size observed at 6 months (SMD = 0.43, 95% CI: [0.26, 0.61]) was identical to that at 12 months (SMD = 0.43, 95% CI: [0.25, 0.60]); however, this equivalence was not statistically significant. In contrast, two studies [71] that evaluated medium-term effects (at 2 months) showed significant improvements compared with the control group. Fraser’s study [70] focused on multiple outcomes simultaneously, examining both mental health literacy (SMD = 0.77, 95% CI: [0.45, 1.09], P < 0.001) and self-efficacy (SMD = 0.56, 95% CI: [0.25, 0.88], P < 0.001), and reported significant improvements in both outcomes.

### Resilience

Regarding the outcomes for resilience [77, 80, 89, 90, 94, 96, 99]. the seven studies used a total of seven distinct assessment tools.

### Self-resilience

Seven RCTs [66, 71, 72, 82, 87, 93, 100] evaluated self-efficacy as an outcome in capacity-building interventions aimed at promoting mental health. Some of the interventions specifically targeted the enhancement of self-efficacy [66, 72], while others included self-efficacy as one of several outcome measures. These studies varied in the domains of self-efficacy that they addressed, such as academic self-efficacy, help-seeking self-efficacy, self-efficacy for coping, general self-efficacy, and social self-efficacy. A quasi-RCT study by Cepukiene et al. [66] utilizing positive developmental theory reported significant improvements in general self-efficacy (SMD = 0.53, 95% CI: [0.00, 1.05], P < 0.001) but not in social self-efficacy (SMD = 0.57, 95% CI: [−0.19, 1.33], P = 0.176).

### Positive thinking

One RCT study by Robinson et al. [78] examined a CBT-based intervention with stress management training and found that the intervention significantly improved African American adolescents’ level of positive thinking immediately post-intervention compared with a typical school curriculum without any psychoeducational skill training (Cohen’s d = 0.49, P < 0.001). Because only one study attained this outcome, the result should be interpreted with caution.

## Evidence quality of the studies included in the meta-analysis

The funnel plot and Egger’s test (*P* = 0.115) indicated no risk of publication bias, suggesting that the studies were representative (see Supplementary Information, SI 5); however, because of the low quality of evidence in the 27 included studies (see SI 6), we caution against over-interpreting the meta-analysis results and emphasize the need for more rigorous, high-quality research to strengthen the conclusions.

## Discussion

A systematic review based on 47 studies was conducted to synthesize evidence on capacity-building interventions designed to enhance adolescents’ mental health through the promotion of mental health literacy, resilience, self-efficacy, and positive thinking. Overall, these interventions demonstrated significant positive effects on mental health literacy and resilience in short- to long-term follow-ups. Subgroup analyses indicated that interventions based on multi-theoretical frameworks consisting of multiple components such as group education, cognitive and coping skills training, and behavioral management strategies were the most effective at improving adolescents’ resilience and mental health literacy. In contrast, the few studies addressing self-efficacy and positive thinking produced inconclusive findings on their related outcomes.

Most of the mental health literacy interventions focused on concepts of mental illness such as understanding and recognition of mental disorders, but only a few studies [2, 71, 97] addressed topics or concepts related to positive mental health. This trend may reflect the lack of a comprehensive understanding of mental health, which, as the World Health Organization has stated, is more than the absence of mental disorders [110]. Effective interventions were characterized as multi-component and face-to-face group psychoeducation on topics such as mental illness and its care, symptom recognition, stigma, and help-seeking behaviors. Gender differences were also observed, with female adolescents generally showing greater gains than their male counterparts, particularly in single-sex interventions, as seen in the studies by Liddle (only male participants) [84] and Zare (only female participants) [2]. However, this difference might also stem from the durations of the interventions: Liddle’s study [84] involved only a 45-min workshop, whereas Zare’s [2] intervention lasted for six weeks. Therefore, we cannot yet draw reliable conclusions about gender differences in mental health literacy interventions. The immediate post-intervention effects might be caused by direct engagement and mental health knowledge provided during interventions, which emphasizes the acquisition of knowledge as a crucial step toward initial behavior change [25]. Even mental health literacy showed improvements after just a single mental health knowledge session. Ibrahim’s study [76] conducted a single-session intervention involving a lecture and video on depression for 101 students at an NGO-managed boarding school. This intervention led to improvements in depression literacy, with a small but significant effect that persisted at the 3-month follow-up (ηp^2^ = 0.048, P < 0.05). Liddle’s [84] single workshop, featuring psychoeducation and group discussion, significantly boosted mental health knowledge among 102 adolescents from a community sports club, demonstrating the effectiveness of even brief interventions. This finding can offer valuable insights into the design of future interventions, allowing for the optimization of both their duration and components. While most studies concentrated on general awareness and knowledge, the others [104] examined specific areas such as depression and anxiety specific literacy, which presented challenges in synthesizing the findings. Although prior studies contribute valuable insights into mental health literacy, achieving methodological consistency is crucial for advancing this field of research. A unified approach will facilitate not only better comparisons across studies but also the formulation of evidence on effective interventions to improve mental health literacy in diverse adolescent populations.

Capacity-building interventions effectively promoted adolescents’ resilience in the short and long term, albeit with a noticeable reduction in their effects over time (similar to the above-mentioned benefits on mental health literacy). This finding was different from those of previous reviews, which indicated that resilience-focused programs for children and adolescents can have sustained effects for up to six months [111]. Short-term capacity-building interventions [63, 67–69, 73, 75, 79, 82, 83, 85, 87, 90, 95, 99, 101, 107] significantly improved adolescent resilience within one month post-intervention, with notable gains in the intervention groups versus the control groups. However, the high study heterogeneity suggests significant variability in the results. Assessments of the medium-term (1–6 months) [67, 83, 85, 90] and long-term (≥ 6 months) [68, 79, 95] effects were relatively scarce, and the medium-term effects were not significant. When study by O'Connor et al. [90] were removed, the effect of strength-based capacity-building interventions on resilience showed a statistically significant difference. We observed that among the four studies examining medium-term effects, only the population in O'Connor's study consisted of elementary school students(median age 11), while the other three studies [67, 83, 85] involved high school students(median age 14–16). This age difference might contribute to the disparity in findings. Further analysis revealed that the baseline resilience level in O'Connor's study was relatively high, which could make it more challenging to achieve significant improvements compared to studies with lower baseline levels. This highlights that although the World Health Organization defines adolescents as those aged 10–19 [112], different ages within this stage may exhibit varying resilience levels. Future research should consider age differences and baseline resilience levels, ensuring these factors are fully reported. There was a small but significant long-term effect for resilience. However, the effects of the interventions tended to decline over time; thus, without reinforcements and further support, the positive effects of these psychoeducation programs will not be sustainable [64]. Continuous education and/or booster sessions can be used to sustain the benefits in mental health literacy and the related concepts of non-stigmatizing attitudes and wellbeing [113]. Challenges in maintaining the interventions’ effectiveness may stem from the dynamic nature of resilience, which involves complex interactions between protective factors that enable individuals to face adversity [114]. Resilience develops over time through repeated interactions with one’s environment. Adolescence is a critical period characterized by significant developmental shifts. As adolescents grow, their needs and challenges change, requiring adaptive interventions. Strategies that were effective in early adolescence may become less relevant as they transition into later stages, leading to diminishing intervention effects. Intervention components such as self-awareness, social skills, family support, community resources, and adventure programs interact in complex ways. While these interventions enhance resilience, their effectiveness can vary over time. For example, high self-awareness might initially lead to better resource utilization, but without ongoing support or new challenges, this effect can wane. Protective intervention components often reinforce each other in feedback loops. For instance, participating in extracurricular activities can boost self-esteem, which encourages further personal growth. If these activities or opportunities decrease over time, the reinforcing cycle weakens, potentially reducing resilience. Subgroup analysis revealed that different study designs, theoretical frameworks, and types of adolescent populations influence intervention effectiveness. For example, c-RCTs exhibited moderate heterogeneity, and after the removal of a study was likely affected by a ceiling effect (leading to nonsignificant outcomes), the intervention effect remained significant, with low heterogeneity. Our results showed that adolescents with specific risk characteristics, such as those from ethnic minorities and lower socioeconomic status or with lower baseline resilience scores, might exhibit greater improvements in resilience shortly after the capacity-building interventions. In subsequent subgroup analyses, vulnerable adolescents [63, 68, 69, 75, 82, 83, 95, 99, 107] showed significant positive effects from the interventions, whereas non-vulnerable adolescents [67, 73, 79, 85, 87, 90, 101] did not demonstrate notable improvements. Furthermore, interventions with combined or multiple components, particularly those integrating positive psychology with other psychological health approaches, proved most effective in enhancing short-term (< 1 month) resilience. However, the findings of this review did not support the effectiveness of CBT-based interventions in improving adolescents’ resilience, in contrast to the latest systematic review on resilience interventions [115]. This discrepancy might be attributable to the focus of this review on resilience as a capacity for adaptation, whereas most CBT-based interventions aimed to improve emotional symptoms [116]. In our review, four studies [75, 85, 90, 99] on resilience utilized CBT-based interventions. Interventions that focused solely on cognitive biases and discussions of mental health were less effective than the control group in boosting resilience [90]. CBT-based cognitive restructuring showed only a modest effect on resilience [75]. Conversely, studies that incorporated cognitive skills alongside social skills training and family support as part of broader coping strategies resulted in substantial improvements in resilience [85, 99]. This indicates that interventions emphasizing comprehensive coping strategies, including social and family support, may be more beneficial for enhancing adolescents’ resilience compared with those focusing exclusively on cognitive aspects [117]. Additionally, studies using CBT approaches in our review were rated as having a moderate or high risk of bias, particularly regarding randomization and outcome data completeness, potentially skewing the results. This not only underscores the importance of tailoring interventions to individual needs but also highlights the need for more efficient resource allocation to ensure that the most vulnerable adolescents receive priority in mental health support. Such targeted interventions not only helped enhance psychological resilience in the short term but also created a robust foundation for resilience.

Although a previous study on the mediation of self-efficacy reported that self-efficacy and positive thinking were correlated with adolescents’ mental health [118], this review found few studies specifically using these constructs to enhance mental health outcomes. Because of the limited number of comparable studies and varied measurement tools (SES/GSES/Project K self-efficacy), we were unable to pool the results on self-efficacy and positive thinking in the included studies for meta-analysis. Only two included studies [66, 72] prioritized self-efficacy in their interventions, while others treated it merely as one of many secondary outcomes. The participants in the solution-focused self-efficacy enhancement group intervention for adolescents reported a significant increase in general self-efficacy with a large effect size compared with the control group. However, the intervention did not have a significant effect on social self-efficacy. Moreover, interventions using group-based, solution-focused approaches (e.g., short-term goal-focused practice), participatory activities (e.g., outdoor activities), and peer mentorship were more effective in improving adolescents’ self-efficacy than the individual-based intervention of 6-week sessions of health coaching, in which adolescents met one-on-one with a coach [87]. According to Bandura’s theory, self-efficacy can be bolstered through mastery experiences, verbal persuasion, and vicarious experiences, which is consistent with other studies investigating factors influencing adolescents’ self-efficacy [40, 119]. These findings suggest that interventions focused on providing vicarious reinforcements and supportive peer experiences and recognition can effectively enhance adolescents’ self-efficacy in mental health promotion. One study adopting self-determination theory combined with a youth resilience model had low attendance because of the COVID-19 pandemic and did not significantly improve self-efficacy [87]. Conversely, interventions grounded in positive psychology and restorative practices achieved significant success among rural adolescents [82]. This suggests that choosing the right theoretical framework is crucial for intervention success. Different frameworks may be better suited to adolescents of diverse types or backgrounds; thus, careful consideration of the target population’s characteristics is necessary when designing interventions. Interventions in settings such as outdoor activities and community challenges significantly boosted self-efficacy, demonstrating that supportive and motivating environments can enhance effectiveness and provide unique experiences that aid skill development and confidence-building in adolescents [72]. While we referred to “positive thinking” in this review, it is important to note that this concept was not defined consistently across studies. Various studies have highlighted that the term encompasses a range of constructs, including optimism and hope, which may serve as more precise alternatives in future research [120]. For instance, optimism is characterized by generalized expectations of positive outcomes and has been linked to better psychological health and resilience. Similarly, hope has been shown to drive adaptive behavior and improve coping mechanisms during stressful situations [121].

Overall, the outcomes of self-efficacy and positive thinking as part of capacity-building interventions for adolescent mental health are profoundly influenced by multiple factors, including intervention design, external environment, and long-term sustainability. External conditions and implementation settings significantly impact intervention effectiveness, and flexible and adaptable designs are necessary. It may be better to use self-efficacy training in combination with other trainings in cognitive and behavioral constructs such as positive thinking, coping, and resilience to provide more comprehensive and substantial benefits. A multi-faceted approach that combines cognitive-behavioral techniques with other therapeutic modalities (such as mindfulness, social skills, or adventure programs) may enhance intervention effectiveness. Four RCTs [68, 75, 87, 107] demonstrated a significant reduction in depression with low heterogeneity, whereas two c-RCTs [82, 86] did not show significant effects. This discrepancy may be explained by the influence of intra-cluster correlations and the heightened sensitivity of c-RCTs to contextual factors like school culture, peer influence, and administrative support.

## Limitations and implications

This review has several limitations.

First, there was substantial heterogeneity among the included studies in terms of their study designs, sample sizes, theoretical frameworks, types of interventions, and lengths of follow—ups. This heterogeneity posed challenges to data synthesis and undermined the credibility of the pooled evidence. The medium-term effect of resilience became significant after removing O'Connor's study from the meta-analysis, which had previously shown non-significant results. This finding highlights the need for cautious consideration of the robustness of these results.

Second, this study included quasi-experimental studies, which may lead to selection bias and reporting bias. Significant risks of bias were identified in the studies, particularly due to inadequate allocation concealment and the lack of blinding during outcome assessments. These issues likely introduced systematic errors, undermining the internal validity of the studies and potentially skewing their findings. Additionally, the narrative synthesis itself may have been limited by the variability in study methodologies and outcomes, which could affect the overall conclusions drawn from this review.

Third, our meta-analyses were limited to evaluating the short-term effects of the interventions, as the studies mainly had follow-up periods of less than 1 month. Longer-term evaluation of the effectiveness of the interventions with a larger and more diverse sample is recommended to provide more conclusive evidence on sustainable benefits for adolescents’ mental health.

Fourth, while our exclusion criteria focused on adolescents with clinically diagnosed mental health disorders, it is important to note that non-selected populations, such as those recruited from public schools, may include subgroups with significant levels of mental distress [112]. These adolescents, though not formally diagnosed, may exhibit symptom levels above clinical cut-offs, potentially influencing intervention outcomes. Future research should assess and report baseline symptom levels to better understand how capacity-building interventions impact adolescents across the mental health spectrum, from those with subclinical symptoms to those with more pronounced distress.

Fifth, we only included Chinese and English literature, which may introduce certain biases. Future studies are recommended to have no language limitations. Due to the long interval between preregistration and the conduct of the systematic review, our manuscript has some deviations from the preregistration. To maintain consistency, we have updated the preregistration. Additionally, we have provided Table (SI 7) to present the deviations between the preregistration and this manuscript, along with the reasons for these changes.

Finally, although our research referenced the World Health Organization's definition of adolescents as those aged 10–19, some studies suggest that a definition of 10–24 years corresponds more closely to adolescent growth and general understandings of this life phase. Future research could consider expanding the definition of adolescents to this broader age range [122].

Strength-based capacity-building interventions can simultaneously enhance mental health literacy, resilience, self-efficacy, and positive thinking, as these four elements are closely interrelated and mutually reinforcing. Improved mental health literacy may aid in the recognition and management of psychological issues, thereby boosting resilience. Enhanced resilience can strengthen the ability to cope with challenges, increasing self-efficacy, and greater self-efficacy may empower individuals to face difficulties more confidently, fostering positive thinking. Positive thinking can reinforce all of these elements, creating a virtuous cycle that comprehensively boosts psychological capital and overall mental health. The findings of this review offer preliminary insights that can inform practice and policy regarding capacity-building interventions for adolescents. First, capacity-building interventions should be guided by combined or multiple approaches or components of psychological theories/models, such as psychoeducation, cognitive and resilience training, and psycho-behavioral elements, to effectively enhance resilience and mental health literacy. Addressing problem-solving skills and cultural adaptability curriculum and activities could be particularly beneficial for boosting self-efficacy and fostering positive thinking. Second, continuous education, reinforcement, and support are crucial to sustain these benefits. Capacity-building interventions can allocate less time to knowledge acquisition and more time to building mental health awareness and literacy, prioritizing deepened understanding and sustained benefits. The effects of mental health interventions can diminish over time; to sustain resilience, interventions should be adaptable, continuously reinforced, and tailored to the changing needs of adolescents as they grow and face new challenges. By precisely identifying and responding to the specific needs of individual adolescents, we can maximize the effectiveness of interventions and ensure that resources are directed where they can have the greatest impact.

Policymakers are encouraged to provide support and allocate resources to integrative multicomponent capacity-building programs for adolescents in community and educational settings. Ultimately, the long-term collaborative efforts of governments, schools, communities, and families are essential to promote adolescents’ mental health through improving their resilience, mental health literacy, self-efficacy, and positive thinking, thus contributing to healthier future generations. Given the limitations in the quality of the studies included in this review, these insights should be considered thoughtfully and alongside other evidence to guide decision-making.

## Conclusion

This systematic review and meta-analysis provides evidence that strength-based capacity-building interventions that are underpinned by the principles of positive psychology, resilience, and other psychological approaches can significantly enhance adolescents’ resilience and mental health in the short term (e.g., < 3 months post-intervention). The therapeutic components of these interventions, mainly including psychoeducation and cognitive and behavioral training, are best delivered in schools and group-based settings, fostering a supportive environment for adolescents. However, the observed high heterogeneity and varied methodological quality of the included studies raised concerns regarding the risk of bias and the generalizability of the pooled results. To fill the gaps in knowledge on this topic, future research should prioritize and include more positive mental health elements (mental health literacy, resilience, self-efficacy, and positive thinking), as well as gender-specific and person-centered approaches, in capacity-building interventions to enable further evaluation of their long-term effects, particularly on mental health literacy, resilience, and overall well-being. Rigorous high-quality randomized controlled trials will be essential in clarifying the nuances of how these interventions can be optimally designed, implemented, and evaluated over a reasonably long follow-up period. In summary, this review highlights the transformative potential of strength-based capacity-building interventions for adolescents, setting the stage for the further exploration and development of an effective evidence-based capacity-building intervention for fostering adolescents’ resilience and mental well-being.

## Supplementary Information

Below is the link to the electronic supplementary material.

Supplementary file1 (DOCX 914 KB)

## Data Availability

Data is provided within the manuscript or supplementary information files.

## References

[1] World Health Organization (2024) The Adolescent health indicators recommended by the Global Action for Measurement of Adolescent health: Guidance for Monitoring Adolescent Health at Country, Regional and Global Levels. World Health Organization. https://www.who.int/publications/i/item/9789240092198. Accessed 10 June 2024

[2] Zare S, Kaveh MH, Ghanizadeh A, Asadollahi A, Nazari M (2021) Promoting mental health literacy in female students: a school-based educational intervention. Health Educ J80(6):734–745. 10.1177/00178969211013571

[3] Stoerkel E(2019) What Is a Strength-Based Approach. Positivepsychology. https://positivepsychology.com/strengths-based-interventions/#strength-based-approach. Accessed 12 March 2024

[4] World Health Organization (2021) Adolescent Mental Health. World Health Organization. https://www.who.int/news-room/fact-sheets/detail/adolescent-mental- health. Accessed 10 October 2024

[5] Braun-Lewensohn O, Idan O, Lindström B and Margalit M (2022) Salutogenesis and the sense of coherence during the adolescent years. In: Mittelmark MB, Bauer GF, Vaandrager L et al (eds) The handbook of salutogenesis. 2nd edn. Springer, Cham, pp 139–150 36121985

[6] Damon W (2004) What is positive youth development? Ann Am Acad Political Soc Sci 591:13–14. 10.1177/0002716203260092

[7] (Md) R(2023) National healthcare quality and disparities report. Agency for Healthcare Research and Quality (US). https://www.ncbi.nlm.nih.gov/books/NBK600459/. Accessed December 2023 38377267

[8] Solmi M, Radua J, Olivola M, Croce E, Soardo L, Salazar De Pablo G, Il Shin J, Kirkbride JB, Jones P, Kim JH (2022) Age at onset of mental disorders worldwide: large-scale meta-analysis of 192 epidemiological studies. Mol Psychiatry 27(1):281–295. 10.1038/s41380-021-01161-7 34079068 10.1038/s41380-021-01161-7 PMC8960395

[9] Lereya ST, Patel M, Dos Santos JPGA, Deighton J (2019) Mental health difficulties, attainment and attendance: a cross-sectional study. Eur Child Adolesc Psychiatry 28:1147–1152. 10.1007/s00787-018-01273-6 30627786 10.1007/s00787-018-01273-6

[10] Schlack R, Peerenboom N, Neuperdt L, Junker S, Beyer A-K (2021) The effects of mental health problems in childhood and adolescence in young adults: Results of the KiGGS cohort. J Health Monit 6(4):3. 10.25646/8863 35146318 10.25646/8863 PMC8734087

[11] Santre S (2022) Mental health promotion in adolescents. J Indian Assoc Child Adolesc Ment Health 18(2):122–127. 10.1177/09731342221120709

[12] Sadownik AR (2023) Bronfenbrenner: ecology of human development in ecology of collaboration. In: Sadownik AR, and Višnjić JA (eds) (Re)theorising more-than-parental involvement in early childhood education and care (International Perspectives on Early Childhood Education and Development, 40). 1st ed. Springer, Cham, pp 83–95

[13] Brownlee K, Rawana J, Franks J, Harper J, Bajwa J, O’brien E, Clarkson A (2013) A systematic review of strengths and resilience outcome literature relevant to children and adolescents. Child Adolesc Soc Work J30:435–459. 10.1007/s10560-013-0301-9

[14] Lerner RM (2009) The positive youth development perspective: theoretical and empirical bases of strengths based approach to adolescent development. In: Lopez SJ, and Snyder CR (eds) Oxford handbook of positive psychology. 2nd edn. Oxford Academic, Oxford, pp 149–164

[15] Lopez SJ, Louis MC (2009) The principles of strengths-based education. J Coll Character 10(4):1-8. 10.2202/1940-1639.1041

[16] Murphy AL, Gardner DM, Kutcher SP, Martin-Misener R (2014) A theory-informed approach to mental health care capacity building for pharmacists. Int J Ment Health Syst 8:1–11. 10.1186/1752-4458-8-46 25473416 10.1186/1752-4458-8-46 PMC4254206

[17] Bazyk S, Demirjian L, Laguardia T, Thompson-Repas K, Conway C, Michaud P (2015) Building capacity of occupational therapy practitioners to address the mental health needs of children and youth: A mixed-methods study of knowledge translation. Am J Occup Ther 69(6):1–10. 10.5014/ajot.2015.019182 10.5014/ajot.2015.019182 PMC4643378 26565099

[18] Kowalenko N, Hagali M, Hoadley B (2020) Building capacity for child and adolescent mental health and psychiatry in Papua New Guinea. Australas Psychiatry 28(1):51–54. 10.1177/1039856219871883 31486670 10.1177/1039856219871883

[19] Semrau M, Alem A, Abdulmalik J, Docrat S, Evans-Lacko S, Gureje O, Kigozi F, Lempp H, Lund C, Petersen I (2018) Developing capacity-building activities for mental health system strengthening in low-and middle-income countries for service users and caregivers, service planners, and researchers. Epidemiol Psychiatr Sci 27(1):11–21. 10.1017/S2045796017000452 28965528 10.1017/S2045796017000452 PMC6998877

[20] Zhang X, Yue H, Hao X, Liu X, Bao H (2023) Exploring the relationship between mental health literacy and psychological distress in adolescents: A moderated mediation model. Prev Med Rep 33:102199. 10.1016/j.pmedr.2023.102199 37223554 10.1016/j.pmedr.2023.102199 PMC10201844

[21] Steven JC (2008) Strengths based counseling and positive thinking/learned optimism. Basic Counseling Skills. Retrieved from https://www.basic-counseling-skills.com/strengths-based.html. Accessed 2024

[22] Caiels J, Silarova B, Milne AJ, Beadle-Brown J (2023) Strengths-based Approaches—Perspectives from Practitioners. Br J Soc Work 54(1):168–188. 10.1093/bjsw/bcad186

[23] Kågström A, Juríková L, Guerrero Z (2023) Developmentally appropriate mental health literacy content for school-aged children and adolescents. Camb Prisms Glob Ment Health 10:e25. 10.1017/gmh.2023.16 10.1017/gmh.2023.16 PMC10579665 37854395

[24] Kutcher S, Bagnell A, Wei Y (2015) Mental health literacy in secondary schools: a Canadian approach. Child Adolesc Psychiatr Clin 24(2):233–244. 10.1016/j.chc.2014.11.007 10.1016/j.chc.2014.11.007 25773321

[25] Jorm AF (2012) Mental health literacy: empowering the community to take action for better mental health. Am Psychol 67(3):231. 10.1037/a0025957 22040221 10.1037/a0025957

[26] Lam LT (2014) Mental health literacy and mental health status in adolescents: a population-based survey. Child Adolesc Psychiatry Ment Health 8:1–8. 10.1186/1753-2000-8-2624444351

[27] Gulliver A, Griffiths KM, Christensen H (2010) Perceived barriers and facilitators to mental health help-seeking in young people: a systematic review. BMC Psychiatry 10:1–9. 10.1186/1471-244X-10-113 21192795 10.1186/1471-244X-10-113 PMC3022639

[28] Masten AS (2011) Resilience in children threatened by extreme adversity: Frameworks for research, practice, and translational synergy. Dev Psychopathol 23(2):493–506. 10.1017/S0954579411000198 23786691 10.1017/S0954579411000198

[29] Olsson CA, Bond L, Burns JM, Vella-Brodrick DA, Sawyer SM (2003) Adolescent resilience: A concept analysis. J Adolesc 26(1):1–11. 10.1016/s0140-1971(02)00118-5 12550818 10.1016/s0140-1971(02)00118-5

[30] Fletcherd S (2013) Psychologicalresilience: Areviewand critiqueofdefinitions, concepts, andtheory. Eur Psychol 18(1):12–23. 10.1027/1016-9040/a000124

[31] Dray J, Bowman J, Campbell E, Freund M, Wolfenden L, Hodder RK, Mcelwaine K, Tremain D, Bartlem K, Bailey J (2017) Systematic review of universal resilience-focused interventions targeting child and adolescent mental health in the school setting. J Am Acad Child Adolesc Psychiatry 56(10):813–824. 10.1016/j.jaac.2017.07.780 28942803 10.1016/j.jaac.2017.07.780

[32] Lee TY, Cheung CK, Kwong WM (2012) Resilience as a positive youth development construct: a conceptual review. Sci World J 1:390450. 10.1100/2012/390450 10.1100/2012/390450 PMC3353472 22623893

[33] Galatzer-Levy IR, Huang SH, Bonanno GA (2018) Trajectories of resilience and dysfunction following potential trauma: A review and statistical evaluation. Clin Psychol Rev 63:41–55. 10.1016/j.cpr.2018.05.008 29902711 10.1016/j.cpr.2018.05.008

[34] Kalisch R, Müller MB, Tüscher O (2015) A conceptual framework for the neurobiological study of resilience. Behav Brain Sci 38:e92. 10.1017/s0140525x1400082x 25158686 10.1017/S0140525X1400082X

[35] Fergus S, Zimmerman MA (2005) Adolescent resilience: A framework for understanding healthy development in the face of risk. Annu Rev Public Healt 26(1):399–419. 10.1146/annurev.publhealth.26.021304.144357 10.1146/annurev.publhealth.26.021304.144357 15760295

[36] Boden JM, Sanders J, Munford R, Liebenberg L, Mcleod GF (2016) Paths to positive development: A model of outcomes in the New Zealand youth transitions study. Child Indic Res 9:889–911. 10.1007/s12187-015-9341-3

[37] Benight CC, Bandura A (2004) Social cognitive theory of posttraumatic recovery: The role of perceived self-efficacy. Behav Res Ther 42(10):1129–1148. 10.1016/j.brat.2003.08.008 15350854 10.1016/j.brat.2003.08.008

[38] Bandura A (2006) Guide for constructing self-efficacy scales. In: Pajares F, Urdan T (eds) Self-efficacy beliefs of adolescents. Information Age Publishing, Greenwich, pp 307–337

[39] Saarni C (1999) The development of emotional competence. Guilford, London

[40] Kleppang AL, Steigen AM, Finbråten HS (2023) Explaining variance in self-efficacy among adolescents: the association between mastery experiences, social support, and self-efficacy. BMC Public Health 23(1):1665. 10.1186/s12889-023-16603-w 37648980 10.1186/s12889-023-16603-w PMC10466853

[41] Dray J (2021) Child and adolescent mental health and resilience-focussed interventions: A conceptual analysis to inform future research. Int J Environ Res Public Health 18(14):7315. 10.3390/ijerph18147315 34299765 10.3390/ijerph18147315 PMC8303353

[42] Caprara GV, Steca P, Gerbino M, Paciello M, Vecchio GM (2006) Looking for adolescents’ well-being: Self-efficacy beliefs as determinants of positive thinking and happiness. Epidemiol Psychiatr Sci 15(1):30–43. 10.1017/s1121189x00002013 10.1017/s1121189x00002013 16584101

[43] Moksnes UK, Eilertsen MEB, Ringdal R, Bjørnsen HN, Rannestad T (2019) Life satisfaction in association with self-efficacy and stressor experience in adolescents–self-efficacy as a potential moderator. Scand J Caring Sci 33(1):222–230. 10.1111/scs.12624 30374994 10.1111/scs.12624

[44] Yuan J, Ju E, Meng X, Chen X, Zhu S, Yang J, Li H (2015) Enhanced brain susceptibility to negative stimuli in adolescents: ERP evidences. Front Behav Neurosci 9:98. 10.3389/fnbeh.2015.00098 25972790 10.3389/fnbeh.2015.00098 PMC4412063

[45] Leeman J, Calancie L, Kegler MC, Escoffery CT, Herrmann AK, Thatcher E, Hartman MA, Fernandez ME (2017) Developing theory to guide building practitioners’ capacity to implement evidence-based interventions. Health Educ Behav 44(1):59–69. 10.1177/1090198115610572 26500080 10.1177/1090198115610572 PMC5330318

[46] Bergeron K, Abdi S, Decorby K, Mensah G, Rempel B, Manson H (2017) Theories, models and frameworks used in capacity building interventions relevant to public health: a systematic review. BMC Public Health 17:1–12. 10.1186/s12889-017-4919-y 29183296 10.1186/s12889-017-4919-y PMC5706342

[47] Bekhet AK, Zauszniewski JA (2013) Measuring use of positive thinking skills: Psychometric testing of a new scale. West J Nurs Res 35(8):1074–1093. 10.1177/0193945913482191 23509101 10.1177/0193945913482191

[48] Washburn IJ, Acock A, Vuchinich S, Snyder F, Li K-K, Ji P, Day J, Dubois D, Flay BR (2011) Effects of a social-emotional and character development program on the trajectory of behaviors associated with social-emotional and character development: Findings from three randomized trials. Prev Sci 12:314–323. 10.1007/s11121-011-0230-9 21720782 10.1007/s11121-011-0230-9

[49] Charoensuk S (2007) Negative thinking: A key factor in depressive symptoms in Thai adolescents. Issues Ment Health Nurs 28(1):55–74. 10.1080/01612840600996265 17130007 10.1080/01612840600996265

[50] Chui RC, Chan C-K (2020) Positive thinking, school adjustment and psychological well-being among Chinese college students. Open Psychol J 13(1):151. 10.2174/1874350102013010151

[51] Page MJ, Mckenzie JE, Bossuyt PM, Boutron I, Hoffmann TC, Mulrow CD, Shamseer L, Tetzlaff JM, Akl EA, Brennan SE (2021) The PRISMA 2020 statement: an updated guideline for reporting systematic reviews. BMJ 372:n71. 10.1136/bmj.n71 33782057 10.1136/bmj.n71 PMC8005924

[52] Innovation VH(n.d.) Covidence systematic review software. Melbourne, Australia. https://www.covidence.org/

[53] Ryan R, Synnot A, Prictor M and Hill S(2020) Cochrane consumers and communication group data extraction template for included studies. La Trobe University, Melbourne. https://cccrg.cochrane.org/author-resources. Accessed 29 November 2024

[54] Eldridge S, Campbell M, Campbell M, Dahota A, Giraudeau B, Higgins J, Reeves B, Siegfried N (2016) Revised Cochrane risk of bias tool for randomized trials (RoB 2.0): additional considerations for cluster-randomized trials. https://methods.cochrane.org/bias/resources/rob-2-revised-cochrane-risk-bias-tool-randomized-trials. Accessed 29 November 2024

[55] Sterne JA, Hernán MA, Reeves BC, Savović J, Berkman ND, Viswanathan M, Henry D, Altman DG, Ansari MT, Boutron I (2016) ROBINS-I: a tool for assessing risk of bias in non-randomised studies of interventions. BMJ 355:i4919. 10.1136/bmj.i4919 27733354 10.1136/bmj.i4919 PMC5062054

[56] Higgins Jpt TJ, Chandler J, Cumpston M, Li T, Page Mj, Welch Va (Editors)(2024) Cochrane handbook for systematic reviews of interventions version 6.5. Cochrane. https://training.cochrane.org/handbook/current/chapter-23. Accessed 29 November 2024

[57] Guyatt G, Oxman AD, Akl EA, Kunz R, Vist G, Brozek J, Norris S, Falck-Ytter Y, Glasziou P, Debeer H (2011) GRADE guidelines: 1. Introduction—GRADE evidence profiles and summary of findings tables. J Clin Epidemiol 64(4):383–394. 10.1016/j.jclinepi.2010.04.026 21195583 10.1016/j.jclinepi.2010.04.026

[58] Valentine JC, Pigott TD, Rothstein HR (2010) How many studies do you need? A primer on statistical power for meta-analysis. J Educ Behav Stat 35(2):215–247. 10.3102/1076998609346961

[59] Cohen J (1992) Statistical power analysis. Curr Dir Psychol Sci 1(3):98–101

[60] Dersimonian R, Kacker R (2007) Random-effects model for meta-analysis of clinical trials: an update. Contemp Clin Trials 28(2):105–114. 10.1016/j.cct.2006.04.004 16807131 10.1016/j.cct.2006.04.004

[61] Higgins JP, Thompson SG (2002) Quantifying heterogeneity in a meta-analysis. Stat Med 21(11):1539–1558. 10.1002/sim.1186 12111919 10.1002/sim.1186

[62] Ataman A, Uysal B (2023) Examining the effectiveness of a group hope intervention program in Syrian refugee children: A pilot study. Vulnerable Child Youth Stud 18(3):501–515. 10.1080/17450128.2023.2216476

[63] Berger R, Benatov J, Cuadros R, Vannattan J, Gelkopf M (2018) Enhancing resiliency and promoting prosocial behavior among Tanzanian primary-school students: A school-based intervention. Transcult Psychiatry 55(6):821–845. 10.1177/1363461518793749 30091688 10.1177/1363461518793749

[64] Campos L, Dias P, Duarte A, Veiga E, Dias CC, Palha F (2018) Is It Possible to “Find Space for Mental Health” in Young People? Effectiveness of a School-Based Mental Health Literacy Promotion Program. Int Int J Environ Res Public Health 15(7):1426. 10.3390/ijerph15071426 29986444 10.3390/ijerph15071426 PMC6069495

[65] Casañas R, Castellvi P, Gil JJ, Torres-Torres M, Barón J, Teixidó M, Sampietro HM, Díez M, Fernández R, Sorli R, Siñol P, Jurado F, Carreras-Salvador R, Vazquez D, Gonzalez S, Martín MIF, Raya-Tena A, Alvarez R, Amado-Rodriguez I, López LMM, Alonso J, Lalucat-Jo L (2022) The effectiveness of a “EspaiJove.net”- a school-based intervention programme in increasing mental health knowledge, help seeking and reducing stigma attitudes in the adolescent population: a cluster randomised controlled trial. BMC Public Health 22(1):2425. 10.1186/s12889-022-14558-y 36566192 10.1186/s12889-022-14558-y PMC9789578

[66] Cepukiene V, Pakrosnis R, Ulinskaite G (2018) Outcome of the solution-focused self-efficacy enhancement group intervention for adolescents in foster care setting. Child Youth Serv Rev 88:81–87. 10.1016/j.childyouth.2018.03.004

[67] Cerit E, Şimşek N (2021) A social skills development training programme to improve adolescents’ psychological resilience and emotional intelligence level. Arch Psychiatr Nurs 35(6):610–616. 10.1016/j.apnu.2021.08.001 34861953 10.1016/j.apnu.2021.08.001

[68] Chung JOK, Li WHC, Ho KY, Lam KKW, Cheung AT, Ho LLK, Lin JJ, Lopez V (2021) Adventure-based training to enhance resilience and reduce depressive symptoms among juveniles: A randomized controlled trial. Res Nurs Health 44(3):438–448. 10.1002/nur.22127 33754400 10.1002/nur.22127

[69] Felver JC, Clawson AJ, Morton ML, Brier-Kennedy E, Janack P, Diflorio RA (2019) School-based mindfulness intervention supports adolescent resiliency: A randomized controlled pilot study. Int J Sch Educ Psychol 7(sup1):111–122. 10.1080/21683603.2018.1461722

[70] Fraser E, Pakenham KI (2008) Evaluation of a resilience-based intervention for children of parents with mental illness. Aust N Z J Psychiatry 42(12):1041–1050. 10.1080/00048670802512065 19016092 10.1080/00048670802512065

[71] Freţian AM, Kirchhoff S, Bauer U, Okan O (2023) The effects of an adapted mental health literacy curriculum for secondary school students in Germany on mental health knowledge and help-seeking efficacy: results of a quasi-experimental pre-post evaluation study. Front Public Health 11:1219925. 10.3389/fpubh.2023.1219925 37663825 10.3389/fpubh.2023.1219925 PMC10468570

[72] Furness K, Williams MN, Veale J, Gardner DH (2017) Maximising potential: The psychological effects of the youth development programme Project K. N Z J Psychol 46(1):14–23

[73] Green AL, Ferrante S, Boaz TL, Kutash K, Wheeldon-Reece B (2022) Effects of the SPARK Teen Mentoring Program for high school students. J Child Fam Stud 31(7):1982–1993. 10.1007/s10826-022-02298-x

[74] Hassen HM, Behera MR, Jena PK, Dewey RS,Disassa GA (2022) Effectiveness and implementation outcome measures of mental health curriculum intervention using social media to improve the mental health literacy of adolescents. J Multidiscip Healthc 15:979–997. 10.2147/JMDH.S361212 10.2147/JMDH.S361212 PMC9078434 35535244

[75] Hyun M-S, Nam KA, Kim M-A (2010) Randomized controlled trial of a cognitive–behavioral therapy for at-risk Korean male adolescents. Arch Psychiatr Nurs 24(3):202–211. 10.1016/j.apnu.2009.07.005 20488346 10.1016/j.apnu.2009.07.005

[76] Ibrahim N, Mohd Safien AI, Siau CS, Shahar S (2020) The effectiveness of a depression literacy program on stigma and mental help-seeking among adolescents in Malaysia: a control group study with 3-month follow-up. INQUIRY-J Health Car 57:0046958020902332. 10.1177/0046958020902332 10.1177/0046958020902332 PMC7288814 32009506

[77] İme Y, Ümmet D (2024) The effects of cognitive behavioral psychological group counseling program on the psychological resilience and emotional flexibility of adolescents. Curr Psychol 43(10):8885–8895. 10.1007/s12144-023-05051-9

[78] Ionescu-Corbu A, Ursu A (2023) The sustained effectiveness of a mental health literacy intervention: a romanian adolescent sample at 2-and 12-months follow-Up. J Innov Psychol Educ Didact 27(1):43–58

[79] Johnstone KM, Middleton T, Kemps E, Chen J (2020) A pilot investigation of universal school-based prevention programs for anxiety and depression symptomology in children: A randomized controlled trial. J Clin Psychol 76(7):1193–1216. 10.1002/jclp.22926 31943189 10.1002/jclp.22926

[80] Kallianta M-DK, Katsira XE, Tsitsika AK, Vlachakis D, Chrousos G, Darviri C,Bacopoulou F (2021) Stress management intervention to enhance adolescent resilience: a randomized controlled trial. EMBnet J 26(1):e967. 10.14806/ej.26.1.967 10.14806/ej.26.1.967 PMC8525873 34671547

[81] Kelley T, Kessel A, Collings R, Rubenstein B, Monnickendam C, Solomon A (2021) Evaluation of the iHEART mental health education programme on resilience and well-being of UK secondary school adolescents. J Public Ment Health 20(1):43–50. 10.1108/JPMH-03-2020-0019

[82] Leventhal KS, Gillham J, Demaria L, Andrew G, Peabody J, Leventhal S (2015) Building psychosocial assets and wellbeing among adolescent girls: A randomized controlled trial. J Adolesc 45:284–295. 10.1016/j.adolescence.2015.09.011 26547145 10.1016/j.adolescence.2015.09.011

[83] Li C, Ma C, Li P, Liang Z (2022) The effect of model-based group counseling on the resiliency of disadvantaged adolescents from poor areas of china: a single-blind randomized controlled study. Sch Ment Health 14(3):550–567. 10.1007/s12310-021-09479-x

[84] Liddle SK, Deane FP, Batterham M, Vella SA (2021) A brief sports-based mental health literacy program for male adolescents: A cluster-randomized controlled trial. J Appl Sport Psychol 33(1):20–44. 10.1080/10413200.2019.1653404

[85] Liu S, Wang Y (2021) Using cognitive behavioral group counseling to improve the mental toughness of high school students. Chin J Clin Psychol 29:198–202

[86] Maalouf FT, Alrojolah L, Ghandour L, Afifi R, Dirani LA, Barrett P, Nakkash R, Shamseddeen W, Tabaja F, Yuen CM (2020) Building emotional resilience in youth in Lebanon: a school-based randomized controlled trial of the FRIENDS intervention. Prev Sci 21:650–660. 10.1007/s11121-020-01123-5 32363411 10.1007/s11121-020-01123-5

[87] Moran MJ, Aichele S, Shomaker LB, Lucas-Thompson RG, Heberlein E, Chandrasekhar JL, Bowen AE, Kaar JL (2024) Supporting youth mental health through a health coaching intervention with a mindfulness component: A pilot randomized controlled trial during COVID-19. Child Youth Care Forum 53(3):645–666. 10.1007/s10566-023-09764-7

[88] Morgado T, Loureiro L, Rebelo Botelho MA, Marques MI, Martínez-Riera JR, Melo P (2021) Adolescents’ empowerment for mental health literacy in school: a pilot study on ProLiSMental psychoeducational intervention. Int J Environ Res Public Health 18(15):8022. 10.3390/ijerph18158022 34360315 10.3390/ijerph18158022 PMC8345420

[89] Noh D (2018) The effect of a resilience enhancement programme for female runaway youths: A quasi-experimental study. Issues Ment Health Nurs 39(9):764–772. 10.1080/01612840.2018.1462871 30239243 10.1080/01612840.2018.1462871

[90] O’connor M, O’reilly G, Murphy E, Connaughton L, Hoctor E, Mchugh L (2022) Universal process-based CBT for positive mental health in early adolescence: A cluster randomized controlled trial. Behav Res Ther 154:104120. 10.1016/j.brat.2022.104120 35659695 10.1016/j.brat.2022.104120

[91] Patafio B, Skvarc D, Miller P, Hyder S (2021) Evaluating a sport-based mental health literacy intervention in Australian amateur sporting adolescents. J Youth Adolesc 50(12):2501–2518. 10.1007/s10964-021-01513-0 34626293 10.1007/s10964-021-01513-0

[92] Perry Y, Petrie K, Buckley H, Cavanagh L, Clarke D, Winslade M, Hadzi-Pavlovic D, Manicavasagar V, Christensen H (2014) Effects of a classroom-based educational resource on adolescent mental health literacy: A cluster randomised controlled trial. J Adolesc 37(7):1143–1151. 10.1016/j.adolescence.2014.08.001 25151646 10.1016/j.adolescence.2014.08.001

[93] Robinson WL, Droege JR, Case MH, Jason LA (2015) Reducing stress and preventing anxiety in African American adolescents: a culturally-grounded approach. Glob J Community Psychol Pract 6(2):1-12. 10.7728/0602201503 10.7728/0602201503 PMC4813807 27042702

[94] Şahin H, Türk F (2021) The impact of cognitive-behavioral group psycho-education program on psychological resilience, irrational beliefs, and well-being. J Ration Emot Cogn Behav Ther 39(4):672–694. 10.1007/s10942-021-00392-5 33824549 10.1007/s10942-021-00392-5 PMC8016428

[95] Seale JP, Seale DM, Pande Y, Lewis TM, Manda W, Kasanga L, Gibson EB, Hadfield K, Ogoh T, Mcgrath RE, Harris SK (2022) GROW Zambia: A pilot cluster-randomized trial of a spiritually-based character strengths training curriculum to enhance resilience among Zambian youth. J Posit Psychol 17(4):596–609. 10.1080/17439760.2021.1913640

[96] Shelton D, Lyon-Jenkins N (2006) Mental health promotion for vulnerable African American youth. J Forensic Nurs 2(1):7–14. 10.1111/j.1939-3938.2006.tb00048.x 17073393 10.1111/j.1939-3938.2006.tb00048.x

[97] Simkiss NJ, Gray NS, Kemp AH, Dunne C, Snowden RJ (2023) A randomised controlled trial evaluating the Guide Cymru mental health literacy intervention programme in year 9 (age 13–14) school pupils in Wales. BMC Public Health 23(1):1062. 10.1186/s12889-023-15922-2 37277757 10.1186/s12889-023-15922-2 PMC10239719

[98] Skre I, Friborg O, Breivik C, Johnsen LI, Arnesen Y, Wang CEA (2013) A school intervention for mental health literacy in adolescents: effects of a non-randomized cluster controlled trial. BMC Public Health 13:1–15. 10.1186/1471-2458-13-873 24053381 10.1186/1471-2458-13-873 PMC3850725

[99] Suranata K, Rangka IB, Permana AAJ (2020) The comparative effect of internet-based cognitive behavioral counseling versus face to face cognitive behavioral counseling in terms of student’s resilience. Cogent Psychology 7(1):1751022. 10.1080/23311908.2020.1751022

[100] Tak YR, Kleinjan M, Lichtwarck-Aschoff A, Engels RC (2014) Secondary outcomes of a school-based universal resiliency training for adolescents: a cluster randomized controlled trial. BMC Public Health 14:1–13. 10.1186/1471-2458-14-1171 25404142 10.1186/1471-2458-14-1171 PMC4247712

[101] Tang Y, Diao H, Jin F, Pu Y, Wang H (2022) The effect of peer education based on adolescent health education on the resilience of children and adolescents: A cluster randomized controlled trial. PLoS ONE 17(2):e0263012. 10.1371/journal.pone.0263012 35108312 10.1371/journal.pone.0263012 PMC8809556

[102] Tripa L, Sava FA, Paloş R, Măgurean S, Macsinga I (2021) Evaluating the outcomes of “Resilient left-behind children”–A social-emotional learning and mindfulness group counseling program. Cogn Brain Behav 25(1):33. 10.24193/cbb.2021.25.03

[103] Tuijnman A, Kleinjan M, Hoogendoorn E, Granic I, Engels RC (2019) A game-based school program for mental health literacy and stigma regarding depression (moving stories): protocol for a randomized controlled trial. JMIR Res Protoc 8(3):e11255. 10.2196/11255 30869652 10.2196/11255 PMC6437615

[104] Vella SA, Swann C, Batterham M, Boydell KM, Eckermann S, Ferguson H, Fogarty A, Hurley D, Liddle SK, Lonsdale C (2021) An intervention for mental health literacy and resilience in organized sports. Med Sci Sports Exerc 53(1):139. 10.1249/MSS.0000000000002433 32555025 10.1249/MSS.0000000000002433 PMC7737879

[105] Villiers M, Van Den Berg H (2012) The implementation and evaluation of a resiliency programme for children. S Afr J Psychol 42:93–102. 10.1177/008124631204200110

[106] Yamaguchi S, Ojio Y, Foo JC, Michigami E, Usami S, Fuyama T, Onuma K, Oshima N, Ando S, Togo F (2020) A quasi-cluster randomized controlled trial of a classroom-based mental health literacy educational intervention to promote knowledge and help-seeking/helping behavior in adolescents. J Adolesc 82:58–66. 10.1016/j.adolescence.2020.05.002 32615487 10.1016/j.adolescence.2020.05.002

[107] Zhang J, Zhou Z, Zhang W (2021) Intervention effect of research-based psychological counseling on adolescents’mental health during the covid-19 epidemic. Psychiatr Danub 33(2):209–216. 10.24869/psyd.2021.209 34185752 10.24869/psyd.2021.209

[108] Miller CJ, Smith SN, Pugatch M (2020) Experimental and quasi-experimental designs in implementation research. Psychiatry Res 283:112452. 10.1016/j.psychres.2019.06.027 31255320 10.1016/j.psychres.2019.06.027 PMC6923620

[109] Elliott SA, Brown JS (2002) What are we doing to waiting list controls? Behav Res Ther 40(9):1047–1052. 10.1016/s0005-7967(01)00082-1 12296489 10.1016/s0005-7967(01)00082-1

[110] Mansfield R, Patalay P, Humphrey N (2020) A systematic literature review of existing conceptualisation and measurement of mental health literacy in adolescent research: current challenges and inconsistencies. BMC Public Health 20:1–14. 10.1186/s12889-020-08734-1 32357881 10.1186/s12889-020-08734-1 PMC7195735

[111] Pinto TM, Laurence PG, Macedo CR, Macedo EC (2021) Resilience programs for children and adolescents: a systematic review and meta-analysis. Front Psychol 12:754115. 10.3389/fpsyg.2021.754115 34880812 10.3389/fpsyg.2021.754115 PMC8645691

[112] World Health Organization(2024) Mental health of adolescents. World Health Organization. https://www.who.int/news-room/fact-sheets/detail/adolescent-mental-health. Accessed 10 October 2024

[113] Richards BA, Frankland PW (2017) The persistence and transience of memory. Neuron 94(6):1071–1084. 10.1016/j.neuron.2017.04.037 28641107 10.1016/j.neuron.2017.04.037

[114] Liebenberg L (2020) Reconsidering interactive resilience processes in mental health: Implications for child and youth services. J Community Psychol 48(5):1365–1380. 10.1002/jcop.22331 32058584 10.1002/jcop.22331

[115] Llistosella M, Goni-Fuste B, Martín-Delgado L, Miranda-Mendizabal A, Franch Martinez B, Pérez-Ventana C, Castellvi P (2023) Effectiveness of resilience-based interventions in schools for adolescents: A systematic review and meta-analysis. Front Psychol 14:1211113. 10.3389/fpsyg.2023.1211113 37868613 10.3389/fpsyg.2023.1211113 PMC10587685

[116] Wu Y, Fenfen E, Wang Y, Xu M, Liu S, Zhou L, Song G, Shang X, Yang C, Yang K, Li X (2023) Efficacy of internet-based cognitive-behavioral therapy for depression in adolescents: A systematic review and meta-analysis. Internet Interv 34:100673. 10.1016/j.invent.2023.100673 37822787 10.1016/j.invent.2023.100673 PMC10562795

[117] Thompson NJ, Fiorillo D, Rothbaum BO, Ressler KJ, Michopoulos V (2018) Coping strategies as mediators in relation to resilience and posttraumatic stress disorder. J Affect Disord 225:153–159. 10.1016/j.jad.2017.08.049 28837948 10.1016/j.jad.2017.08.049 PMC5626644

[118] Schönfeld P, Brailovskaia J, Bieda A, Zhang XC, Margraf J (2016) The effects of daily stress on positive and negative mental health: Mediation through self-efficacy. Int J Clin Health Psychol 16(1):1–10. 10.1016/j.ijchp.2015.08.005 30487845 10.1016/j.ijchp.2015.08.005 PMC6225043

[119] Wang Y, Li S, Gong J, Cao L, Xu D, Yu Q, Wang X, Chen Y (2022) Perceived Stigma and Self-Efficacy of Patients With Inflammatory Bowel Disease-Related Stoma in China: A Cross-Sectional Study. Front Med 9:813367. 10.3389/fmed.2022.813367 10.3389/fmed.2022.813367 PMC8888524 35252252

[120] Laranjeira C, Querido A (2022) Hope and optimism as an opportunity to improve the “positive mental health” demand. Front Psychol 13:827320. 10.3389/fpsyg.2022.827320 35282230 10.3389/fpsyg.2022.827320 PMC8907849

[121] Almeida TC, Ifrim IC (2023) Psychometric properties of the positive thinking skills scale (PTSS) among Portuguese adults. Behav Sci 13(5):357. 10.3390/bs13050357 37232594 10.3390/bs13050357 PMC10215308

[122] Sawyer SM, Azzopardi PS, Wickremarathne D, Patton GC (2018) The age of adolescence. The Lancet Child Adolesc Health 2(3):223–228. 10.1016/S2352-4642(18)30022-1 30169257 10.1016/S2352-4642(18)30022-1

