# Supplementary material for: Strength-based capacity-building interventions to promote adolescents’ mental health: A systematic review and meta-analysis
Source: Eur Child Adolesc Psychiatry. 2025 May 28;34(10):2975–3015. doi: 10.1007/s00787-025-02741-6 (PMC12592288; doi:10.1007/s00787-025-02741-6)
Supplement: Supplementary file 1 — Supplementary file1 (DOCX 914 KB) [file 787_2025_2741_MOESM1_ESM.docx]

**Supplementary Information**

**List of contents**

[Supplementary Information 1 Full search strategy 2](#_Toc176940257)

[Supplementary Information 2 Effective sample size calculation （accounting for clustering in meta-analysis of cluster randomized controlled trials） 7](#_Toc176940258)

[Supplementary Information 3 The overall risk of bias for all included studies 10](#_Toc176940259)

[Supplementary Information 4 The risk of bias for each study 13](#_Toc176940260)

[Supplementary Information 5: Funnel plot 28](#_Toc176940261)

[Supplementary Information 6: Grade Assessment 29](#_Toc176940262)

# **Supplementary Information 1 Full search strategy**

**Table 1-1 Search strings in PubMed**

| PICO framework |  | Search terms | Search hits |
| --- | --- | --- | --- |
| ***Population***  (Adolescent:10-19 years old following WHO definition) | #1 | adolescen*[Title/Abstract] OR teen*[Title/Abstract] OR youth*[Title/Abstract] OR juvenile*[Title/Abstract] OR (adolescent [Mesh]) | 2,457,786 |
| ***Intervention***  (Any intervention aims to build capacity for adolescents from mental health literacy, resilience, self-efficacy, and positive thinking) | #2 | (capacit*[Text Word] OR competenc*[Text Word] OR capabilit*[Text Word] OR ("skill development"[Text Word] OR "skill building" [Text Word]) OR strength*[Text Word] OR empower*[Text Word]  OR (resilience [Text Word] OR "Resilience, Psychological"[Mesh])  OR ("self-efficacy"[Text Word] OR "Self Efficacy"[Mesh])  OR ("Health Literacy"[Mesh] OR "mental health literacy"[Text Word])  OR ("positive thinking"[Text Word] OR "positive thoughts"[Text Word] OR "Optimism"[Mesh] | 1,970,023 |
| ***Outcome***  (Mental health literacy, positive thinking, resilience and coping) | #3 | ("coping*" [Text Word] OR "Adaptation, Psychological"[Mesh])  OR (resilience [Text Word] OR "Resilience, Psychological"[Mesh])  OR ("Health Literacy"[Mesh] OR "mental health literacy"[Text Word]) OR ("positive thinking"[Text Word] OR "positive thoughts"[Text Word] OR "Optimism"[Mesh]) OR ("self-efficacy"[Text Word] OR "Self Efficacy"[Mesh]) | 120,617 |
|  | #4 | #1 AND #2 AND #3 | 20,104 |
| ***Study design*** | #5 | (randomis*[Title/Abstract] OR randomiz*[Title/Abstract] OR trial[Title/Abstract] OR experiment*[Title/Abstract]) OR ("Clinical Trials as Topic"[Mesh]) OR ("randomized controlled trial"[Publication Type] OR "controlled clinical trial"[Publication Type]) | 4,269,834 |
|  | #6 | #4 AND #5 | 3,505 |
|  | #7 | #6 AND Filters: from 2003-2023, Chinese, English | 3,173 |

**Table 1-2 Search strings in CINAHL Ultimate**

| PICO framework |  | Search terms | Search hits |
| --- | --- | --- | --- |
| ***Population***  (Adolescent:10-19 years old following WHO definition) | #1 | TI adolescen* OR AB adolescen* OR TI youth* OR AB youth* TI juvenile* OR AB juvenile* OR TI teen* OR AB teen* OR MH "Adolescence+" | 683,668 |
| ***Intervention***  (Any intervention aims to build capacity for adolescents from mental health literacy, resilience, self-efficacy, and positive thinking) | #2 | TX capacit* OR TX competenc* OR TX capabilit* OR ("skill developemnt" OR "skill building") OR TX strength* OR TX empower* OR MH "Empowerment"  OR TX resilience OR (MH "Hardiness")  OR TX "self-efficacy" OR (MH "Self-Efficacy")  OR TX "mental health literacy" OR (MH "Health Literacy")  OR TX "positive thinking" OR TX "positive thoughts" OR MH "Optimism" | 1,354,781 |
| ***Outcome***  (Mental health literacy, positive thinking, resilience and coping) | #3 | TX coping* OR (MH "Coping+") OR (MH "Adaptation, Psychological+")  OR TX resilience OR (MH "Hardiness")  OR TX "mental health literacy" OR (MH "Health Literacy")  OR TX "positive thinking" OR TX "positive thoughts" OR MH "Optimism"  OR TX "self-efficacy" OR (MH "Self-Efficacy") | 196,067 |
|  | #4 | #1 AND #2 AND #3 | 32,512 |
| ***Study design*** | #5 | TI randomiz* OR AB randomiz* OR TI randomis* OR AB randomis* OR TI trial OR AB trial OR TI experiment* OR AB experiment*  OR (MH "Experimental Studies+") OR PT ("controlled trial" or "randomized controlled trial" or "randomised controlled trial") | 848,974 |
|  | #6 | #4 AND #5 | 4,820 |
|  | #7 | #6 AND limiters - Published Date: 20030101-20231131, Chinese, English | 4,347 |

**Table 1-3 Search strings in Embase/Ovid**

| PICO framework |  | Search terms | Search hits |
| --- | --- | --- | --- |
| ***Population***  (Adolescent:10-19 years old following WHO definition) | #1 | adolescen*.ti,ab. or teen*.ti,ab. or youth*.ti,ab. or juvenile*.ti,ab. or (exp adolescent /) | 2,099,890 |
| ***Intervention***  (Any intervention aims to build capacity for adolescents from mental health literacy, resilience, self-efficacy, and positive thinking) | #2 | ('capacit*' or 'competenc*' or 'capabilit*' or 'skill development' or 'skill building' or 'strength*' or 'empower*').mp. or exp 'psychological resilience'/ or 'psychological resilience'.mp.  or 'self-efficacy'.mp.  or exp 'health literacy'/ or 'mental health literacy'.mp.  or 'positive thinking'.mp. or 'positive thoughts'.mp. or exp optimism/ or optimism.mp. | 2,442,040 |
| ***Outcome***  (Mental health literacy, positive thinking, resilience and self-efficacy) | #3 | exp 'psychological resilience'/ or 'psychological resilience'.mp.  or exp 'health literacy'/ or 'mental health literacy'.mp.  or 'positive thinking'.mp. or 'positive thoughts'.mp. or exp optimism/ or optimism.mp.  or 'self-efficacy'.mp. | 136,891 |
|  | #4 | #1 AND #2 AND #3 | 14,579 |
| ***Study design*** | #5 | (randomiz* or randomis* or trial or experiment*).ti,ab. or randomized controlled trial.mp. or exp randomized controlled trial/ or controlled trial.mp. or exp controlled study/ | 12,732,536 |
|  | #6 | #4 AND #5 | 5,336 |
|  | #7 | #6 AND limiters ((chinese or english) and yr="2003 - 2023") | 4,762 |

**Table 1-4 Search strings in APA PsycINFO/Ovid**

| PICO framework |  | Search terms | Search hits |
| --- | --- | --- | --- |
| ***Population***  (Adolescent:10-19 years old following WHO definition) | #1 | (adolescen* or 'teen*' or youth* or juvenile*).ti,ab. or adolescent.mp. | **596,573** |
| ***Intervention***  (Any intervention aims to build capacity for adolescents) | #2 | capacit*.mp. or (competenc*.mp. or (exp Competence/ or Competence.mp.)) or capabilit*.mp. or 'skill development'.mp. or 'skill building'.mp. or strength*.mp. or (empower*.mp. or (exp Empowerment/ or Empowerment.mp.)) or (exp "Resilience (Psychological)"/ or "Resilience (Psychological)".mp.) or (exp Mental Health Literacy/ or Mental Health Literacy.mp.) or (exp Self-Efficacy/ or Self-Efficacy.mp.) or (("positive thinking" or "positive thoughts").mp. or (exp Optimism/ or Optimism.mp.)) | 556,444 |
| ***Outcome***  (Mental health literacy, positive thinking, resilience and coping) | #3 | (exp Coping Behavior/ or Coping Behavior.mp.) or (exp "Resilience (Psychological)"/ or "Resilience (Psychological)".mp.) or (exp Mental Health Literacy/ or Mental Health Literacy.mp.) or (("positive thinking" or "positive thoughts").mp. or (exp Optimism/ or Optimism.mp.))  or (exp Self-Efficacy/ or Self-Efficacy.mp.) | 103,309 |
|  | #4 | #1 AND #2 AND #3 | 17,639 |
| ***Study design*** | #5 | (randomiz* or randomis* or trial or experiment*).ti,ab.or exp Clinical Trials/ or Clinical Trials.mp. or (exp Randomized Clinical Trials/ or Randomized Clinical Trials.mp.) | 670,247 |
|  | #6 | #4 AND #5 | 1,969 |
|  | #7 | limit #6 to ((chinese or english) and yr="2003 - 2023") | 1,732 |

**Table 1-5 Search strings in Web of Science**

| PICO framework |  | Search terms | Search hits |
| --- | --- | --- | --- |
| ***Population***  (Adolescent:10-19 years old following WHO definition) | #1 | (TI=(adolescen* OR teen*OR youth* OR juvenile* )) OR AB=(adolescen* OR teen*OR youth* OR juvenile*) | 664,761 |
| ***Intervention***  (Any intervention aims to build capacity for adolescents from mental health literacy, resilience, self-efficacy, and positive thinking) | #2 | TS=(capacit* OR competenc* OR capabilit*OR "skill development" OR "skill building" OR strength* OR empower* OR resilience OR "self-efficacy" OR "mental health literacy" OR "positive thinking" OR "positive thoughts" OR optimism) | 4, 741,042 |
| ***Outcome***  (Mental health literacy, positive thinking, resilience and coping) | #3 | TS=(coping* OR resilience OR "mental health literacy" OR "positive thinking" OR "positive thoughts" OR optimism OR "self-efficacy") | 431,267 |
|  | #4 | #1 AND #2 AND #3 | 15,312 |
| ***Study design*** | #5 | (TI=("randomized controlled trials" or "controlled trials")) OR AB=("randomized controlled trials" or "controlled trials") OR  TI=(randomiz* or randomis* or trial or experiment* ) OR AB=(randomiz* or randomis* or trial or experiment* ) | 9,474,857 |
|  | #6 | #4 AND #5 | 1,928 |
|  | #7 | Limit #6 and English, Chinese (Languages) and 2003 or 2004 or 2005 or 2006 or 2007 or 2008 or 2009 or 2010 or 2011 or 2012 or 2013 or 2014 or 2015 or 2016 or 2017 or 2018 or 2019 or 2020 or 2021 or 2022 or 2023 (Publication Years) | 1,710 |

Note: No article in Chinese were found

**Table 1-6 Search strings in Cochrane Library**

| PICO framework |  | Search terms | Search hits |
| --- | --- | --- | --- |
| ***Population***  (Adolescent:10-19 years old following WHO definition) | #1 | (adolescen* OR teen* OR youth*OR juvenile*):ti OR (adolescen* OR teen* OR youth*OR juvenile*):ab OR (adolescent [MeSH]) | 160,781 |
| ***Intervention***  (Any intervention aims to build capacity for adolescents from mental health literacy, resilience, self-efficacy, and positive thinking) | #2 | (capacit* OR competenc*OR capabilit* OR "skill development" OR "skill building" OR strength* OR empower*):ti,ab,kw  OR empowerment[MeSH])  OR (resilience:ti,ab,kw OR "Resilience, Psychological"[MeSH])  OR ("self efficacy":ti,ab,kw OR "Self Efficacy"[MeSH])  OR ("Health Literacy"[MeSH] OR "mental health literacy":ti,ab,kw)  OR ("positive thinking" OR "positive thoughts"):ti,ab,kw OR ("Optimism"[MeSH]) | 140,397 |
| ***Outcome***  (Mental health literacy, positive thinking, resilience and coping) | #3 | (coping*:ti,ab,kw OR "Adaptation, Psychological"[Mesh])  OR (resilience:ti,ab,kw OR "Resilience, Psychological"[MeSH])  OR ("Health Literacy"[MeSH] OR "mental health literacy":ti,ab,kw)  OR ("positive thinking" OR "positive thoughts"):ti,ab,kw OR ("Optimism"[MeSH]) OR ("self efficacy":ti,ab,kw OR "Self Efficacy"[MeSH]) | 24,524 |
|  | #4 | #1 AND #2 AND #3 | 3,439 |
| ***Study design*** | #5 | Randomized Controlled Trial [MeSH descriptor] OR Controlled Clinical Trial [MeSH descriptor] OR (randomis* or randomiz* or trial or experiment*):ti OR (randomis* or randomiz* or trial or experiment*):ab or ("randomized controlled trial" or "controlled trial"):pt | 1,3103,126 |
|  | #6 | #4 AND #5 | 2,583 |
|  | #7 | limit #6 with Publication Year from 2003 to 2023, in Trials, in English, Chinese | 2,352 |

**Table 1-7 Search strings in CNKI (Chinese National Knowledge Infrastructure)**

| PICO framework |  | Search terms | Search hits |
| --- | --- | --- | --- |
| ***Population***  (Adolescent:10-19 years old following WHO definition) | #1 | (TI = '青少年'+'青少年群体' OR AB = '青少年'+'青少年群体') | 642,833 |
| ***Intervention***  (Any intervention aims to build capacity for adolescents from mental health literacy, resilience, self-efficacy, and positive thinking) | #2 | SU %=('能力'+'技能培养'+'精神力量'+'韧性'+'心理韧性'+'赋权'+'自我效能'+'健康素养'+'心理健康素养'+'积极思维'+'积极心理'+'乐观') | 3,620,502 |
| ***Outcome***  (Mental health literacy, positive thinking, resilience and coping) | #3 | SU %= ('应对'+'韧性'+'心理韧性'+'健康素养'+'心理健康素养'+'积极思维'+'积极心理'+'乐观') | 217,004 |
| ***Study design*** | #4 | SU %= ('随机对照研究'+'随机对照实验'+'随机对照试验'+'随机对照'+'对照'+'干预'+实验') | 3,259,493 |
|  | #5 | #1 AND #2 AND #3 AND #4 | 430 |
|  | #6 | #5 AND 2003-2023, Chinese, English | 402 |

資源範圍:學術期刊；中英文擴展

(TI = '青少年'+'青少年群体' OR AB = '青少年'+'青少年群体') AND SU %=('能力'+'技能培养'+'精神力量'+'韧性'+'心理韧性'+'赋权'+'自我效能'+'健康素养'+'心理健康素养'+'积极思维'+'积极心理'+'乐观') AND SU %= ('韧性'+'心理韧性'+'健康素养'+'心理健康素养'+'积极思维'+'乐观') AND SU %= ('随机对照研究'+'随机对照实验'+'随机对照试验'+'随机对照'+'对照'+'干预'+'实验') =402

# **Supplementary Information 2 Effective sample size calculation （accounting for clustering in meta-analysis of cluster randomized controlled trials）**

**2.1 Berger 2018**

The cluster-randomized trial that randomized 6 classrooms (clusters) with 183 adolescents. At post-intervention, there were 95 adolescents in an experimental group and 88 adolescents in the control group. ICC not reported. Assume the intracluster correlation coefficient = 0.05.

The average cluster size is: (95+88)/(3+3)= 30.5. The design effect for the trial as a whole is 1 + (M – 1) ICC = 1 + (30.5 – 1) × 0.02 = 2.48.

The effective sample size in the experimental group is: 95/2.48=38.3= 38, and for the control group is: 88/2.48=35.4 =35

- 1. **Campos 2018**

The cluster-randomized trial that randomized 22 school classes of 543 adolescents. There were 11 school classes of 239 adolescents in experimental group and 11 school classes of 263 adolescents in a control group at post-intervention. With 211 adolescents and 176 adolescents at follow-up. ICC not reported. Assume the intracluster correlation coefficient = 0.05

The average cluster size is: 543/22= 25. The design effect for the trial as a whole is 1 + (M – 1) ICC = 1 + (25 – 1) × 0.05 =1.12

The effective sample size in the experimental group at post-intervention is: 239/1.12=213, and for the control group is:263/1.12=235

The effective sample size in the experimental group at follow-up is: 211/1.12=188, and for the control group is:176/1.12=157

- 1. **Felver 2019**

The cluster-randomized trial that randomized 2 classes (29 students). At post-intervention, there were 12 students in the experimental group and 11 students in the control group. Assume the intracluster correlation coefficient = 0.05

The average cluster size is: 29/2= 14.5. The design effect for the trial as a whole is 1 + (M – 1) ICC = 1 + (14.5 – 1) × 0.05 = 1.68.

The effective sample size in the experimental group is: 12/1.68=7, and for the control group is: 11/1.68= 7

- 1. **Green 2022**

The cluster-randomized trial that randomized 24 classrooms (clusters) with 396 adolescents. At post-intervention, there were 200 children (12 classes) in the experimental group and 172 adolescents (12 classes) in the control group. Assume the intracluster correlation coefficient = 0.05.

The average cluster size is: 396/24= 16.5. The design effect for the trial as a whole is 1 + (M – 1) ICC = 1 + (16.5 – 1) × 0.05 = 1.775

The effective sample size in the experimental group is: 200/1.772=113, and for the control group is: 172/1.775= 97.

- 1. **Johnstone 2020**

This pilot cluster-randomized trial randomized 5 schools (clusters) with 295 students into conditions. At post-intervention, there were 2 schools of 154 students in the experimental group (ER programme) and 1 school with 17 students in the control group. At follow-up, there were 2 schools of 74 students in the experimental group (ER programme) and 1 school with 18 students in the control group. Assume the intracluster correlation coefficient = 0.05.

The average cluster size is: 295/5= 59. The design effect for the trial as a whole is 1 + (M – 1) ICC = 1 + (59 – 1) × 0.05 = 3.9

At post-intervention, the effective sample size in the experimental group is: 154/3.9=39, and for the control group is: 17/3.9=4.

At follow-up, the effective sample size in the experimental group is: 74/3.9=19, and for the control group is: 18/3.9=4.

- 1. **Leventhal 2015**

This large cluster-randomized trial randomized 76 schools (clusters) with 2508 girls into conditions. At post-intervention, there were 1681 girls in the experimental group and 706 girls in the control group. Assume the intracluster correlation coefficient = 0.05.

The average cluster size is: 2508/76 = 33. The design effect for the trial as a whole is 1 + (M – 1) ICC = 1 + (33 – 1) × 0.05 = 2.6.

The effective sample size in the experimental group is: 1681/2.6=647, and for the control group is: 706/2.6=272.

- 1. **Liddle 2021**

This cluster-randomized trial randomized 9 teams (clusters) with 122 male adolescents into conditions. At post-intervention, with 47 male adolescents into the experimental group and 55 into the control group. Assume the intracluster correlation coefficient = 0.05.

The average cluster size is: 122/9= 14. The design effect for the trial as a whole is 1 + (M – 1) ICC = 1 + (14 – 1) × 0.05 = 1.65.

The effective sample size in the experimental group is: 47/1.65=29, and for the control group is: 55/1.65=33.

- 1. **MaalouF 2020**

This cluster-randomized trial randomized 10 schools (clusters) with 280 adolescents into conditions. At post-intervention, there were 5 schools with 102 adolescents into the experimental group and 5 schools with 126 into the control group. Assume the intracluster correlation coefficient = 0.05.

The average cluster size is: 280/10= 28. The design effect for the trial as a whole is 1 + (M – 1) ICC = 1 + (28 – 1) × 0.05 = 2.35

The effective sample size in the experimental group is: 102/2.35=43, and for the control group is: 126/2.35=54.

- 1. **O’Connor 2022**

This cluster-randomized trial randomized 29 schools (clusters) of 604 adolescents into conditions. At post-intervention, there were 15 schools of 210 adolescents into the experimental group and 14 schools of 305 adolescents into the control group at post-intervention. There were 15 169 adolescents in the experimental group and 237 adolescents in the control at the follow-up.

Assume the intracluster correlation coefficient = 0.05.

The average cluster size is: 604/29= 21. The design effect for the trial as a whole is 1 + (M – 1) ICC = 1 + (21 – 1) × 0.05 = 2.

At post-intervention, the effective sample size in the experimental group is: 210/2=105, and for the control group is: 305/2=153.

At follow-up, the effective sample size in the experimental group is: 169/2=85, and for the control group is: 237/2=119.

- 1. **Perry 2014**

This cRCT included 380 adolescents 22 classes (clusters) at baseline, with 207 adolescents in the experimental group, and 173 adolescents in the control group. At post-intervention, there were 160 adolescents in the experimental group and 162 adolescents in the control group. At follow-up, there were 138 adolescents in the experimental group and 70 adolescents in the control group. Assume the intracluster correlation coefficient = 0.05.

The average cluster size is: 380/22= 17. The design effect for the trial as a whole is 1 + (M – 1) ICC = 1 + (17 – 1) × 0.05 = 1.8

At post-intervention, the effective sample size in the experimental group is: 160/1.8=89, and for the control group is: 162/1.8=90.

At follow-up, the effective sample size in the experimental group is: 138/1.8=77, and for the control group is: 70/1.8=39.

- 1. **Seale 2022**

This pilot cluster-randomized trial randomized 30 classes (clusters) with 643 adolescents. At post-intervention, there were 15 classes of 299 adolescents in the experimental group and 15 schools of 312 adolescents in the control group. At follow-up, there were 241 in the experimental group and 277 in the control group. Assume the intracluster correlation coefficient = 0.05.

The average cluster size is: 643/30= 21. The design effect for the trial as a whole is 1 + (M – 1) ICC = 1 + (21– 1) × 0.05 = 2.

At post-intervention: The effective sample size in the experimental group is: 299/2=150, and for the control group is: 312/2=156.

At follow-up: The effective sample size in the experimental group is: 241/2=121, and for the control group is: 277/2=139.

- 1. **Tang 2022**

This cluster-randomized trial randomized 19 classes (clusters) of 706 adolescents into the experimental group and 24 classes of 850 adolescents into the control group at post-intervention. Authors reported ICC=0.034

The design effect for the trial as a whole is 1 + (M – 1) ICC = 1 + (36– 1) × 0.034 = 2.19

The effective sample size in the experimental group is: 706/2.19=322, and for the control group is: 850/2.19=388.

- 1. **Zare 2021**

This cluster-randomized trial randomized 8 schools (clusters) of 220 adolescent girl into conditions. There were 4 schools with 110 adolescents in the experimental group and 4 schools with 110 adolescents into the control group, at baseline and post-intervention. Assume the intracluster correlation coefficient = 0.05

The average cluster size is: 220/8= 28. The design effect for the trial as a whole is 1 + (M – 1) ICC = 1 + (28– 1) × 0.05 = 2.35

The effective sample size for both groups is: 110/2.35 =47

# **Supplementary Information 3 The overall risk of bias for all included studies**


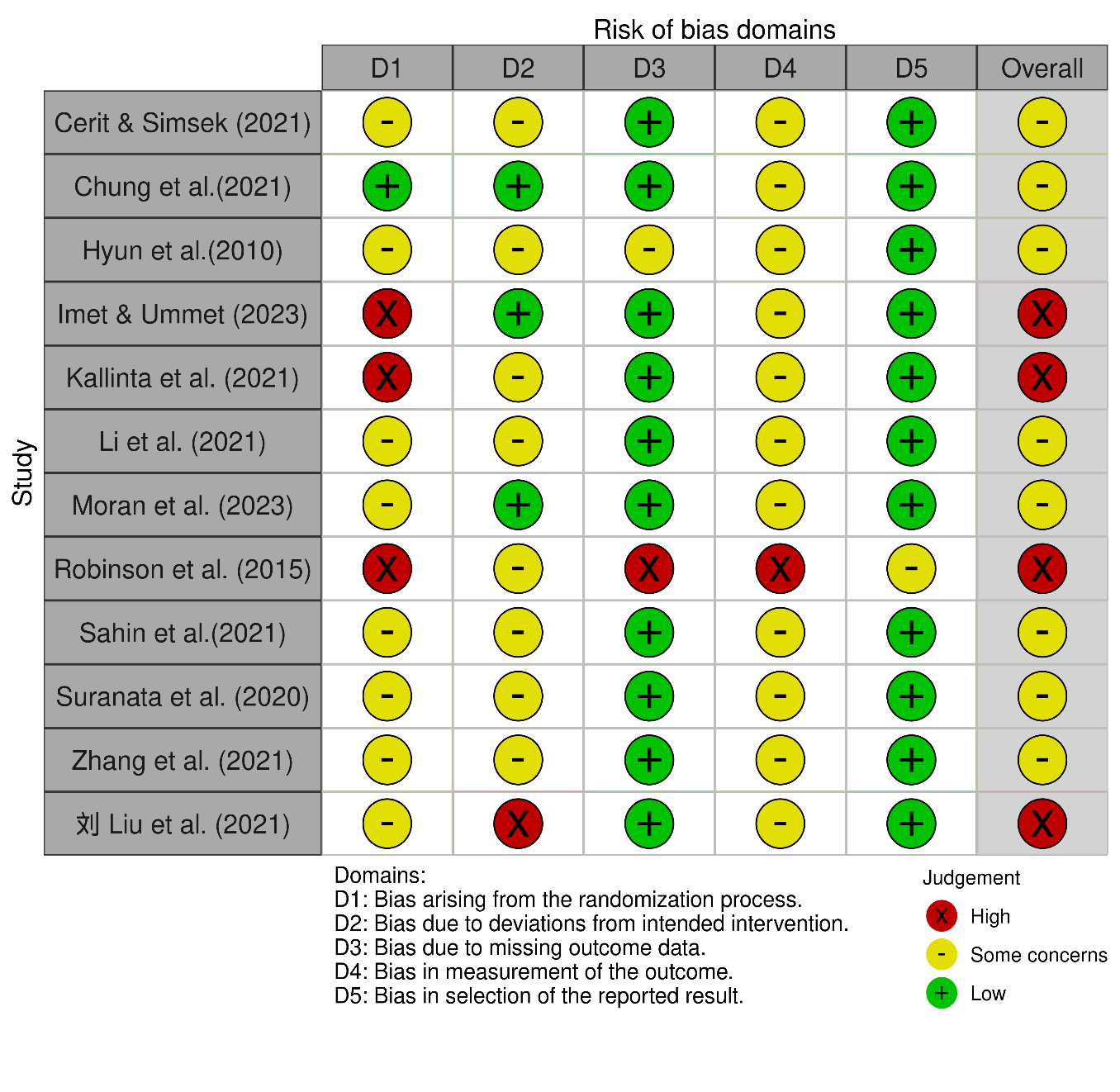


Figure 1-1 Cochrane risk of bias assessment for RCTs (*n*=12)


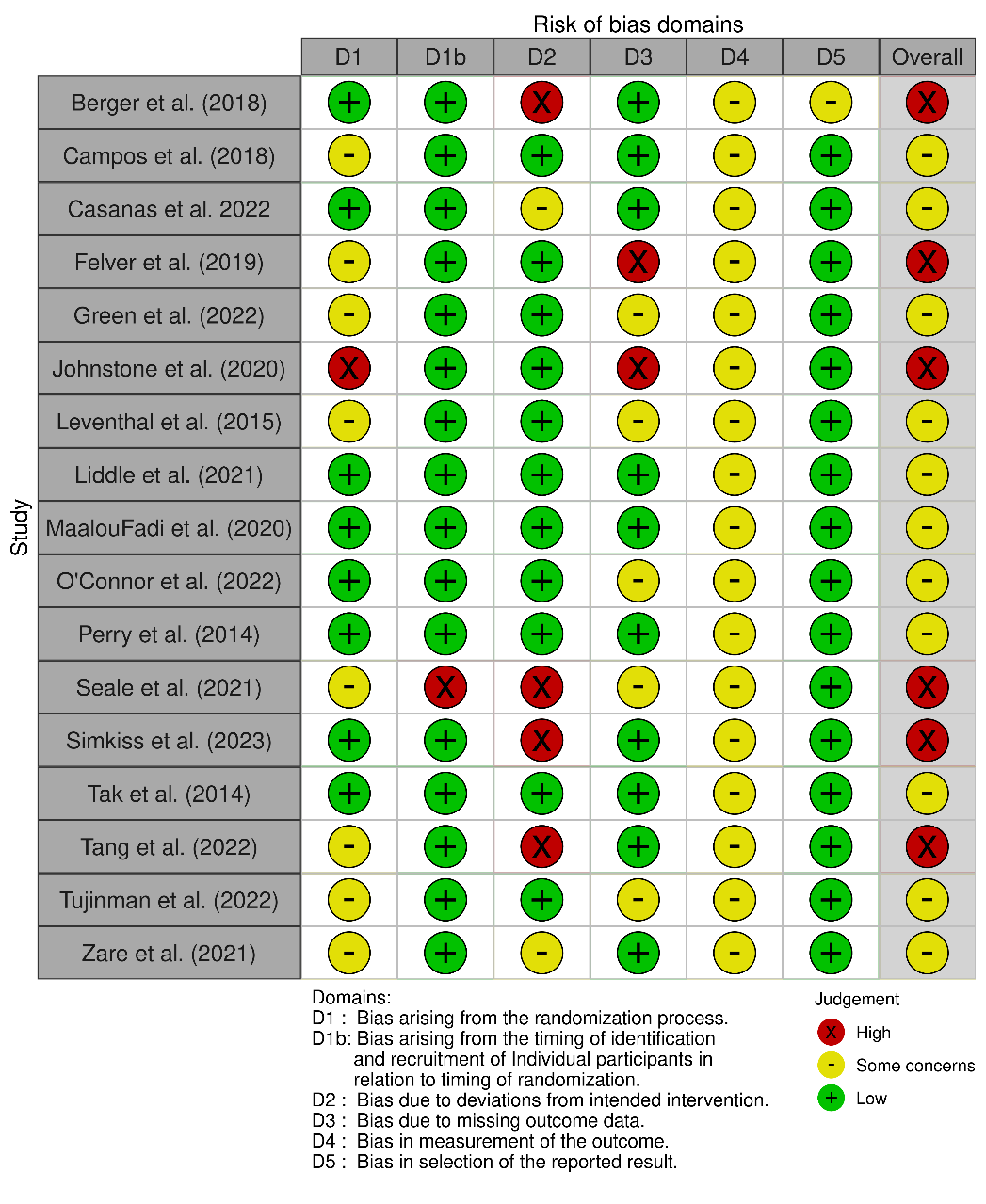


Figure 1-2 Cochrane risk of bias assessment for cluster [1-6]RCTs (*n*=17)


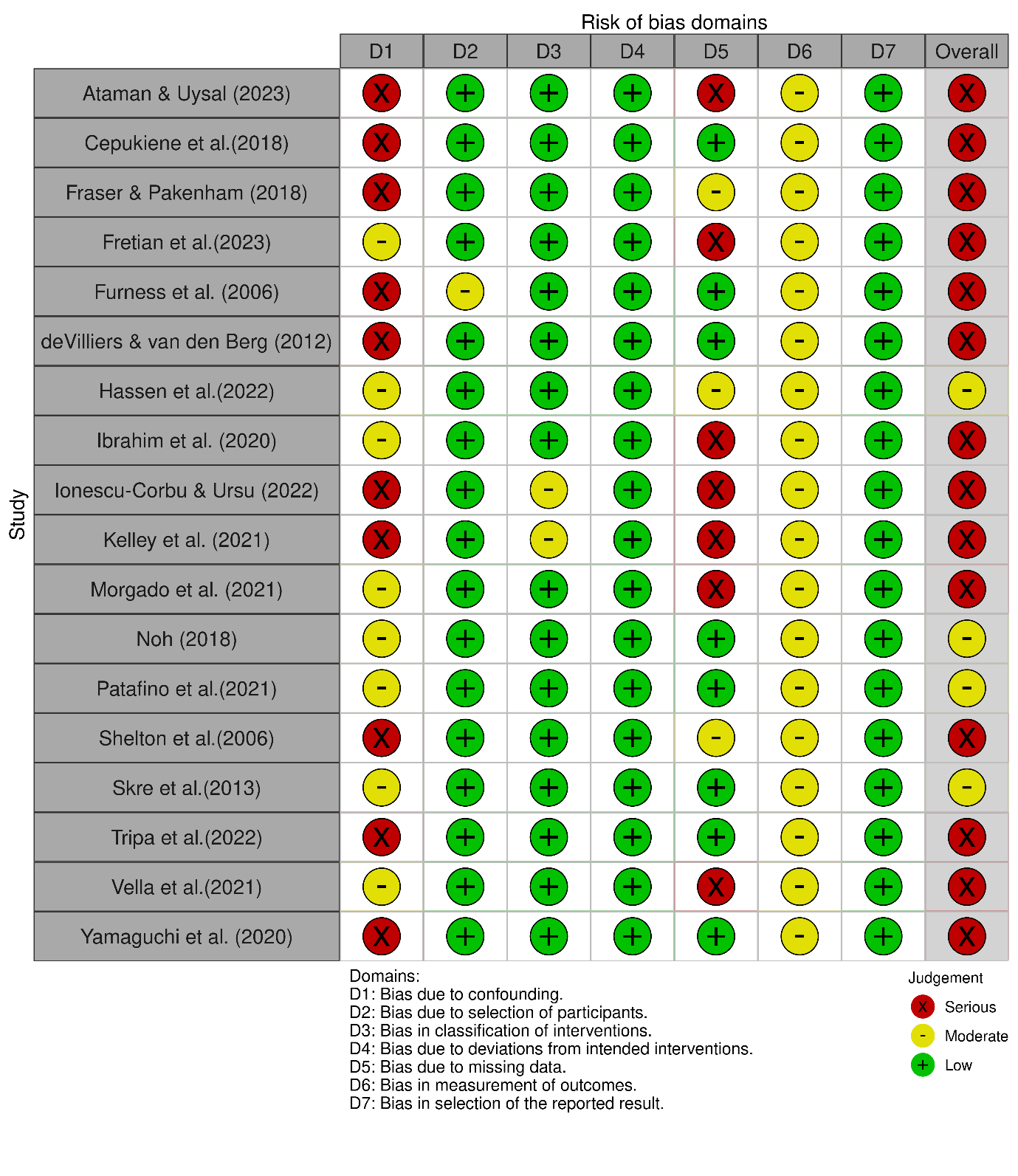


Figure 1-3 Cochrane risk of bias assessment for quasi-experimental and non-randomized experimental studies (*n*=18)

# **Supplementary Information 4 The risk of bias for each study**

**Table 2-1 Risk of Bias Assessment ROB2 Tool for RCTs**

| **Domains** | **Signalling questions** | Cerit & Simsek (2021) | Chung et al. (2021) | Hyun et al. (2010) | Imet & Ummet (2023) | Kallinta et al. (2021)I | Li et al. (2021) | Moran et al. (2023) | Robinson et al. (2015) | Sahin et al. (2021) | Suranata et al. (2020) | Zhang et al. (2021) | Liu et al. (2021) |
| --- | --- | --- | --- | --- | --- | --- | --- | --- | --- | --- | --- | --- | --- |
| **1.Randomization process:** | **1.1 Was the allocation sequence random?** | NI | Y | NI | PY | Y | PY | Y | NI | PY | PY | NI | NI |
|  | **1.2 Was the allocation sequence concealed until participants were enrolled and assigned to interventions?** | NI | PY | NI | NI | NI | NI | NI | NI | NI | NI | NI | NI |
|  | **1.3 Did baseline differences between intervention groups suggest a problem with the randomization process?** | NI | PN | PN | NI | PY | PN | NI | PY | NI | NI | PN | NI |
|  | **Overall risk of bias** | Some concerns | Low | Some concerns | High | High | Some concerns | Some concerns | High | Some concerns | Some concerns | Some concerns | Some concerns |
| **2. Deviation from Intended intervention:**  **(Intention-to treat-)** | **2.1. Were participants aware of their assigned intervention during the trial?** | PY | PY | PY | PY | PY | PY | PY | PY | NI | NI | PY | PY |
|  | **2.2. Were carers and people delivering the interventions aware of participants' assigned intervention during the trial?** | PY | PY | PY | PY | PY | PY | NI | PY | NI | PY | PY | PY |
|  | **2.3. If Y/PY/NI to 2.1 or 2.2: Were there deviations from the intended intervention that arose because of the trial context?** | PN | PN | NI | NI | NI | NI | PN | NI | NI | NI | NI | PN |
|  | **2.4 If Y/PY to 2.3: Were these deviations likely to have affected the outcome?** | NA | NA | NA | NA | NA | NA | NA | NA | NA | NA | NA | NA |
|  | **2.5. If Y/PY/NI to 2.4: Were these deviations from intended intervention balanced between groups?** | NA | NA | NA | NA | NA | NA | NA | NA | NA | NA | NA | NA |

**Table 2-1 (Continued)**

| **Domains** | **Signalling questions** | Cerit & Simsek (2021) | Chung et al. (2021) | Hyun et al. (2010) | Imet & Ummet (2023) | Kallinta et al. (2021)I | Li et al. (2021) | Moran et al. (2023) | Robinson et al. (2015) | Sahin et al. (2021) | Suranata et al. (2020) | Zhang et al. (2021) | Liu et al. (2021) |
| --- | --- | --- | --- | --- | --- | --- | --- | --- | --- | --- | --- | --- | --- |
| **2. Deviation from Intended intervention:**  **(Intention-to treat-)** | **2.6 Was an appropriate analysis used to estimate the effect of assignment to intervention?** | PY | PY | PY | PY | PY | PY | Y | PY | PY | Y | PY | PN |
|  | **2.7 If N/PN/NI to 2.6: Was there potential for a substantial impact (on the result) of the failure to analyse participants in the group to which they were randomized?** | NA | NA | NA | NA | NA | NA | NA | NA | NA | NA | NA | NA |
|  | **Risk-of-bias judgement** | Low | Low | Some concerns | Low | Some concerns | Some concerns | Low | Some concerns | Some concerns | Some concerns | Some concerns | High |
| **3.Missing outcome data** | **3.1 Were data for this outcome available for all, or nearly all, participants randomized?** | PN | Y | PN | PY | PN | PY | Y | NI | PY | Y | PN | PN |
|  | **3.2 If N/PN/NI to 3.1: Is there evidence that the result was not biased by missing outcome data?** | PN | NA | PN | NA | PN | NA | NA | PN | NA | NA | PN | PN |
|  | **3.3 If N/PN to 3.2: Could missingness in the outcome depend on its true value?** | PN | NA | PY | NA | PN | NA | NA | NI | NA | NA | PN | PN |
|  | **3.4 If Y/PY/NI to 3.3: Is it likely that missingness in the outcome depended on its true value?** | NA | NA | PN | NA | NA | NA | NA | NI | NA | NA | NA | NA |
|  | **Risk-of-bias judgement** | Low | Low | Some concerns | Low | Low | Low | Low | High | Low | Low | Low | Low |
| **4.Measurement of Outcome** | **4.1 Was the method of measuring the outcome inappropriate?** | PN | PN | PN | PN | PN | PN | PN | PN | PN | PN | PN | PN |

**Table 2-1 (Continued)**

| **Domains** | **Signalling questions** | Cerit & Simsek (2021) | Chung et al. (2021) | Hyun et al. (2010) | Imet & Ummet (2023) | Kallinta et al. (2021)I | Li et al. (2021) | Moran et al. (2023) | Robinson et al. (2015) | Sahin et al. (2021) | Suranata et al. (2020) | Zhang et al. (2021) | Liu et al. (2021) |
| --- | --- | --- | --- | --- | --- | --- | --- | --- | --- | --- | --- | --- | --- |
| **4.Measurement of Outcome** | **4.2 Could measurement or ascertainment of the outcome have differed between intervention groups?** | PN | PN | PN | PN | PN | PN | PN | PN | PN | PN | PN | PN |
|  | **4.3 If N/PN/NI to 4.1 and 4.2: Were outcome assessors aware of the intervention received by study participants?** | PY | PY | PY | PY | PY | PY | PY | PY | PY | PY | PY | PY |
|  | **4.4 If Y/PY/NI to 4.3: Could assessment of the outcome have been influenced by knowledge of intervention received?** | PY | PY | PY | PY | PY | PY | PY | NI | PY | PY | PY | PY |
|  | **4.5 If Y/PY/NI to 4.4: Is it likely that assessment of the outcome was influenced by knowledge of intervention received?** | PN | PN | PN | PN | PN | PN | PN | N | PN | PN | PN | PN |
|  | **Risk-of-bias judgement** | Some concerns | Some concerns | Some concerns | Some concerns | Some concerns | Some concerns | Some concerns | High | Some concerns | Some concerns | Some concerns | Some concerns |
| **5. Selection of reported result** | **5.1 Were the data that produced this result analysed in accordance with a pre-specified analysis plan that was finalized before unblinded outcome data were available for analysis?**  **Is the numerical result being assessed likely to have been selected, on the basis of the results, from.** | Y | Y | Y | Y | Y | Y | Y | Y | PY | PY | Y | PY |

**Table 2-1 (Continued)**

| **Domains** | **Signalling questions** | Cerit & Simsek (2021) | Chung et al. (2021) | Hyun et al. (2010) | Imet & Ummet (2023) | Kallinta et al. (2021)I | Li et al. (2021) | Moran et al. (2023) | Robinson et al. (2015) | Sahin et al. (2021) | Suranata et al. (2020) | Zhang et al. (2021) | Liu et al. (2021) |
| --- | --- | --- | --- | --- | --- | --- | --- | --- | --- | --- | --- | --- | --- |
| **5. Selection of reported result** | **5.2. ... multiple eligible outcome measurements (e.g. scales, definitions, time points) within the outcome domain?** | N | PN | PN | PN | PN | PN | PN | PN | PN | PN | PN | PN |
|  | **5.3 ... multiple eligible analyses of the data?** | N | PN | PN | PN | PN | PN | PN | NI | PN | PN | PN | PN |
|  | **Risk-of-bias judgement** | Low | Low | Low | Low | Low | Low | Low | Some concerns | Low | Low | Low | Low |
| **Overall risk of bias** | | Some concerns | Some concerns | Some concerns | High | High | Some concerns | Some concerns | High | Some concerns | Some concerns | Some concerns | High |

**Abbreviation：Y=yes; PY=probably yes; PN=probably no; N=no; NA=not applicable; NI=no information.**

**Table 2-2 ROB2 Tool for cRCTs**

| **Domains** | **Signalling questions** | Berger et al. (2018) | Campos et al. (2018) | Casanas et al. (2022) | Felver et al. (2019) | Green et al. (2022) | Johnstone et al. (2020) | Leventhal et al. (2015) | Liddle et al. (2021) | MaalouFadi et al. (2020) | O'Connor et al. (2022) | Perry et al. (2014) | Seale et al. (2021) | Simkiss et al. (2023) | Tak et al. (2014) | Tang et al. (2022) | Tujinman et al. (2022) | Zare et al. (2021) |
| --- | --- | --- | --- | --- | --- | --- | --- | --- | --- | --- | --- | --- | --- | --- | --- | --- | --- | --- |
| **1.Randomization process:** | **1a.1 Was the allocation sequence random?** | Y | PY | Y | Y | PY | Y | PY | Y | Y | Y | Y | PY | Y | Y | NI | PY | NI |
|  | **1a.2 Was the allocation sequence concealed until participants were enrolled and assigned to interventions?** | PY | NI | Y | NI | NI | N | NI | Y | PY | PY | Y | NI | PY | PY | NI | NI | NI |
|  | **1a.3 Did baseline differences between intervention groups suggest a problem with the randomization process?** | NI | PN | PN | PN | PN | PY | PN | PN | PN | PN | PN | NI | PN | PN | NI | PN | PN |
|  | **Overall risk of bias** | Low | Some conce-rns | Los | Some conce-rns | Some conce-rns | High | Some concerns | Low | Low | Low | Low | Some concerns | Low | Low | Some concerns | Some concerns | Some concerns |
|  | **1b.1 Were all the individual participants identified and recruited (if appropriate) before randomization of**  **clusters?** | Y | PY | Y | PY | PY | PY | PY | PY | Y | PY | PY | N | PY | PY | PY | PY | PY |
|  | **1b.2 If N/PN/NI to 1b.1: Is it likely that selection of individual participants was affected by knowledge of the**  **intervention assigned to the cluster?** | NA | NA | NA | NA | NA | NA | NA | NA | NA | NA | NA | PY | NA | NA | NA | NA | NA |
|  | **1b.3 Were there baseline imbalances that suggest differential identification**  **or recruitment of individual participants between intervention groups?** | PN | NI | PN | PN | PN | NI | PN | PN | PN | PN | PN | NI | PN | NI | NI | PN | PN |
|  | **Overall risk of bias** | Low | Low | Low | Low | Low | Low | Low | Low | Low | Low | Low | High | Low | Low | Low | Low | Low |

**Table 2-2 (Continued)**

| **Domains** | **Signalling questions** | Berger et al. (2018) | Campos et al. (2018) | Casanas et al. (2022) | Felver et al. (2019) | Green et al. (2022) | Johnstone et al. (2020) | Leventhal et al. (2015) | Liddle et al. (2021) | MaalouFadi et al. (2020) | O'Connor et al. (2022) | Perry et al. (2014) | Seale et al. (2021) | Simkiss et al. (2023) | Tak et al. (2014) | Tang et al. (2022) | Tujinman et al. (2022) | Zare et al. (2021) |
| --- | --- | --- | --- | --- | --- | --- | --- | --- | --- | --- | --- | --- | --- | --- | --- | --- | --- | --- |
| **2. deviation from intended intervention（intention-to treat）** | **2.1a. Were participants aware of their assigned intervention during the trial?** | NI | NI | NI | NI | NI | PY | NI | NI | NI | NI | NI | NI | NI | NI | NI | NI | NI |
|  | **2.1b. If Y/PY/NI to 2.1a: Were participants aware of their assigned intervention during the trial?** | NI | PY | PY | PY | PY | PY | PY | PY | PY | PN | PY | NI | Y | Y | PY | PY | PY |
|  | **2.2. Were carers and people delivering the interventions aware of participants' assigned intervention during the trial?** | PY | PY | PY | PY | PY | PY | PY | PY | PY | PN | PY | NI | Y | Y | PY | PY | PY |
|  | **2.3. If Y/PY/NI to 2.1 or 2.2: Were there deviations from the intended intervention that arose because of the trial context?** | PN | PN | NI | PN | PN | PN | PN | PN | PN | PN | PN | PY | NI | PN | PN | PN | NI |
|  | **2.4 If Y/PY to 2.3: Were these deviations likely to have affected the outcome?** | NA | NA | NA | NA | NA | NA | NA | NA | NA | NA | NA | NI | NA | NA | NA | NA | NA |
|  | **2.5. If Y/PY/NI to 2.4: Were these deviations from intended intervention balanced between groups?** | NA | NA | NA | NA | NA | NA | NA | NA | NA | NA | NA | PY | NA | NA | NA | NA | NA |
|  | **2.6 Was an appropriate analysis used to estimate the effect of assignment to intervention?** | PN | PY | PY | PY | PY | PY | PY | PY | PY | Y | Y | PN | PN | Y | PN | PY | PY |

**Table 2-2 (Continued)**

| **Domains** | **Signalling questions** | Berger et al. (2018) | Campos et al. (2018) | Casanas et al. (2022) | Felver et al. (2019) | Green et al. (2022) | Johnstone et al. (2020) | Leventhal et al. (2015) | Liddle et al. (2021) | MaalouFadi et al. (2020) | O'Connor et al. (2022) | Perry et al. (2014) | Seale et al. (2021) | Simkiss et al. (2023) | Tak et al. (2014) | Tang et al. (2022) | Tujinman et al. (2022) | Zare et al. (2021) |
| --- | --- | --- | --- | --- | --- | --- | --- | --- | --- | --- | --- | --- | --- | --- | --- | --- | --- | --- |
| **2. deviation from intended intervention（intention-to treat）** | **2.7 If N/PN/NI to 2.6: Was there potential for a substantial impact (on the result) of the failure to analyse participants in the group to which they were randomized?** | NI | NA | NA | NA | NA | NA | NA | NA | NA | NA | NA | NI | NI | NA | NI | NA | NA |
|  | **Risk-of-bias judgement** | High | Low | Some conce-rns | Low | Low | Low | Low | Low | Low | Low | Low | High | High | Low | High | Low | Some conce-rns |
| **3.Missing outcome data** | **3.1a Were data for this outcome available for all, or nearly all, participants randomized?** | Y | Y | PN | PY | PY | Y | Y | PY | Y | Y | Y | Y | Y | PY | Y | Y | Y |
|  | **3.1b Were data for this outcome available for all, or nearly all,**  **participants within clusters?** | PN | PN | PN | PN | PN | PN | PN | PN | PN | PN | PN | PN | PY | PN | PN | PN | PY |
|  | **3.2 If N/PN/NI to 3.1: Is there evidence that the result was not biased by missing outcome data?** | PN | PN | PN | PN | PN | PN | PN | PN | PY | PN | PN | PN | NA | PN | PN | PN | NA |
|  | **3.3 If N/PN to 3.2: Could missingness in the outcome depend on its true value?** | PN | PN | PN | PY | PY | PY | PY | PN | NA | PY | PN | PY | NA | PN | PN | PY | NA |
|  | **3.4 If Y/PY/NI to 3.3: Is it likely that missingness in the outcome depended on its true value?** | NA | NA | NA | PY | PN | PY | PN | NA | NA | PN | NA | PN | NA | NA | NA | PN | NA |
|  | **Risk-of-bias judgement** | Low | Low | Low | High | Some conce-rns | High | Some conce-rns | Low | Low | Some conce-rns | Low | Some conce-rns | Low | Low | Low | Some conce-rns | Low |

**Table 2-2 (Continued)**

| **Domains** | **Signalling questions** | Berger et al. (2018) | Campos et al. (2018) | Casanas et al. (2022) | Felver et al. (2019) | Green et al. (2022) | Johnstone et al. (2020) | Leventhal et al. (2015) | Liddle et al. (2021) | MaalouFadi et al. (2020) | O'Connor et al. (2022) | Perry et al. (2014) | Seale et al. (2021) | Simkiss et al. (2023) | Tak et al. (2014) | Tang et al. (2022) | Tujinman et al. (2022) | Zare et al. (2021) |
| --- | --- | --- | --- | --- | --- | --- | --- | --- | --- | --- | --- | --- | --- | --- | --- | --- | --- | --- |
| **4.Measurement of Outcome** | **4.1 Was the method of measuring the outcome inappropriate?** | PN | PN | PN | PN | PN | PN | PN | PN | PN | PN | PN | PN | PN | PN | PN | PN | PN |
|  | **4.2 Could measurement or ascertainment of the outcome have differed between intervention groups?** | PN | PN | PN | PN | PN | PN | PN | PN | PN | PN | PN | PN | PN | PN | PN | PN | PN |
|  | **4.3a If N/PN/NI to 4.1 and 4.2: Were**  **outcome assessors aware that a trial**  **was taking place?** | NI | NI | NI | NI | NI | PY | NI | NI | NI | NI | NI | PY | NI | NI | NI | NI | NI |
|  | **4.3b If Y/PY/NI to 4.3a: Were outcome**  **assessors aware of the intervention**  **received by study participants?** | PY | PY | PY | PY | PY | PY | PY | PY | PY | PY | PY | PY | PY | PY | PY | PY | PY |
|  | **4.4 If Y/PY/NI to 4.3: Could assessment of the outcome have been influenced by knowledge of intervention received?** | PY | PY | PY | PY | PY | PY | PY | PY | PY | PY | PY | PY | PY | PY | PY | PY | PY |
|  | **4.5 If Y/PY/NI to 4.4: Is it likely that assessment of the outcome was influenced by knowledge of intervention received?** | NI | PN | PN | PN | PN | PN | PN | PN | PN | PN | PN | PN | PN | PN | PN | PN | PN |
|  | **Risk-of-bias judgement** | Some concerns | Some concerns | Some concerns | Some concerns | Some concerns | Some concerns | Some concerns | Some concerns | Some concerns | Some concerns | Some concerns | Some concerns | Some concerns | Some concerns | Some concerns | Some concerns | Some concerns |

**Table 2-2 (Continued)**

| **Domains** | **Signalling questions** | Berger et al. (2018) | Campos et al. (2018) | Casanas et al. (2022) | Felver et al. (2019) | Green et al. (2022) | Johnstone et al. (2020) | Leventhal et al. (2015) | Liddle et al. (2021) | MaalouFadi et al. (2020) | O'Connor et al. (2022) | Perry et al. (2014) | Seale et al. (2021) | Simkiss et al. (2023) | Tak et al. (2014) | Tang et al. (2022) | Tujinman et al. (2022) | Zare et al. (2021) |
| --- | --- | --- | --- | --- | --- | --- | --- | --- | --- | --- | --- | --- | --- | --- | --- | --- | --- | --- |
| **5. Selection of reported result** | **5.1 Were the data that produced this result analysed in accordance with a pre-specified analysis plan that was finalized before unblinded outcome data were available for analysis?**  **Is the numerical result being assessed likely to have been selected, on the basis of the results, from.** | PY | PY | Y | Y | PY | Y | PY | PY | Y | Y | Y | Y | PY | Y | Y | PY | Y |
|  | **5.2. ... multiple eligible outcome measurements (e.g. scales, definitions, time points) within the outcome domain?** | PN | PN | PN | PN | PN | PN | PN | PN | PN | PN | PN | PN | PN | PN | PN | PN | PN |
|  | **5.3 ... multiple eligible analyses of the data?** | PN | PN | PN | PN | PN | PN | PN | PN | PN | PN | PN | PN | PN | PN | PN | PN | PN |
|  | **Risk-of-bias judgement** | Low | Low | Low | Low | Low | Low | Low | Low | Low | Low | Low | Low | Low | Low | Low | Low | Low |
| **Overall risk of bias** | | High | Some concerns | Some concerns | High | Some concerns | High | Some concerns | Some concerns | Some concerns | Some concerns | Some concerns | High | High | Some concerns | High | Some concerns | Some concerns |

**Table 2-3 ROBIN-I Tool for Non-randomized Studies of Interventions**

| **Domains** | **Signalling questions** | Ataman & Uysal (2023) | deVilliers & van den Berg (2012) | Cepukiene et al.(2018) | Fraser & Pakenham (2008) | Fretian et al. (2023) | Furness et al. (2017) | Hassen et al. (2022) | Ibrahim et al. (2020) | Ionescu-Corbu & Ursu (2022) | Kelley et al. (2021) | Morgado et al. (2021) | Noh (2018) | Patafino et al. (2021) | Shelton et al. (2006) | Skre et al. (2013) | Tripa et al. (2022) | Vella et al. (2021) | Yamaguchi et al. (2020) |
| --- | --- | --- | --- | --- | --- | --- | --- | --- | --- | --- | --- | --- | --- | --- | --- | --- | --- | --- | --- |
| **Bias due to confounding** | **1.1 Is there potential for confounding of the effect of intervention in this study? If N/PN to 1.1: the study can be considered to be at low risk of bias due to confounding and no further signalling questions need be considered** | PY | PY | PY | PY | PY | PY | PY | PY | PY | PY | PY | PY | PY | PY | Y | PY | PY | PY |
|  | **If Y/PY to 1.1: determine whether there is a need to assess time-varying confounding** | / | / | / | / | / | / | / | / | / | / | / | / | / | / | / | / | / | / |
|  | **1.2. Was the analysis based on splitting participants’ follow up time according to interventionreceived?**  **If N/PN, answer questions relating to baseline confounding (1.4 to 1.6) If Y/PY, go to question 1.3.** | NA | NA | NA | NA | NA | NA | NA | NA | NA | NA | NA | NA | NA | NA | NA | NA | NA | NA |
|  | **1.3. Were intervention discontinuations or switches likely to be related to factors that are prognostic for the outcome? If N/PN, answer questions relating to baseline confounding (1.4 to 1.6) If Y/PY, answer questions relating to both baseline and time-varying confounding (1.7 and 1.8)** | NA | NA | NA | NA | NA | NA | NA | NA | NA | NA | NA | NA | NA | NA | NA | NA | NA | NA |

**Table 2-3 (Continued)**

| **Domains** | **Signalling questions** | Ataman & Uysal (2023) | deVilliers & van den Berg (2012) | Cepukiene et al.(2018) | Fraser & Pakenham (2008) | Fretian et al. (2023) | Furness et al. (2017) | Hassen et al. (2022) | Ibrahim et al. (2020) | Ionescu-Corbu & Ursu (2022) | Kelley et al. (2021) | Morgado et al. (2021) | Noh (2018) | Patafino et al. (2021) | Shelton et al. (2006) | Skre et al. (2013) | Tripa et al. (2022) | Vella et al. (2021) | Yamaguchi et al. (2020) |
| --- | --- | --- | --- | --- | --- | --- | --- | --- | --- | --- | --- | --- | --- | --- | --- | --- | --- | --- | --- |
| **Bias due to confounding** | **Questions relating to baseline confounding only** | | | | | | | | | | | | | | | | | | |
|  | **1.4. Did the authors use an appropriate analysis method that controlled for all the important confounding domains?** | PN | N | N | PN | PN | N | Y | PY | N | N | PN | PY | PY | N | PY | N | PY | N |
|  | **1.5. If Y/PY to 1.4: Were confounding domains that were controlled for measured validly and reliably by the variables available in this study?** | NA | NA | NA | NA | NA | NA | NA | NA | NA | NA | NA | PY | NA | NA | NA | NA | PY | NA |
|  | **1.6. Did the authors control for any post-intervention variables that could have been affected by the intervention?** | NI | NI | NI | NI | NI | NI | NI | NI | NI | NI | NI | NI | NI | NI | NI | NI | NI | NI |
|  | **Questions relating to baseline and time-varying confounding** | | | | | | | | | | | | | | | | | | |
|  | **1.7. Did the authors use an appropriate analysis method that controlled for all the important confounding domains and for time-varying confounding?** | NA | NA | NA | NA | NA | NA | NA | NA | NA | NA | NA | NA | NA | NA | NA | NA | NA | NA |
|  | **1.8. If Y/PY to 1.7: Were confounding domains that were controlled for measured validly and reliably by the variables available in this study?** | NA | NA | NA | NA | NA | NA | NA | NA | NA | NA | NA | NA | NA | NA | NA | NA | NA | NA |
|  | **Risk of bias judgement** | Serious | Serious | Serious | Mod | Mod | Serious | Mod | Mod | Serious | Serious | Mod | Mod | Mod | Serious | Mod | Serious | Mod | Serious |

**Table 2-3 (Continued)**

| **Domains** | **Signalling questions** | Ataman & Uysal (2023) | deVilliers & van den Berg (2012) | Cepukiene et al.(2018) | Fraser & Pakenham (2008) | Fretian et al. (2023) | Furness et al. (2017) | Hassen et al. (2022) | Ibrahim et al. (2020) | Ionescu-Corbu & Ursu (2022) | Kelley et al. (2021) | Morgado et al. (2021) | Noh (2018) | Patafino et al. (2021) | Shelton et al. (2006) | Skre et al. (2013) | Tripa et al. (2022) | Vella et al. (2021) | Yamaguchi et al. (2020) |
| --- | --- | --- | --- | --- | --- | --- | --- | --- | --- | --- | --- | --- | --- | --- | --- | --- | --- | --- | --- |
| **Bias in selection of participants into the study** | **2.1. Was selection of participants into the study (or into the analysis) based on participant characteristics observed after the start of intervention?**  **If N/PN to 2.1: go to 2.4** | PN | PN | PN | PN | PN | PN | PN | PN | PN | PN | PN | PN | PN | PN | PN | PN | PN | PN |
|  | **2.2. If Y/PY to 2.1: Were the post-intervention variables that influenced selection likely to be associated with intervention?** | NA | NA | NA | NA | NA | NA | NA | NA | NA | NA | NA | NA | NA | NA | NA | NA | NA | NA |
|  | **2.3 If Y/PY to 2.2: Were the post-intervention variables that influenced selection likely to be influenced by the outcome or a cause of the outcome?** | NA | NA | NA | NA | NA | NA | NA | NA | NA | NA | NA | NA | NA | NA | NA | NA | NA | NA |
|  | **2.4. Do start of follow-up and start of intervention coincide for most participants?** | Y | Y | Y | Y | PY | N | Y | Y | Y | PY | PY | Y | Y | Y | Y | Y | Y | Y |
|  | **2.5. If Y/PY to 2.2 and 2.3, or N/PN to 2.4: Were adjustment techniques used that are likely to correct for the presence of selection biases?** | NA | NA | NA | NA | NA | N | NA | NA | NA | NA | NA | NA | NA | NA | NA | NA | NA | NA |
|  | **Risk of bias judgement** | Low | Low | Low | Low | Low | Mod | Low | Low | Low | Low | Low | Low | Low | Low | Low | Low | Low | Low |

**Table 2-3 (Continued)**

| **Domains** | **Signalling questions** | Ataman & Uysal (2023) | deVilliers & van den Berg (2012) | Cepukiene et al.(2018) | Fraser & Pakenham (2008) | Fretian et al. (2023) | Furness et al. (2017) | Hassen et al. (2022) | Ibrahim et al. (2020) | Ionescu-Corbu & Ursu (2022) | Kelley et al. (2021) | Morgado et al. (2021) | Noh (2018) | Patafino et al. (2021) | Shelton et al. (2006) | Skre et al. (2013) | Tripa et al. (2022) | Vella et al. (2021) | Yamaguchi et al. (2020) |
| --- | --- | --- | --- | --- | --- | --- | --- | --- | --- | --- | --- | --- | --- | --- | --- | --- | --- | --- | --- |
| **Bias in classification of interventions** | **3.1 Were intervention groups clearly defined?** | Y | Y | Y | Y | Y | Y | Y | Y | Y | Y | Y | Y | Y | Y | Y | Y | Y | Y |
|  | **3.2 Was the information used to define intervention groups recorded at the start of the intervention?** | Y | Y | Y | Y | Y | Y | Y | Y | PN | Y | Y | Y | Y | Y | Y | Y | Y | Y |
|  | **3.3 Could classification of intervention status have been affected by knowledge of the outcome or risk of the outcome?** | NI | NI | NI | NI | NI | NI | NI | NI | NI | PY | NI | NI | NI | NI | NI | NI | NI | NI |
|  | **Risk of bias judgement** | Low | Low | Low | Low | Low | Low | Low | Low | Mod | Mod | Low | Low | Low | Low | Low | Low | Low | Low |
| **Bias due to deviations from intended interventions** | **4.1. Were there deviations from the intended intervention beyond what would be expected in usual practice?** | PN | PN | PN | PN | PN | PN | PN | PN | PN | PN | PN | PN | PN | PN | PN | PN | PN | PN |
|  | **4.2. If Y/PY to 4.1: Were these deviations from intended intervention unbalanced between groups *and* likely to have affected the outcome?** | NA | NA | NA | NI | NA | NA | NA | NA | NA | NA | NA | NA | NA | NA | NA | NA | NA | NA |
|  | **Risk of bias judgement** | Low | Low | Low | Low | Low | Low | Low | Low | Low | Low | Low | Low | Low | Low | Low | Low | Low | Low |

**Table 2-3 (Continued)**

| **Domains** | **Signalling questions** | Ataman & Uysal (2023) | deVilliers & van den Berg (2012) | Cepukiene et al.(2018) | Fraser & Pakenham (2008) | Fretian et al. (2023) | Furness et al. (2017) | Hassen et al. (2022) | Ibrahim et al. (2020) | Ionescu-Corbu & Ursu (2022) | Kelley et al. (2021) | Morgado et al. (2021) | Noh (2018) | Patafino et al. (2021) | Shelton et al. (2006) | Skre et al. (2013) | Tripa et al. (2022) | Vella et al. (2021) | Yamaguchi et al. (2020) |
| --- | --- | --- | --- | --- | --- | --- | --- | --- | --- | --- | --- | --- | --- | --- | --- | --- | --- | --- | --- |
| **Bias due to missing data** | **5.1 Were outcome data available for all, or nearly all, participants?** | PN | PY | Y | PN | PN | PY | PN | PN | N | PN | PN | Y | PY | NI | PY | PY | PN | PY |
|  | **5.2 Were participants excluded due to missing data on intervention status?** | N | PN | N | PN | PN | PN | PN | N | N | NI | PN | N | N | NI | NI | N | NI | N |
|  | **5.3 Were participants excluded due to missing data on other variables needed for the analysis?** | PY | PN | N | PY | PY | PN | PY | PY | PN | NI | PY | N | N | NI | NI | N | NI | N |
|  | **5.4 If PN/N to 5.1, or Y/PY to 5.2 or 5.3: Are the proportion of participants and reasons for missing data similar across interventions?** | NI | NA | NA | PY | NI | NA | NI | NI | NA | NI | PY | NA | Y | NI | NI | NA | NI | PY |
|  | **5.5 If PN/N to 5.1, or Y/PY to 5.2 or 5.3: Is there evidence that results were robust to the presence of missing data?** | PN | NA | NA | PN | PN | NA | NI | PN | NA | PN | PN | NA | NA | NI | PY | NA | PN | PY |
|  | **Risk of bias judgement** | Serious | Low | Low | Mod | Serious | Low | Mod | Serious | Serious | Serious | Serious | Low | Low | NI/Serio | Low | Low | Serious | Low |
| **Bias in measurement of outcomes** | **6.1 Could the outcome measure have been influenced by knowledge of the intervention received?** | PY | PY | PY | PY | PY | PY | PY | PY | PY | PY | PY | PY | PY | PY | PY | PY | PY | PY |
|  | **6.2 Were outcome assessors aware of the intervention received by study participants?** | PY | Y | Y | PY | Y | PY | Y | PY | PY | Y | PY | PY | Y | PY | PY | PY | PY | Y |

**Table 2-3 (Continued)**

| **Domains** | **Signalling questions** | Ataman & Uysal (2023) | deVilliers & van den Berg (2012) | Cepukiene et al.(2018) | Fraser & Pakenham (2008) | Fretian et al. (2023) | Furness et al. (2017) | Hassen et al. (2022) | Ibrahim et al. (2020) | Ionescu-Corbu & Ursu (2022) | Kelley et al. (2021) | Morgado et al. (2021) | Noh (2018) | Patafino et al. (2021) | Shelton et al. (2006) | Skre et al. (2013) | Tripa et al. (2022) | Vella et al. (2021) | Yamaguchi et al. (2020) |
| --- | --- | --- | --- | --- | --- | --- | --- | --- | --- | --- | --- | --- | --- | --- | --- | --- | --- | --- | --- |
| **Bias in measurement of outcomes** | **6.3 Were the methods of outcome assessment comparable across intervention groups?** | PY | PY | PY | PY | Y | PY | PY | N | PY | PY | PY | PY | PY | PY | PY | PY | PY | PY |
|  | **6.4 Were any systematic errors in measurement of the outcome related to intervention received?** | NI | PN | PN | PN | NI | NI | NI | NI | NI | NI | NI | NI | NI | NI | NI | NI | NI | NI |
|  | **Risk of bias judgement** | Mod | Mod | Mod | Mod | Mod | Mod | Mod | Serious | Mod | Mod | Mod | Mod | Mod | Mod | Mod | Mod | Mod | Mod |
| **Bias in selection of the reported result** | **Is the reported effect estimate likely to be selected, on the basis of the results, from...** | | | | | | | | | | | | | | | | | | |
|  | **7.1 multiple outcome *measurements* within the outcome domain?** | PN | PN | PN | PN | PN | PN | PN | PN | PN | PN | PN | PN | PN | PN | PN | PN | PN | PN |
|  | **7.2 multiple *analyses* of the intervention-outcome relationship?** | PN | PN | PN | PN | PN | PN | PN | PN | PN | PN | PN | PN | PN | PN | PN | PN | PN | PN |
|  | **7.3 different *subgroups*?** | NI | NI | NI | NI | NI | NI | NI | NI | NI | NI | NI | NI | NI | NI | NI | NI | NI | NI |
|  | **Risk of bias judgement** | Low | Low | Low | Low | Low | Low | Low | Low | Low | Low | Low | Low | Low | Low | Low | Low | Low | Low |
| **Overall bias** | | Serious | Serious | Serious | Serious | Serious | Serious | Mod | Serious | Serious | Serious | Serious | Mod | Mod | Serious | Mod | Serious | Serious | Serious |

# **Supplementary Information 5: Funnel plot**


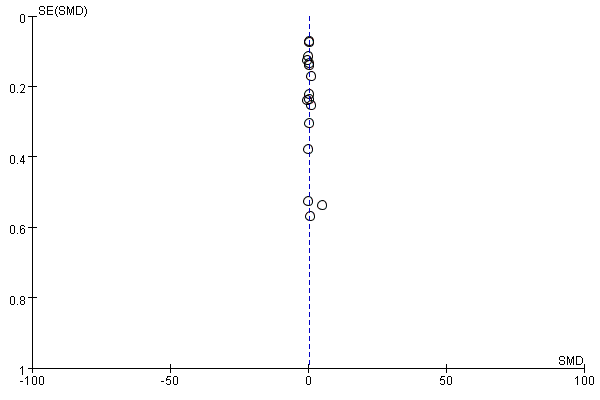


Figure 2 Funnel plot

*Funnel plot of capacity-building interventions on overall resilience


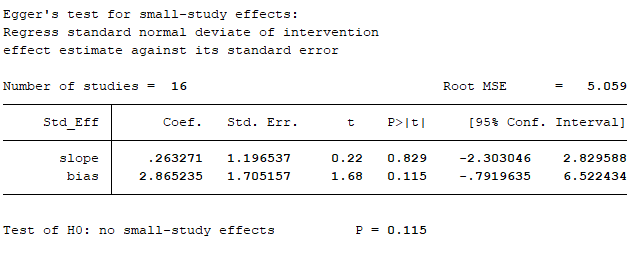


Figure 3 Egger’s test

# **Supplementary Information 6: Grade Assessment**

**Table 3 GRADE Assessment**

| **Certainty assessment** | | | | | | | **Number of patients** | | **Effect** | | **Certainty** | **Importance** |
| --- | --- | --- | --- | --- | --- | --- | --- | --- | --- | --- | --- | --- |
| **Number of studies** | **Study design** | **Risk of bias** | **Inconsistency** | **Indirectness** | **Imprecision** | **Other considerations** | **Capacity-building intervention** | **usual care at post intervention** | **Relative (95% *CI*)** | **Absolute (95% *CI*)** |  |  |
| **Mental health literacy at post-intervention (assessed with: Mental health literacy questionnaire, Mental health literacy scale, Mental health Literacy Scale Depression specific )** | | | | | | | | | | | | |
| 4 | randomised trials | serious^a^ | serious^b^ | not serious | not serious | none | 378 | 405 | - | SMD **1.7 SD higher** (0.57 higher to 2.84 higher) | ⨁⨁◯◯ Low | CRITICAL |
| **Resilience at post-intervention (assessed with: Healthy Kids Resilience Assessment, Resilience Youth Development Module, Chinese version of Resilience Scale, Connor-Davidson Resilience Scale 10, Child and Youth Resilience Measure 12, Korean Adolescent Resilience Scale, Resiliency Scales for Children and Adolescents, Gürgan Resilience scale, Resilience Scale 14, Social-Emotional Assets and Resilience Scales, )** | | | | | | | | | | | | |
| 16 | randomised trials | serious^c^ | serious^d^ | not serious | serious^e^ | none | 1845 | 1465 | - | SMD **0.51 SD higher** (0.26 higher to 0.76 higher) | ⨁◯◯◯ Very low | CRITICAL |
| **Depression at post-intervention (assessed with: Patient Health Questionnaire-9, Self-rating Depression Scale, Mood and Feelings Questionnaire,Chinese, Reynolds Adolescent Depression Scale, version of the Center for Epidemiologic)** | | | | | | | | | | | | |
| 6 | randomised trials | not serious | serious^f^ | not serious | serious^g^ | none | 976 | 552 | - | SMD **0.31 SD lower** (0.64 lower to 0.01 higher) | ⨁⨁◯◯ Low | CRITICAL |
| **Anxiety at post-intervention (assessed with: General Anxiety Disorder-7, Self-rating Anxiety Scale, Scale for Childhood Anxiety and Related Disorders )** | | | | | | | | | | | | |
| 4 | randomised trials | not serious | serious^h^ | not serious | serious^i^ | none | 804 | 438 | - | SMD **0.15 SD lower** (0.57 lower to 0.27 higher) | ⨁⨁◯◯ Low | CRITICAL |

**CI:** confidence interval; **SMD:** standardised mean difference

#### Explanations

a. All studies showed some concerns of bias due to no blinding to outcome assessors (self-reported outcome measures), and Zare et al 2021 and Campos et al 2018 showed some risk of concern due to randomized process.

b. High statistical heterogeneity may be due to the inclusion of Zare et al 2021 and Liddle et al 2019, both of which focused on single-gender populations.

c. Six out of 16 studies had high risks of bias in Rob 2 assessment

d. High statistical heterogeneity (*I^2^* = 89%).

e. Felver et al 2018, Hyun et al 2010, and Johnstone et al 2020, had a wide CI

f. High statistical heterogeneity (*I*^2^=83%)

g. Hyun et al 2010 and Maaloufadi et al 2020 had wide CI

h. High statistical heterogeneity (*I*^2^=83%)

i. Berger et al 2018, Maaloufadi et al 2020 and Zhang et al 2021 had wide CI

# **Supplementary Information 7:** **Presentation of deviations from the preregistration**

Table 4 Presentation of deviations from the preregistration

| **Category** | Pre-registration | Modified | Reasons for changes |
| --- | --- | --- | --- |
| **Title** | A systematic review of building adolescent's capacity to promote mental health | Strengths-based capacity-building interventions to promote adolescents' mental health: A systematic review and meta-analysis | We believe that including our theoretical framework (Strengths-based intervention) in the title will make it easier for readers to search for our work. |
| **Search** | Studies published between January 2003 and October 2023 will be searched. | Studies published from their inceptions to June 2024 have been searched. We searched the following electronic electronic databases: PubMed, EMBASE, PsycINFO, CINAHL, The Cochrane Library (Cochrane Database of Systematic Reviews), and CNKI (China National Knowledge Infrastructure). Both English and Chinese published were searched. | We acknowledge that as the review progressed, certain refinements were made to our search strategy and inclusion criteria. These refinements were not promptly updated in the pre-registered record. However, the core objectives and scope of the review remained unaltered.  In our manuscript, we furnished more detailed information regarding our search approaches. For instance, we utilized the PICO framework, incorporated additional databases and trial registries, and extended the search timeline until June 2024. This was done to ensure that we captured the most current evidence available. As a result, there was a delay in the originally scheduled completion time, causing the entire process to fall behind the preregistered schedule. |
| **Study design** | Inclusion criteria: Studies using randomized controlled design. Exclusion criteria: Non-randomized controlled trial and other quantitative and qualitative (exploratory, descriptive and comparative) study designs | Randomized controlled studies; and quasi- and non-randomized experimental studies | The inclusion criteria were adjusted in order to adopt more inclusive search strategies, as we believed that including both types of studies would allow us to capture the full breadth of strength-based capacity-building interventions in target population (adolescents). |
| **Population** | Exclusion criteria: Adolescents who are clinically diagnosed with mental disorder | Exclusion criteria: Adolescents who had pre-existing clinically diagnosed mental health disorders, cognitive impairments and/or learning disabilities; adolescents who were receiving or had recently received psychiatric and/or psychological treatments; and adolescents with serious physical illnesses at recruitment | The exclusion of Population were expanded to ensure a more focused and relevant population. We recognized that including adolescents with complex clinical needs might introduce some confounding factors. So this adjustments aimed to better understand the impact of strength-based capacity-building interventions among adolescents without severe clinical conditions. |

Table 4 (Continued)

| **Category** | Pre-registration | Modified | Reasons for changes |
| --- | --- | --- | --- |
| **Intervention(s) or exposure(s)** | Inclusion criteria: Intervention aiming to promote adolescent mental health through build capacity from mental health literacy, resilience, self-efficacy, and positive thinking; Intervention focuses on building these capacities at adolescent level | Inclusion criteria: (1) an interventional study; (2) used an intervention targeted at enhancing adolescent capacities to maintain and promote mental health in areas of mental health literacy, resilience, self-efficacy, and/or positive thinking and (3) included a control or a comparison group, which involved either no intervention, waitlist control, or active controls that did not specifically target adolescent mental health literacy, resilience, self-efficacy, and/or positive thinking. | We refined the descriptions in our manuscript to provide more details. |
| **Main outcomes** | Inclusion criteria: The primary outcomes of the intervention are the increase in the awareness of mental health and positive attitudes towards mental health, behavior in change such as positive coping skills or strategies (using validated coping scale or measurement), the level of perceived self-efficacy, personal empowerment, and resilience. The secondary outcomes are symptoms or specific problem-related outcome, including the level of depression, anxiety, suicidality, risky behaviors or other behavioral problems. Exclusion criteria: Studies that measure only symptom or problem-specific outcomes are excluded. | The primary outcomes were mental health literacy, resilience, self-efficacy, and positive thinking. The secondary outcomes were common psychological symptoms such as depression and anxiety and related behavioral problems. | In the pre-registration of the review, we broadly defined the primary outcomes as awareness of mental health, positive attitudes, and behavior changes such as positive coping, self-advocacy, and resilience. However, after further review of the literature, we refined our primary outcomes to mental health literacy, resilience, self-efficacy and positive thinking. This refinement was made because we believe that these four capacities mental health literacy, resilience, self-efficacy, and positive thinking were both well-established in the literature and most relevant to the conceptual framework of strength-based capacity-building for adolescents. |
| **Risk of bias (quality) assessment** | Cochrane risk of bias tool will be used to assess internal validity of included studies | The risk of bias in the included studies was independently evaluated by two reviewers using Version 2 of the Cochrane risk of bias tool (RoB 2) for randomized controlled trials (RCTs) and cluster randomized controlled trials (c-RCTs). The quasi-experimental design was assessed using Risk Of Bias In Non-randomized Studies – of Interventions (Robins-I). | We did not make major changes to our approach to assessing risk of bias. However, as we progressed, we identified different types of randomized controlled trials within our included studies. We followed the Cochrane guidelines to ensure an appropriate and consistent assessment of risk of bias for different types of studies and reported these in detail in our manuscript. |

Table 4 (Continued)

| **Category** | Pre-registration | Modified | Reasons for changes |
| --- | --- | --- | --- |
| **Strategy for data synthesis** | Dichotomous outcomes will be presented as risk ratios (RR) with 95% confidence intervals (CI), while continuous data will be expressed as mean differences (MD) with 95% CI. In cases where outcome measures have differing scales, we will calculate the standardized mean difference (SMD) with its 95% CI. Heterogeneity will be assessed using the χ² test and the I² statistic. | All of the analyses were conducted using Review Manager 5.4. Data from three or more studies reporting the same outcomes were combined for meta-analysis in this review.  The effect sizes was estimated using the standardized mean difference (SMD) between the intervention and comparison groups regarding the change in mean values from the baseline to the endline after standardization by their pooled standard deviations (SDs), along with the 95% confidence intervals (CIs). Effect sizes were calculated based on the means and SDs. When studies did not provide these data, we contacted the authors directly. SMDs of 0.2, 0.5, and 0.8 are considered small, medium, and large effect sizes, respectively. When SDs were not provided and could not be obtained from the author, they were estimated using alternative statistics, such as P-values or t-values. We pooled the effect estimates using a random effects model because of the variability between studies. Heterogeneity was assessed using the *I²* statistic and categorized as low (*I²* < 40%), moderate (40% ≤ *I²* ≤ 75%), or high (*I²* > 75%).  The c-RCTs were adjusted for clustering effects using the design effect formula: 1 + (M − 1) × ICC. If the ICC was not reported, an ICC of 0.05 was chosen based on prior studies.  Leave-one-out sensitivity analysis was performed by removing one study at a time to examine the robustness of the pooled effect. | We did not make any major changes to the principles of our data analysis strategy. However, we recognized the need to provided more details in our data synthesis strategy due to the different types of studies included. For example, we followed Cochrane guidelines and provided a more detailed explanation in the manuscript regarding how cRCTs and RCTs were handled. In our manuscript, we provided more detailed explanations on how we assessed these data to ensure the rigor and transparency of the process. |
| **Analysis of subgroups or subsets** | No description. | When there was significant heterogeneity in the study, subgroup analyses were conducted based on the type of RCT, age group, and different theoretical foundations. | We refined our subgroup analysis approach as the review progressed and this was based on the available data and relevant characteristics observed in the studies. We provided in details how these subgroups were analysed in our manuscript. |
